# Bioactivity-Guided Isolation of Antistroke Compounds from *Gymnadenia conopsea* (L.) R. Br.

**DOI:** 10.3390/molecules29184389

**Published:** 2024-09-15

**Authors:** Juan Qin, Shiyi Xue, Chao Xu, Jian Jin, Jianbin Wang, Hailian Yuan, Liang Liu

**Affiliations:** 1 Institute of Translational Medicine, Medical College, Yangzhou University, Yangzhou 225009, China; qinjuan0324@163.com (J.Q.); shiyixue0430@163.com (S.X.); 007898@yzu.edu.cn (J.W.); y15253090921@163.com (H.Y.); 2 Jiangsu Key Laboratory of Integrated Traditional Chinese and Western Medicine for Prevention and Treatment of Senile Diseases, Yangzhou University, Yangzhou 225009, China; 3 College of Animal Science and Technology, Yangzhou University, Yangzhou 225009, China; zhongchaoxu@163.com (C.X.); dz120210008@yzu.edu.cn (J.J.)

**Keywords:** *Gymnadenia conopsea*, bioactivity-guided separation strategy, antistroke effect, *Col27a1* gene

## Abstract

A bioactivity-guided separation strategy was used to identify novel antistroke compounds from *Gymnadenia conopsea* (L.) R. Br., a medicinal plant. As a result, 4 undescribed compounds (**1–2**, **13**, and **17**) and 13 known compounds, including 1 new natural product (**3**), were isolated from *G. conopsea.* The structures of these compounds were elucidated through comprehensive spectroscopic techniques, such as 1D/2D nuclear magnetic resonance (NMR) spectroscopy, high-resolution electrospray ionization mass spectrometry (HRESIMS), and quantum chemical calculations. An oxygen–glucose deprivation/reoxygenation (OGD/R)-injured rat pheochromocytoma (PC12) cell model was used to evaluate the antistroke effects of the isolates. Compounds **1–2**, **10–11**, **13–15**, and **17** provided varying degrees of protection against OGD/R injury in the PC12 cells at concentrations of 12.5, 25, and 50 µM. Among the tested compounds, compound **17** demonstrated the most potent neuroprotective effect, which was equivalent to that of the positive control drug (edaravone). Then, transcriptomic and bioinformatics analyses were conducted to reveal the regulatory effect of compound **17** on gene expression. In addition, quantitative real-time PCR (qPCR) was performed to verify the results of the transcriptomic and bioinformatics analyses. These results suggest that the in vitro antistroke effect of compound **17** may be associated with the regulation of the *Col27a1* gene. Thus, compound **17** is a promising candidate for the development of novel antistroke drugs derived from natural products, and this topic should be further studied.

## 1. Introduction

*Gymnadenia conopsea* (L.) R. Br., a member of the family Orchidaceae, is a perennial herbaceous flowering plant that is distributed at altitudes from 200 to 4700 m throughout Northern Europe and the temperate and subtropical zones in Asian countries [1]. This plant has long been used as a valuable Tibetan medicine in China. The famous Tibetan Materia Medica book called Jingzhu Bencao describes the use of *G. conopsea* to boost vitality and prolong life [2]. The medicinal part of *G. conopsea* is the tuber, which is renowned for its palm-like appearance and characteristic finger-like protrusions [3].

At present, approximately one hundred chemical compounds, including low-molecular-weight organic acids, benzylester glucosides [4,5,6,7], dihydrostilbenes [8], phenanthrenes [8], and alkaloids [9,10], have been reported from *G. conopsea* [11]. These various types of secondary metabolites determine the diverse biological activities of *G. conopsea*. Extracts of *G. conopsea* have been reported to have a remarkable array of pharmacological effects, including antifatigue, antioxidant, antiviral, sedative–hypnotic, and immunoregulatory effects [1]. However, whether *G. conopsea* has other pharmacological effects remains unknown. Moreover, although we have a certain understanding of its components, there is considerable investigation space for the phytochemical aspects of *G. conopsea*. In recent years, the use of *G. conopsea* has increasingly expanded because of its health care properties [1]. Thus, further investigations on *G. conopsea* are urgently needed to determine its multiple pharmacological effects and active compounds and to improve the application of this valuable plant.

*G. conopsea* belongs to the subfamily Orchidoideae of the family Orchidaceae. *Gastrodia elata* Bl. is also a member of the subfamily Orchidoideae and the tuber of *G. elata*, a famous Chinese medicine, has been reported to exhibit antistroke activity [12]. The principles of plant chemical taxonomy reveal that closely related species have similar chemical constituents and bioactivities [13]. Additionally, the previous phytochemical studies have demonstrated that both *G. elata* and *G. conopsea* contain organic acids, benzylester glucosides, dihydrostilbenes, phenanthrenes, and alkaloids [4,5,6,7,8,9,12]. Inspired by the above principles and related phytochemical findings, we hypothesize that *G. conopsea* may also exhibit antistroke activity.

In the present study, a bioactivity-guided separation strategy was used to isolate the constituents of *G. conopsea*, and the antistroke effects of the isolated compounds were evaluated. The novel compound with the most promising antistroke effect was subsequently examined through transcriptome analysis to investigate its regulatory effects on gene expression. Then, quantitative real-time PCR (qPCR) was performed to verify the results of the transcriptome analysis. Overall, this comprehensive approach provides insights into the potential antistroke property of *G. conopsea* and offers useful data for its further development and utilization.

## 2. Results

### 2.1. Identification of the Active Fractions

**Figure 1:**
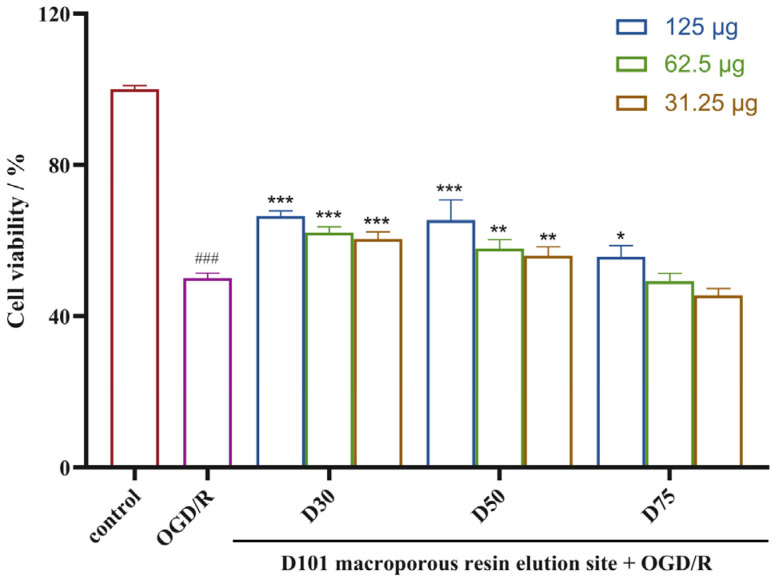
Neuroprotective effects of samples prepared from *Gymnadenia conopsea* on ODG/R-induced PC12 cells (means ± SDs, *n* = 3). ^###^ *p* < 0.001 versus the control, * *p* < 0.05, ** *p* < 0.01, and *** *p* < 0.001 versus OGD/R.

We first evaluated the neuroprotective activities of the fractions to identify the active fractions. The effects of each fraction on oxygen–glucose deprivation/reoxygenation (OGD/R)-injured rat pheochromocytoma (PC12) cells are illustrated in Figure 1. The neuroprotective effects of the 30% ethanol fraction (D30) and the 50% ethanol fraction (D50) surpassed that of the 75% ethanol fraction (D75). Consequently, we systematically separated the compounds within the D30 and D50 fractions.

### 2.2. Identification of the Structures

A total of 4 new compounds (**1–2**, **13**, and **17**) and 13 known compounds, including 1 new natural product (**3**), were isolated from *G. conopsea* (Figure 2), and their structures were elucidated through comprehensive spectroscopic techniques, such as 1D/2D nuclear magnetic resonance (NMR) spectroscopy, high-resolution electrospray ionization mass spectrometry (HRESIMS), and quantum chemical calculations. The key heteronuclear multiple bond connectivity (HMBC), correlation spectroscopy (COSY), and nuclear Overhauser effect spectroscopy (NOESY) correlations of the four new compounds are shown in Figure 3. The stereoscopic configuration of compound **17** is shown in Figure 4. The ultraviolet (UV), infrared (IR), circular dichroism (CD), HRESIMS, 1D NMR, and 2D NMR spectra of the isolates are shown in Figures S1–S78.

Compound **1** was obtained as a pale yellow powder, and its molecular formula was determined to be C_20_H_24_O_8_ with nine degrees of unsaturation according to its HRESIMS data ([M + Na]^+^ *m*/*z* 415.1363, calcd 415.1363). The ^1^H-NMR spectrum of **1** (Figure S5) exhibited signals for one methyl singlet (*δ*_H_ 2.11), two methylene groups (including one methylene in glucose), six olefinic protons, and five glycosyl protons. The ^13^C-NMR data (Figure S6) revealed one methyl group, two methylene groups, eleven methine groups (including six olefinic carbons), and six quaternary carbons (olefinic carbons). The ^1^H-NMR and ^13^C-NMR data revealed a pair of overlapping AA′BB′ signals. In the HMBC spectrum, the correlations from H-7′ to C-1′, C-2′, and C-6′ indicated the presence of a 1′,4′-disubstituted benzyl moiety. The HMBC correlations from H-7′ to C-5, C-4, and C-6, from H-6 to C-5, C-1, C-2, C-4, and C-7′, from H-3 to C-5, C-1, C-2, and C-4, and from H-1″ to C-1 indicate the presence of an additional 1,2,4,5-tetrasubstituted phenyl moiety in **1**, which was connected to the 1′,4′-disubstituted benzyl moiety. In the ^13^C-NMR spectrum, six carbon signals (*δ*_C_ 104.7, 74.9, 77.7, 71.1, 78.1, and 62.1) assigned to a glucopyranosyl moiety were observed. Additionally, in the ^1^H-NMR spectrum, terminal protons were observed at *δ*_H_ 4.63 (d, *J* = 7.2 Hz, H-1″), and overlapping signals were observed between *δ*_H_ 3.27 and 3.78, indicating the presence of a glucose moiety and suggesting a *β*-glycosidic linkage. The D-glucopyranose moieties of **1** were defined by comparison with those of a standard sample via silica gel thin layer chromatography (TLC) analysis after acid hydrolysis. The HMBC correlations from H-1″ to C-1 confirmed that the sugar unit was connected to the 1,2,4,5-tetrasubstituted phenyl moiety. Thus, the structure of compound **1** was fully elucidated, and the compound was named 2-hydroxy-1-[(4-hydroxyphenyl) methyl]-4-methylphenyl-1-*β*-D-glucopyranoside.

Compound **2** was obtained as a pale yellow powder, and its molecular formula was determined to be C_20_H_24_O_7_ with nine degrees of unsaturation on the basis of its HRESIMS data ([M + Na]^+^ *m*/*z* 399.1409, calcd 399.1414). The ^1^H-NMR spectrum of **2** (Figure S15) exhibited signals for one methyl singlet (*δ*_H_ 2.16), two methylene groups (including one methylene in glucose), seven olefinic protons, and five glycosyl protons. The ^13^C-NMR data (Figure S16) revealed one methyl group, two methylene groups, twelve methine groups (including seven olefinic carbons), and five quaternary carbons (olefinic carbons). The ^1^H-NMR and ^13^C-NMR data revealed a pair of overlapping AA′BB′ signals. In the HMBC spectrum, the correlations from H-7′ to C-1′, C-2′, and C-6′ and from H-1″ to C-4′ indicated the presence of a 1′,4′-disubstituted benzyl moiety. In the ^13^C-NMR spectrum, six carbon signals (*δ*_C_ 102.5, 75.0, 78.0, 71.4, 78.1, and 62.5) assigned to a glucopyranosyl moiety were observed. In the HMBC spectrum, the correlations from H-1″ to C-4′ indicated that a sugar unit was connected to the 1′,4′-disubstituted benzyl moiety. In the TLC analysis after acid hydrolysis, the *β*-D-glucopyranose part of **2** was confirmed by comparison with the standard sample. The HMBC correlations from H-7′ to C-1, C-6, and C-2, from H-4 to C-2 and C-6, from H-3 to C-5, C-1, and C-2, from H-6 to C-4, C-2, and C-7′, and from CH_3_ (*δ*_H_ 2.16, s) to C-5, C-4, and C-6 indicated the presence of a 1,2,5-trisubstituted phenyl moiety in **2**. The HMBC correlations from H-7′ to C-1, C-6, and C-2 indicated that the 1,2,5-trisubstituted phenyl moiety was connected to a 1′,4′-disubstituted benzyl moiety. Thus, the structure of compound **2** was fully elucidated, and the compound was named 2-hydroxy-5-methylphenyl-1-(4-*β*-D-glucopyranosyloxybenzyl).

Compound **13** was obtained as a white amorphous powder, and its molecular formula was determined to be C_12_H_11_N_4_O_2_ with eight degrees of unsaturation on the basis of its HRESIMS data ([M + H]^+^ *m*/*z* 243.0871, calcd 243.0877). The ^1^H and ^13^C-NMR data (Figures S24 and S25) indicate that this compound and 9-benzylhypoxanthine exhibit a high degree of similarity [14]. Compound **13** has an additional hydroxyl group at the C-4′ position. This assignment was confirmed by the HMBC cross peaks from H-7′ to C-1′, C-2′, and C-6′, from 4′-OH to C-4′, C-3′, and C-5′, from H-2 to C-4 and C-6, and from H-8 to C-4 and C-5. Consequently, the structure of compound **13** was identified, and it was named 9-*p*-hydroxybenzylhypoxanthine.

**Figure 4:**
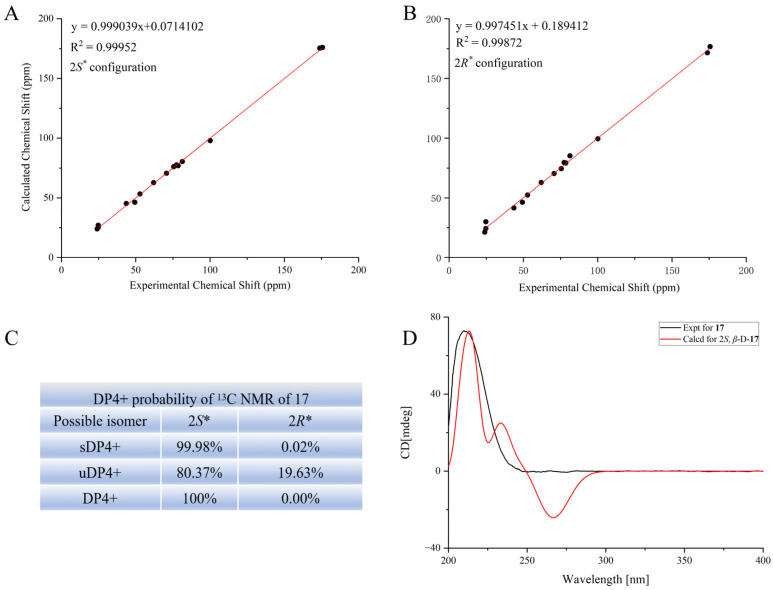
Stereoscopic configuration of compound **17**. (**A**) Correlations between the calculated and experimental chemical shifts of 2*S*. (**B**) Correlations between the calculated and experimental chemical shifts of 2*R*. (**C**) DP4+ probabilities of possible isomers of **17**. (**D**) Experimental ECD spectrum of **17** (black line) and the calculated spectrum of **17** (red line).

Compound **17** was obtained as a colorless transparent gum (MeOH). Its molecular formula was determined to be C_15_H_26_O_10_ with three degrees of unsaturation on the basis of its HRESIMS data ([M + Na]^+^ *m*/*z* 389.1423, calcd 389.1418). The ^1^H and ^13^C-NMR data (Figures S34 and S35) indicate that this compound and (2R)-2-(*β*-D-glucopyranosyloxy-2-(2-methylpropyl) butanedioic acid exhibit a high degree of similarity [15]; however, a hydroxycarbonyl group (C-4) in (2*R*)-2-(*β*-D-glucopyranosyloxy-2-(2-methylpropyl) butanedioic acid was replaced by a methoxycarbonyl group in **17**. This assignment was confirmed by the HMBC cross peaks from H-9 to C-4 and from H-3 to C-4, C-2, C-1, and C-5. A NOESY experiment was performed to determine the relative configuration of compound **17**. Unfortunately, no effective NOESY cross peak was observed. Next, the configuration of C-2 was confirmed through NMR calculations. The results showed that S* (R2 = 0.99952) (Figure 4A) is more consistent with the experimental values than R* (R2 = 0.99872) (Figure 4B), which is further supported by DP4+ (Figure 4C) and the calculated ECD (Figure 4D). According to the DP4+ analysis, the probability of 2S* is 100%, and the calculated ECD spectrum of 2S* matches well with the experimental ECD spectrum, indicating that C-2 has an *S* configuration. At this point, the structure of compound **17** was fully elucidated, and the compound was named (2*S*)-2-(*β*-D-glucopyranosyloxy)-2-(2-methylpropyl) butanedioic acid 4-methyl ester.

By comparing the spectroscopic data from the isolated compounds with the data reported in the literature, the other 13 known compounds were identified as 4-[[[(2*E*)-3-(4-hydroxy-3-methoxyphenyl)-1-oxo-2-propen-1-yl]oxy]methyl]phenyl *β*-D-glucopyranoside (**3**) [16], 4-hydroxy-trans-cinnamomic acid 4-*β*-D-glucopyranosyloxybenzyl ester (**4**) [17], 4-O-(6′-O-glucosyl-p-coumaroyl)-4-hydroxybenzy alcohol (**5**) [18], amburoside B (**6**) [19], 4-*β*-D-glucopyranosyloxybenzyl ester (**7**) [17], vanillic acid (**8**) [20], 4-hydroxybenzaldehyde (**9**) [21], 4-methylphenyl *β*-D-glucopyranoside (**10**) [22], 4-(methoxymethyl) phenyl-1-O-*β*-D-glucopyranoside (**11**) [23], 4-(*β*-D-glucopyranosyloxy) benzyl ethyl ether (**12**) [24], 6-(ethoxymethyl) pyridin-3-ol (**14**) [25], 1-(2,3-dihydroxyphenyl)pyrrolidin-2-one (**15**) [26], and N^6^-(4-hydroxybenzyl)-adenosine (**16**) [24].

### 2.3. Neuroprotective Activity Results

**Figure 5:**
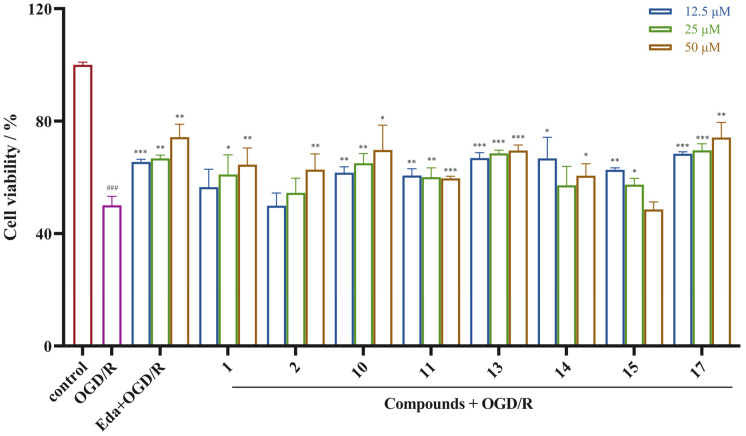
Neuroprotective effects of the isolated compounds on ODG/R-induced PC12 cells (means ± SDs, *n* = 3). ^###^ *p* < 0.001 versus the control, * *p* < 0.05, ** *p* < 0.01, and *** *p* < 0.001 versus OGD/R. Positive control: edaravone (Eda).

After systematic separation, the antistroke activity of the 17 isolated compounds was evaluated. The PC12 cell line possesses typical neuron features and has been extensively used in related neurological studies [27,28]. Therefore, we used PC12 cells to establish OGD/R-induced PC12 cell damage model to mimic the ischemia and reperfusion process of stroke and to investigate their in vitro neuroprotective activities [28]. The results revealed that compounds **1**–**2**, **10**–**11**, **13**–**15**, and **17** had varying degrees of protective effects on the OGD/R-injured PC12 cells at concentrations of 12.5, 25, and 50 µM (Figure 5). Notably, compound **17** demonstrated a potent neuroprotective effect. The positive control, edaravone, exhibited protective effect on the OGD/R-injured PC12 cells, with cell survival rates of 65.47%, 66.71%, and 74.31% at concentrations of 12.5 μM, 25 μM, and 50 μM, respectively, and those of compound **17** were 68.39%, 69.62%, and 74.16%, respectively, indicating that compound **17** exhibited neuroprotective effect comparable to that of edaravone. The above results indicate that compound **17** is a promising antistroke drug candidate.

### 2.4. Transcriptomic and Bioinformatic Analyses and qPCR Experiments Results

**Figure 6:**
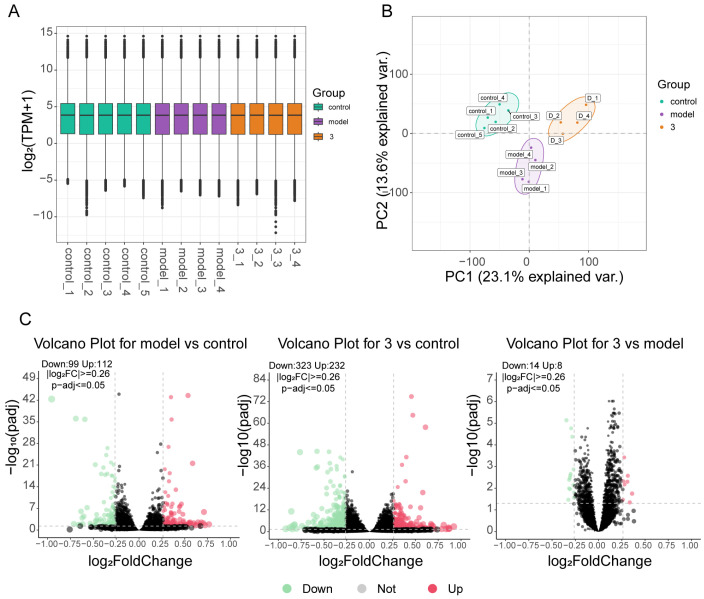
Differentially expressed gene (DEG) analysis. (3 = compound **17**) (**A**) Box plot of log_2_ (TPM) values for mRNA under different conditions. (**B**) PCA diagram of normalized mRNA expression values illuminating the general relationship between datasets. (**C**) Upregulated and downregulated genes in the mRNA database among the three groups. The green dots indicate downregulated genes, and the red dots indicate upregulated genes.

Transcriptome sequencing analysis was performed to further elucidate the protective effect of compound **17** on the OGD/R-injured PC12 cells and to determine the variations in transcriptome levels among the normal group, model group, and treatment group. Box plot distributions of transcripts per million (TPM) depict the median and quartile values of mRNA expression across different groups (Figure 6A). Principal component analysis revealed that the distinct groups could be readily distinguished, suggesting that the transcriptional profile of PC12 cells was altered by OGD/R and drugs (Figure 6B). Additionally, differential gene expression analysis was conducted. The results revealed that compared with the normal group, the model group contained 99 downregulated genes and 112 upregulated genes. Furthermore, compared with the normal group, the treatment group contained 323 downregulated genes and 232 upregulated genes, and when the treatment group was compared with the model group, 14 downregulated genes and 8 upregulated genes were observed (Figure 6C). 

**Figure 7:**
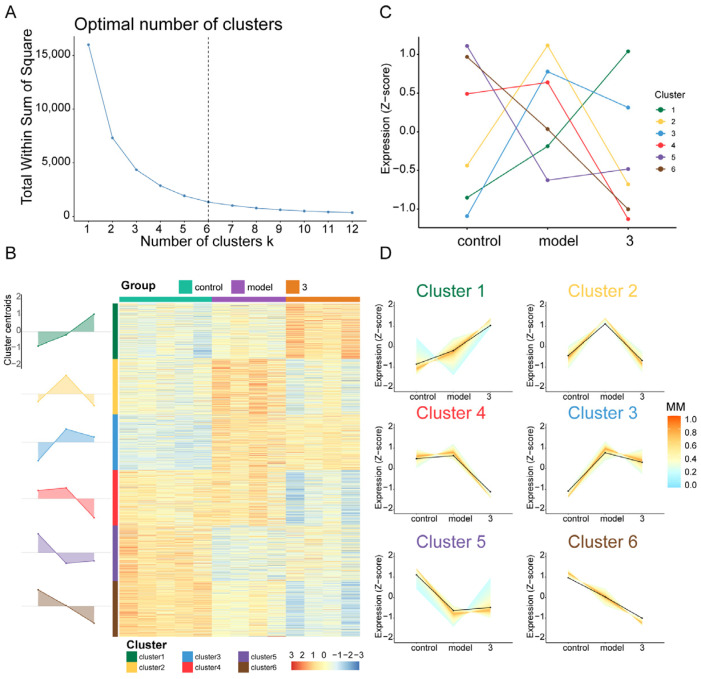
Global changes in gene expression for multiple time points. (3 = compound **17**) (**A**) Optimal number of clusters. (**B**) Line plot displaying the expression patterns of mRNAs and cluster centroids identified by the fuzzy c-means algorithm at different developmental time points. (**C**) Heatmap displaying six obtained clusters with dynamic gene expression patterns. (**D**) The clusters’ overall gene expression dynamics are displayed as area plots (visualized in relation to cluster centroids).

**Figure 8:**
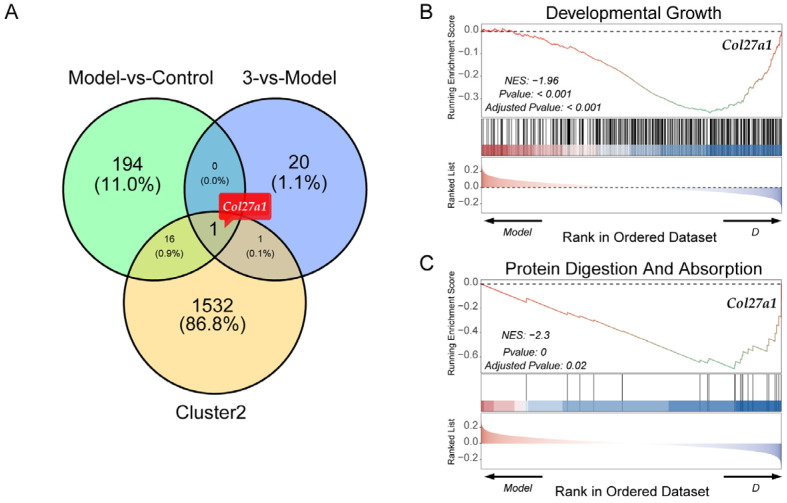
Identification of key genes. (3 = compound **17**) (**A**) Key genes identified via a Venn diagram. (**B**,**C**) GSEA enrichment analysis showing that DEGs are significantly enriched in the developmental growth pathway (**B**) and protein digestion and absorption (**C**).

**Figure 9:**
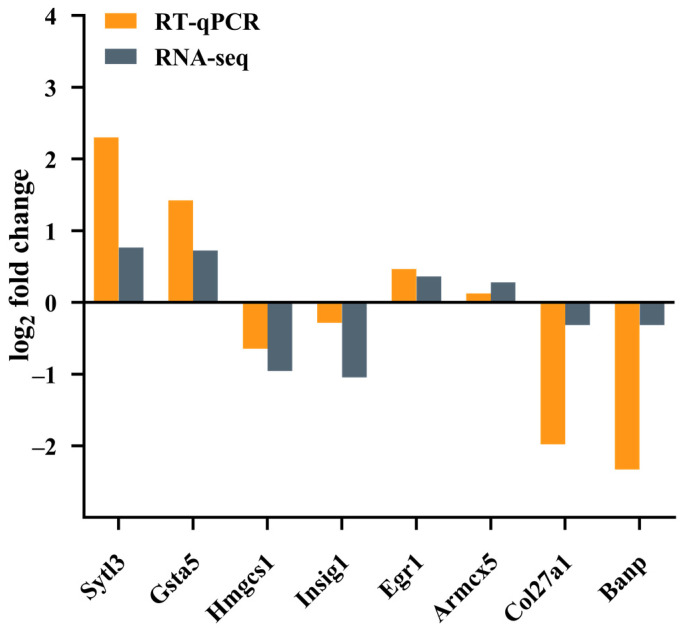
Comparisons of the expression patterns of *Sytl3*, *Gsta5*, *Hmgcs1*, *Insig1*, *Egr1*, *Armcx5*, *Col27a1*, and *Banp* obtained via qPCR and those obtained via RNA-seq.

Next, clustering analysis was performed to describe the widespread patterns and trends of transcriptional changes. Based on the scaled and centralized mean expression values obtained, we applied the fuzzy c-means clustering algorithm to the protein-coding genes and determined the optimal number of clusters (k = 6) through gap statistics (Figure 7A). Heatmaps and line plots depict the dynamic transcriptional features of the normal group, model group, and treatment group (Figure 7B). Among the six clusters, significant differences were observed in the increase in genes in cluster 2 after modeling and the decrease in genes in cluster 2 after treatment (Figure 7C,D). Based on the GO enrichment analysis and KEGG pathway enrichment analysis of the gene cluster, GO enrichment analysis of cluster 2 focused primarily on processes such as proteasome-mediated ubiquitin-dependent protein catabolic processes, protein acylation, the endoplasmic reticulum membrane, histone modification, and DNA replication. KEGG enrichment analysis revealed that the enriched genes were related mainly to the cell cycle, endoplasmic reticulum protein processing, and nuclear transport. Next, by taking the intersection of cluster 2 genes and DEGs, we identified the key gene *Col27a1* (Figure 8A). To verify the reliability of the RNA sequencing (RNA-Seq) results, qPCR was used to validate the differentially expressed genes (*Col27a1*, *Banp*, *Hmgcs1*, *Insig1*, *Sytl3*, *Gsta5*, *Egr1*, and *Armcx5*). The qPCR results were similar to the RNA-Seq results, indicating the reliability of the RNA-Seq results (Figure 9). A literature review revealed that *Col27a1* is regulated by *SOX9* [29,30], but its role in OGD/R must be further investigated. According to the GO and KEGG enrichment analyses of the *Col27a1* gene, we may focus on pathways related to developmental growth and protein digestion and absorption in the subsequent studies (Figure 8B,C).

Overall, the present study has enriched the pharmacological and phytochemical studies of *G. conopsea.* For the first time, *G. conopsea* was found to have an in vitro antistroke effect, which offers a new perspective to better understand the pharmacological actions of this medicinal plant and reveals more possibilities for its further applications. A total of 4 undescribed compounds, along with 13 known compounds, were isolated and identified. These compounds are phenolic glycosides, alkaloids, and organic acid glycosides, and this finding supports the previous phytochemical results of *G. conopsea* [11]. Eight compounds exhibited protective effects on the OGD/R-injured PC12 cells at the tested concentrations. More importantly, the activity of compound **17** was comparable to that of edaravone. Transcriptomic and bioinformatic analyses, along with qPCR experiments, indicate that the in vitro antistroke effect of compound **17** may be associated with the regulation of the *Col27a1* gene. However, the related mechanisms need further investigation. Structural modification and target confirmation studies are needed to identify the lead compounds based on compound **17** for novel drug development. Overall, our studies highlight the medicinal value of *G. conopsea* and provide a theoretical basis for its further development and utilization.

## 3. Materials and Methods

### 3.1. General Experimental Procedures

The 1D and 2D NMR data were measured on a Bruker AVANCE 600 (Bruker, Billerica, MA, USA) spectrometer using TMS as an internal standard. HRESIMS analyses were performed on a MaXis quadrupole time-of-flight mass spectrometer (Bruker, Billerica, MA, USA). UV spectra, IR spectra, CD spectra, and optical rotations were recorded on a Shimadzu U-3900 spectrometer (Shimadzu, Kyoto, Japan), Varian Cary 610/670 IR spectrometer (Varian, Palo Alto, CA, USA), J-810 Circular Dichroism spectrapolarimeter (JASCO, Tokyo, Janpan), and WZZ-2B automatic polarimeter (Shanghai Sincere Dedication of Science and Technology Innovation, Shanghai, China), respectively. Column chromatography (CC)-based separations were performed using a Sephadex LH-20 column (Cytiva, Uppsala, Sweden), silica gel (Qingdao Marine Chemistry Ltd., Qingdao, China), and an ODS C18 column (Merck, Darmstadt, Germany). Semipreparative high-performance liquid chromatography (HPLC) separations were performed via an Agilent 1260 Infinity II (Agilent Technologies, Palo Alto, CA, USA) instrument equipped with a Cosmosil ODS column and a DAD detector. All the solvents used were of analytical grade (Sinopharm Chemical Reagent Co., Ltd., Shanghai, China).

### 3.2. Plant Material

Tubers of *G. conopsea* were collected from Tibet, China, in March 2020 and identified by Prof. Liang Liu, one of the authors. A voucher specimen (SZS20200328) was deposited at the Medical College of Yangzhou University.

### 3.3. Extraction and Isolation

The dried tubers of *G. conopsea* (20.0 kg) were ground into a powder and extracted three times with aqueous ethanol (EtOH) (95%, *v*/*v*). The combined extract was filtered and concentrated under reduced pressure to generate a residue (1.0 kg), which was separated via a D101 macroporous resin (20 kg/30 BV) CC, with a flow rate of approximately 2–3 BV/h. Gradient elution was carried out using 30% ethanol, 50% ethanol, and 75% ethanol, yielding the following fractions: D30 (Fr. 1, 231.4 g), D50 (Fr. 2, 78.8 g), and D75 (Fr. 3, 78.3 g). Fr. 1 was subjected to silica gel (200–300 mesh) CC using a dichloromethane–methanol gradient (50:1 to 0:1) to yield 9 fractions (Fr. 1.1 to Fr. 1.9). After Fr. 1.3 was recrystallized, compound **8** (10.5 mg) was obtained. Fr. 1.4 was subjected to several purification processes, including Sephadex LH-20 CC and ODS CC, purified by semipreparative HPLC, and eluted with ACN/H_2_O (3:17) to obtain compounds **14** (4.7 mg) and **15** (3.1 mg). Then, several purification processes were performed on Fr. 1.7, including silica gel CC (200–300 mesh, silica gel H), ODS CC, and Sephadex LH-20 CC, resulting in the isolation of compounds **10** (5.7 mg), **11** (9.5 mg), **12** (30.6 mg), **13** (1.0 mg), and **16** (2.0 mg). Subsequently, semipreparative HPLC purification was performed using ACN/H_2_O (1:4) as the elution solvent, yielding compound **17** (21.3 mg). Fr. 2 was subjected to silica gel (200–300 mesh) CC with a dichloromethane–methanol gradient (50:1 to 0:1), generating nine fractions (Fr. 2.1 to Fr. 2.9). Fr. 2.1 was subjected to ODS CC with a methanol–water gradient (1:9 to 1:0), resulting in the isolation of four subfractions (Fr. 2.1.1 to Fr. 2.1.4). Fr. 2.1.2 was further purified through semipreparative HPLC with ACN/H_2_O (1:4) as the elution solvent, yielding compound **9** (7.5 mg). Fr. 2.5 was subjected to silica gel H CC with a dichloromethane–methanol gradient (50:1 to 0:1), resulting in eight subfractions (Fr. 2.5.1 to Fr. 2.5.8). Fr. 2.5.5 was purified by semipreparative HPLC using ACN/H_2_O (1:3) as the elution solvent to generate compounds **1** (3.1 mg), **2** (2.7 mg), **3** (2.1 mg), **4** (1.7 mg), **5** (1.7 mg), **6** (4.4 mg), and **7** (2.8 mg).

Compound **1**. Pale yellow powder; [α${]}_{D}^{25}$ −1.13 (c 0.04, MeOH); UV (MeOH) *λ*_max_ (logє) 209.5 nm (3.72), 223.5 nm (3.59), and 280.5 nm (3.17); IR (KBr) *v*_max_ 3376, 3070, 2919, 1610, 1511, 1220, 1074, and 1022 cm^−1^; CD (MeOH) 204.1 nm (−1.43), 210.3 nm (−7.11), 215.7 nm (−3.17), 220.5 nm (−4.00), 227.3 nm (−1.90), 247.4 nm (+0.26), and 278.8 nm (−1.45); ^1^H-NMR (CD_3_OD, 600 MHz) and ^13^C-NMR (CD_3_OD, 150 MHz) data in Table S1; HRESIMS (*m*/*z*): 415.1363 [M + Na]^+^ (calcd. for C_20_H_24_O_8_Na, 415.1363).

Compound **2**. Pale yellow powder; [α${]}_{D}^{25}$ +2.59 (c 0.04, MeOH); UV (MeOH) *λ*_max_ (logє) 218.5 nm (3.61) and 279.5 nm (2.97); IR (KBr) *v*_max_ 3384, 2925, 1612, 1509, 1226, 1076, and 819 cm^−1^; CD (MeOH) 210.0 nm (+9.08), 223.0 nm (−6.75), and 271.2 nm (−2.33); ^1^H-NMR (CD_3_OD, 600 MHz) and ^13^C-NMR (CD_3_OD, 150 MHz) data in Table S1; HRESIMS (*m*/*z*): 399.1409 [M + Na]^+^ (calcd. for C_20_H_24_O_7_Na, 399.1414).

Compound **13**. White amorphous powder; UV (MeOH) *λ*_max_ (logє) 209.5 nm (4.21), 229.5 nm (4.14), and 251.5 nm (4.05); IR (KBr) *v*_max_ 3455, 3268, 2784, 1691, 1590, 1511, 1228, and 767 cm^−1^; ^1^H-NMR (CD_3_OD, 600 MHz) and ^13^C-NMR (CD_3_OD, 150 MHz) data in Table S2; HRESIMS (*m*/*z*): 243.0871 [M + H]^+^ (calcd. for C_12_H_11_N_4_O_2_, 243.0877).

Compound **17**. Colorless transparent gum (MeOH); [α${]}_{D}^{25}$ −0.26 (c 0.31, MeOH); UV (MeOH) *λ*_max_ (logє) 203.5 nm (3.18), 209.1 nm (3.16), and 261.2 nm (2.68); IR (KBr) *v*_max_ 3380, 2954, 2871, 1733, 1438, 1394, 1236, 1074, 1031, and 644 cm^−1^; CD (MeOH) 211.0 nm (+72.57); ^1^H-NMR (CD_3_OD, 600 MHz) and ^13^C-NMR (CD_3_OD, 150 MHz) data are shown in Table S3; HRESIMS (*m*/*z*): 389.1423 [M+Na]^+^ (calcd. for C_15_H_26_O_10_Na, 389.1418).

### 3.4. Acid Hydrolysis

Compound **1** (1.5 mg) was heated at 80 °C for 4 h in 3 mL of 10% HCl–dioxane (1:1). After dioxane was removed, the solution was extracted with ethyl acetate (3 mL × 3) to obtain aglycones and sugars. The aqueous layer was neutralized with NaHCO_3_ and concentrated [31]. The residual sugar components in the water layer after acid hydrolysis were analyzed by TLC and compared with those of standard sugars. The solvent system was CHCl_3_–MeOH–H_2_O (8:5:1), and 95% EtOH–H_2_SO_4_–anisaldehyde (9:0.5:0.5, *v*/*v*) was sprayed to yield spots by heating at 120 °C for 10 min. The same analysis was carried out on compounds **2** and **17**.

### 3.5. Computer Simulations

The computational chemistry workflow involved the use of Gaussian 09 for both density functional theory (DFT) and time-dependent density functional theory (TD-DFT) calculations. GaussView was used for structure generation and analysis, and theoretical calculations were conducted on the ECD or NMR data of the compounds. Fitting was performed via Origin 2021 and SpecDis 1.71 (σ = 0.30 eV). With the help of the SPARTAN 16 program package, the initial conformation of compound **17** was analyzed via the Monte Carlo search algorithm through the MMFF94 molecular mechanics force field, and the low-energy conformation accounting for more than 2% of the equilibrium population was applied to the next calculation. The minimum energy conformation of the force field obtained was subsequently optimized by performing DFT calculations at the B3LYP/6-31G (d) energy level in the Gaussian 09 software package. The NMR computations were performed with DFT calculations at the mPW1PW91/6-311G (2d, p) level in MeOH via the polarizable continuum model (PCM) solvent model. Additionally, structural confirmation was enhanced via methods such as DP4+. For the ECD calculations, by using PCM solvent model, time-dependent density functional theory (TDDFT) calculations were carried out at the B3LYP/6-311G (d, p) level in MeOH to perform theoretical ECD calculations on these main conformations. The energy, oscillator strength, and rotational strength of each conformation were calculated via the Gaussian 09 software package. According to the Boltzmann weighted overall contribution of SpecDisv1.71, the final ECD spectra of the individual conformers were summed.

### 3.6. Neuroprotective Effect Evaluation

The PC12 cells were cultured in complete RPMI 1640 medium supplemented with 10% fetal bovine serum and 1% penicillin-streptomycin. The cells were inoculated at a density of 5 × 10^4^ cells/mL into a 96-well plate in the following groups: control group, OGD/R group, and treatment group (OGD/R + tested fractions or compounds). After the cells had adhered to the well, the original culture medium was removed, and the wells were washed twice with PBS. The control group was replenished with the original culture medium and maintained under the original conditions. Moreover, the OGD/R and treatment groups were supplied glucose-free DMEM and subjected to low-oxygen treatment for 6 h in a three-gas incubator containing 1% O_2_, 94% N_2_, and 5% CO_2_. Following the deprivation period, the DMEM glucose-free culture medium was replaced with complete 1640 medium (OGD/R group) or medium containing various concentrations of fractions or compounds, and the cells were maintained under the original conditions to stop glucose and oxygen deprivation. After 24 h, the intervention was terminated, and 10 μL of CCK-8 working solution was added to each well. The plate was then incubated for 90 min. Finally, the absorbance value (OD value) was measured via an enzyme-linked immunosorbent assay (ELISA) at a wavelength of 450 nm. Edaravone was used as the positive control.

### 3.7. RNA Extraction, Library Preparation and Sequencing

Total RNA was extracted via a TRIzol reagent kit (Invitrogen, Carlsbad, CA, USA) according to the manufacturer’s protocol. RNA quality was assessed on an Agilent 2100 Bioanalyzer (Agilent Technologies, Palo Alto, CA, USA) and checked via RNase-free agarose gel electrophoresis. RNA purification, reverse transcription, library construction, and sequencing were performed at Shanghai Majorbio Biopharm Biotechnology Co., Ltd. (Shanghai, China) according to the manufacturer’s instructions (Illumina, San Diego, CA, USA). The raw paired-end reads resulting from sequencing were aligned to a reference rat genome (mRatBN7.2) via the rnaseq pipeline from nf-core.

### 3.8. Differential Expression Analysis

Differential gene expression analysis across various groups was performed via the R package DESeq2 (version: 1.34.0) [32], and genes with *p*-adj < 0.05 and |log_2_FC| ≥ 0.26 were identified as differentially expressed genes. Gene functional enrichment analysis was conducted via hypergeometric distribution tests using enrichGO and enrichKEGG in the R package clusterProfiler (version: 4.2.2) [33]. Enriched pathways with a significance threshold of *p* < 0.05 were retained.

### 3.9. Soft Clustering Analysis

The fuzzy c-means (FCM) clustering algorithm in the R package Mfuzz (version: 2.54.0) was used to perform soft-clustering analysis to identify various expression patterns of genes in time series experimental designs [34]. In this analysis, parameters c (number of clusters) and m (fuzzification parameter) were employed. The value of parameter c was determined by assessing the sum of the squared errors generated as the number of clusters increases. Additionally, the mestimate function within the Mfuzz software (version: 2.62.0) package was utilized to obtain a value for parameter m.

### 3.10. qPCR Assay

The qPCR primers were designed via Primer Premier 5.0 software on the basis of the gene sequence published in the GenBank database. The *GAPDH* gene was used as an internal control. All primers were synthesized by Sangon Biotechnology (Shanghai, China), and the corresponding sequences are shown in Table S4. qPCR analysis was performed via a real-time fluorescence quantification kit (Vazyme Biotech Co., Ltd. Nanjing, China). All qPCR assays were conducted in a 20 µL mixture composed of 2 µL of cDNA, 0.4 µL of each primer (10 µmol/L), 10 µL of 2×AceQ Universal SYBR qPCR Master Mix, and 7.2 µL of ddH_2_O. The thermocycler settings were as follows: 95 °C for 5 min; 40 cycles at 95 °C for 5 s and 60 °C for 30 s. Melting curves were then used to confirm the specificity of the amplified products. Three independent experimental replicates were conducted for all analyses. The results of relative quantification were analyzed and processed via the 2^−ΔΔCt^ method.

### 3.11. Statistical Analysis

All the data are presented as the means ± SDs for each group and were evaluated via one-way analysis of variance (ANOVA). All the results of the cell experiments were statistically analyzed via SPSS Version 25.0 for Windows (IBM SPSS Inc., Chicago, IL, USA). *p* < 0.05 was considered statistically significant. All the results of the RNA-Seq experiments were statistically analyzed in the R environment (version: 4.1.3), and the data were visualized via the R package ggplot2 (version: 3.3.5).

## 4. Conclusions

In the present study, we revealed the in vitro antistroke effect of *G. conopsea* for the first time, which offers a new angle of view for its pharmacological action study. Then, a bioactivity-guided separation strategy was performed to identify the antistroke constituents of *G. conopsea*. This process led to the isolation of 4 undescribed compounds, along with 13 known compounds, among which 8 compounds exhibited protective effects on OGD/R-injured PC12 cells at the tested concentrations. More importantly, compound **17** is a promising candidate for the development of novel antistroke drugs and deserves further study.

## Figures and Tables

**Figure 2:**
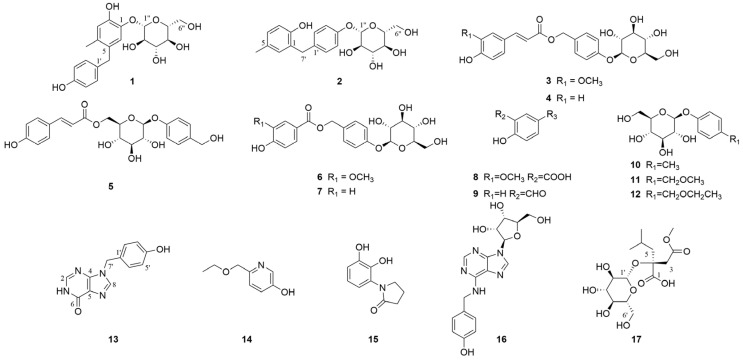
Structures of compounds **1**–**17** isolated from *G. conopsea*.

**Figure 3:**
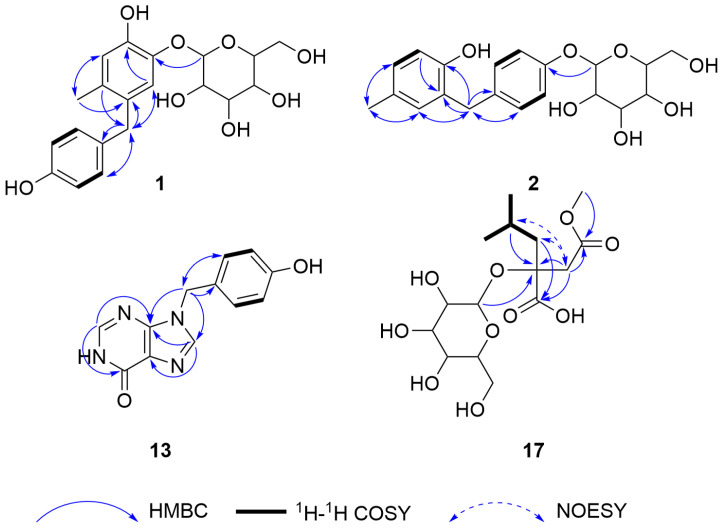
Key HMBC and ^1^H-^1^H COSY correlations of compounds **1**–**2**, **13**, and **17**.

## Data Availability

The original contributions presented in the study are included in the article/Supplemental Materials. Further inquiries can be directed to the corresponding author.

## Supplementary Materials

The supporting information can be downloaded at https://www.mdpi.com/article/10.3390/molecules29184389/s1.
