# Supplementary material for: Bioactivity-Guided Isolation of Antistroke Compounds from Gymnadenia conopsea (L.) R. Br
Source: Molecules. 2024 Sep 15;29(18):4389. doi: 10.3390/molecules29184389 (PMC11434265; doi:10.3390/molecules29184389)
Supplement: Supplementary file 1 [file molecules-29-04389-s001.zip › molecules-3148992-supplementary.pdf]

Supplementary Materials

Bioactivity-Guided Isolation of Antistroke Compounds from Gymnadenia conopsea (L.) R. Br.

Juan Qin [1,2], Shiyi Xue [1,2], Chao Xu [3], Jian Jin [3], Jianbin Wang [1,2], Hailian Yuan [1,2] and Liang Liu [1,2,*]

1 Institute of Translational Medicine, Medical College, Yangzhou University, Yangzhou 225009, China

2 Jiangsu Key Laboratory of Integrated Traditional Chinese and Western Medicine for Prevention and Treatment of Senile Diseases, Yangzhou University, Yangzhou 225009, China

3 College of Animal Science and Technology, Yangzhou University, Yangzhou 225009, China

* Corresponding: enjoyyz@163.com

Table of Contents

Figure S1. HRESIMS spectrum of **1**. --4
Figure S2. UV spectrum of **1**. --4
Figure S3. IR spectrum of **1**. --5
Figure S4. CD spectrum of **1**. --5
Figure S5. ^{1}H NMR spectrum of **1**. -- 6
Figure S6. ^{13}C NMR spectrum of **1**. --6
Figure S7. HSQC spectrum of **1**. -- 7
Figure S8. ^{1}H-^{1}H COSY spectrum of **1**. -- 7
Figure S9. HMBC spectrum of **1**. -- 8
Figure S10. NOESY spectrum of **1**. --8
Figure S11. HRESIMS spectrum of **2**. --9
Figure S12. UV spectrum of **2**. --9
Figure S13. IR spectrum of **2**. --10
Figure S14. CD spectrum of **2**. --10
Figure S15. ^{1}H NMR spectrum of **2**. -- 11
Figure S16. ^{13}C NMR spectrum of **2**. --11
Figure S17. HSQC spectrum of **2**. -- 12
Figure S18. ^{1}H-^{1}H COSY spectrum of **2**. -- 12
Figure S19. HMBC spectrum of **2**. -- 13
Figure S20. NOESY spectrum of **2**. --13
Figure S21. HRESIMS spectrum of **13**. --14
Figure S22. UV spectrum of **13**. -- 14
Figure S23. IR spectrum of **13**. --15
Figure S24. ^{1}H NMR spectrum of **13**. -- 15
Figure S25. ^{13}C NMR spectrum of **13**. --16
Figure S26. HSQC spectrum of **13**. -- 16
Figure S27. ^{1}H-^{1}H COSY spectrum of **13**. -- 17
Figure S28. HMBC spectrum of **13**. -- 17
Figure S29. NOESY spectrum of **13**. --18
Figure S30 HRESIMS spectrum of **17**. -- 18
Figure S31 UV spectrum of **17**. -- 19
Figure S32 IR spectrum of **17**. -- 19
Figure S33 CD spectrum of **17**. -- 20
Figure S34 ^{1}H NMR spectrum of **17**. -- 20
Figure S35 ^{13}C NMR spectrum of **17**. -- 21
Figure S36 HSQC spectrum of **17**. -- 21
Figure S37 ^{1}H-^{1}H COSY spectrum of **17**. -- 22
Figure S38 HMBC spectrum of **17**. -- 22
Figure S39 NOESY spectrum of **17**. -- 23
Figure S40. HRESIMS spectrum of **3**. -- 23
Figure S41. ^{1}H NMR spectrum of **3**. -- 24

Figure S42. ^{13}C NMR spectrum of **3**. -- 24
Figure S43. HRESIMS spectrum of **4**. -- 25
Figure S44. ^{1}H NMR spectrum of **4**. -- 25
Figure S45. ^{13}C NMR spectrum of **4**. -- 26
Figure S46. HRESIMS spectrum of **5**. -- 26
Figure S47. ^{1}H NMR spectrum of **5**. -- 27
Figure S48. ^{13}C NMR spectrum of **5**. -- 27
Figure S49. HRESIMS spectrum of **6**. -- 28
Figure S50. ^{1}H NMR spectrum of **6**. -- 28
Figure S51. ^{13}C NMR spectrum of **6**. -- 29
Figure S52. HRESIMS spectrum of **7**. -- 29
Figure S53. ^{1}H NMR spectrum of **7**. -- 30
Figure S54. ^{13}C NMR spectrum of **7**. -- 30
Figure S55. HRESIMS spectrum of **8**. -- 31
Figure S56. ^{1}H NMR spectrum of **8**. -- 31
Figure S57. ^{13}C NMR spectrum of **8**. -- 32
Figure S58. HRESIMS spectrum of **9**. -- 32
Figure S59. ^{1}H NMR spectrum of **9**. -- 33
Figure S60. ^{13}C NMR spectrum of **9**. -- 33
Figure S61. HRESIMS spectrum of **10**. -- 34
Figure S62. ^{1}H NMR spectrum of **10**. -- 34
Figure S63. ^{13}C NMR spectrum of **10**. -- 35
Figure S64. HRESIMS spectrum of **11**. -- 35
Figure S65. ^{1}H NMR spectrum of **11**. -- 36
Figure S66. ^{13}C NMR spectrum of **11**. -- 36
Figure S67. HRESIMS spectrum of **12**. -- 37
Figure S68. ^{1}H NMR spectrum of **12**. -- 37
Figure S69. ^{13}C NMR spectrum of **12**. -- 38
Figure S70. HRESIMS spectrum of **14**. -- 38
Figure S71. ^{1}H NMR spectrum of **14**. -- 39
Figure S72. ^{13}C NMR spectrum of **14**. -- 39
Figure S73. HRESIMS spectrum of **15**. -- 40
Figure S74. ^{1}H NMR spectrum of **15**. -- 40
Figure S75.^{13}C NMR spectrum of **15**. -- 41
Figure S76. HRESIMS spectrum of **16**. -- 41
Figure S77. ^{1}H NMR spectrum of **16**. -- 42
Figure S78. ^{13}C NMR spectrum of **16**. -- 42

Table S1 ^{1}H and ^{13}C-NMR date of compounds **1** and **2** recorded in CD_3OD (δ in ppm, J in Hz) -- 43
Table S2 ^{1}H and ^{13}C-NMR date of compound **13** recorded in CD_3OD (δ in ppm, J in Hz) --- 44
Table S3 ^{1}H and ^{13}C-NMR date of compounds **17** recorded in CD_3OD (δ in ppm, J in Hz) -- 45
Table S4 The primer sequence of genes for qPCR ------------------------------------ 46

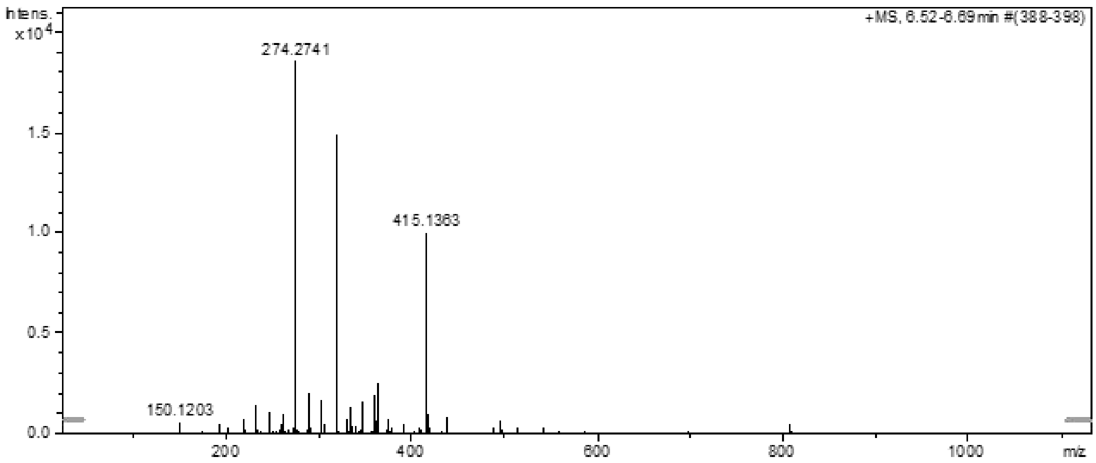

Meas. m/z	#	Formula	Score	m/z	err [mDa]	err [ppm]	mSigma	rdb	e^- Conf	N-Rule
415.1363	1	C 13 H 28 Na O 13	0.98	415.1422	5.9	14.2	38.9	-0.5	even	ok
	2	C 20 H 24 Na O 8	100	415.1363	0	0.1	73	8.5	even	ok
	3	C 22 H 23 O 8	14.57	415.1387	2.4	5.8	84.1	11.5	even	ok
	4	C 27 H 20 Na O 3	0.06	415.1305	-5.9	-14.1	111	17.5	even	ok
	5	C 29 H 19 O 3	0.86	415.1329	-3.4	-8.3	122.2	20.5	even	ok

Figure S1. HRESIMS spectrum of **1**.

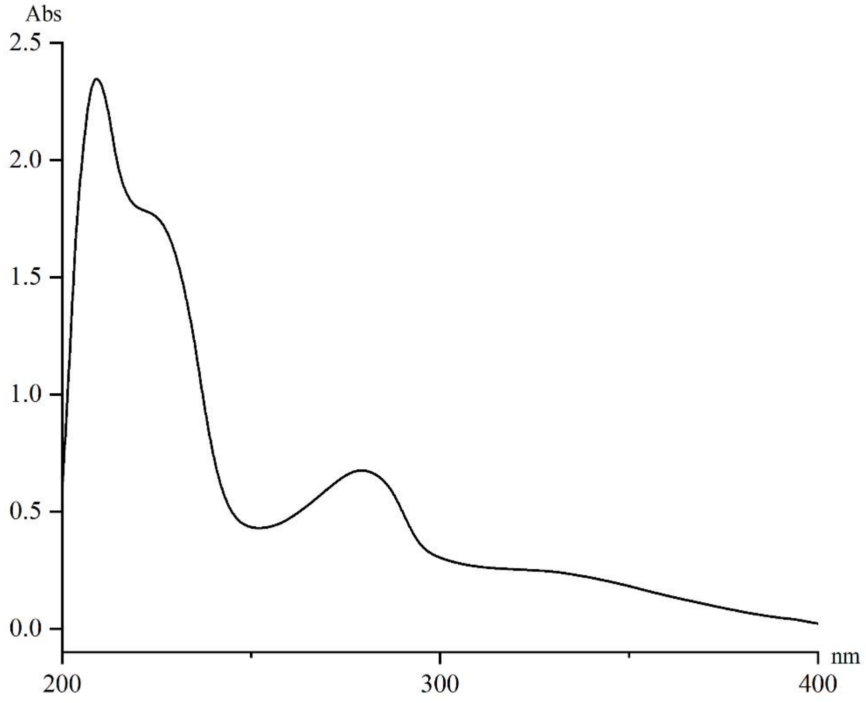

Figure S2. UV spectrum of **1**.

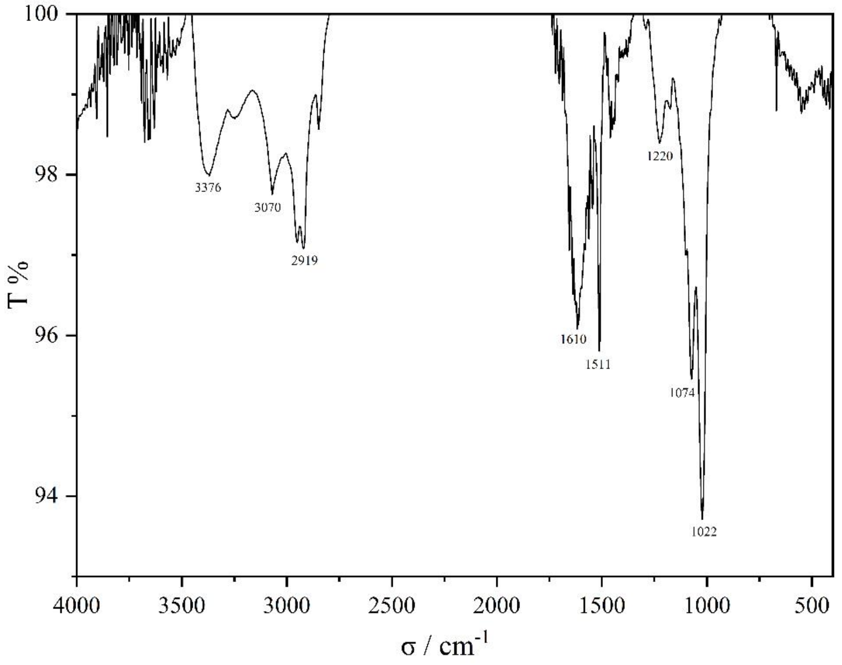

Figure S3. IR spectrum of **1**.

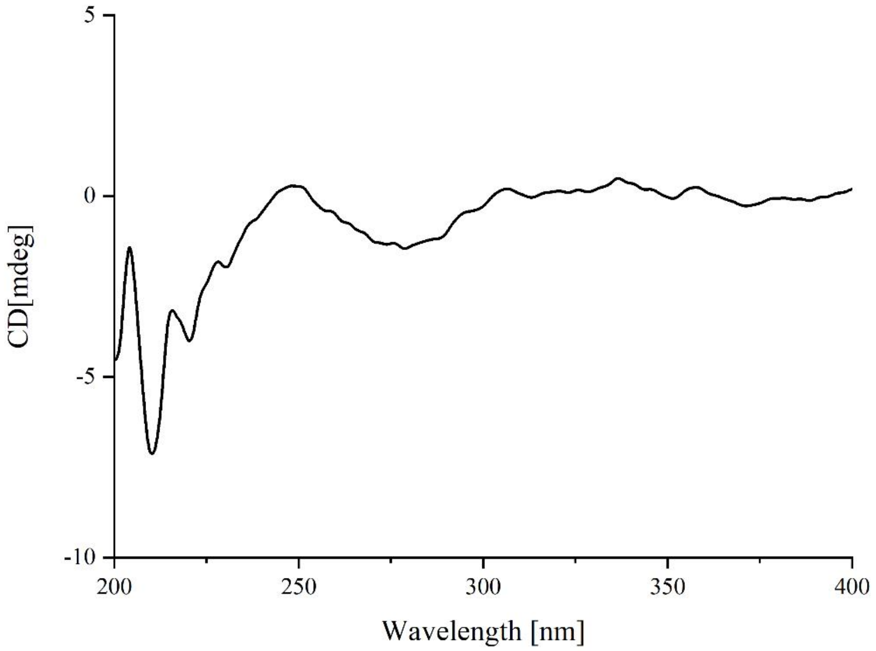

Figure S4. CD spectrum of **1**.

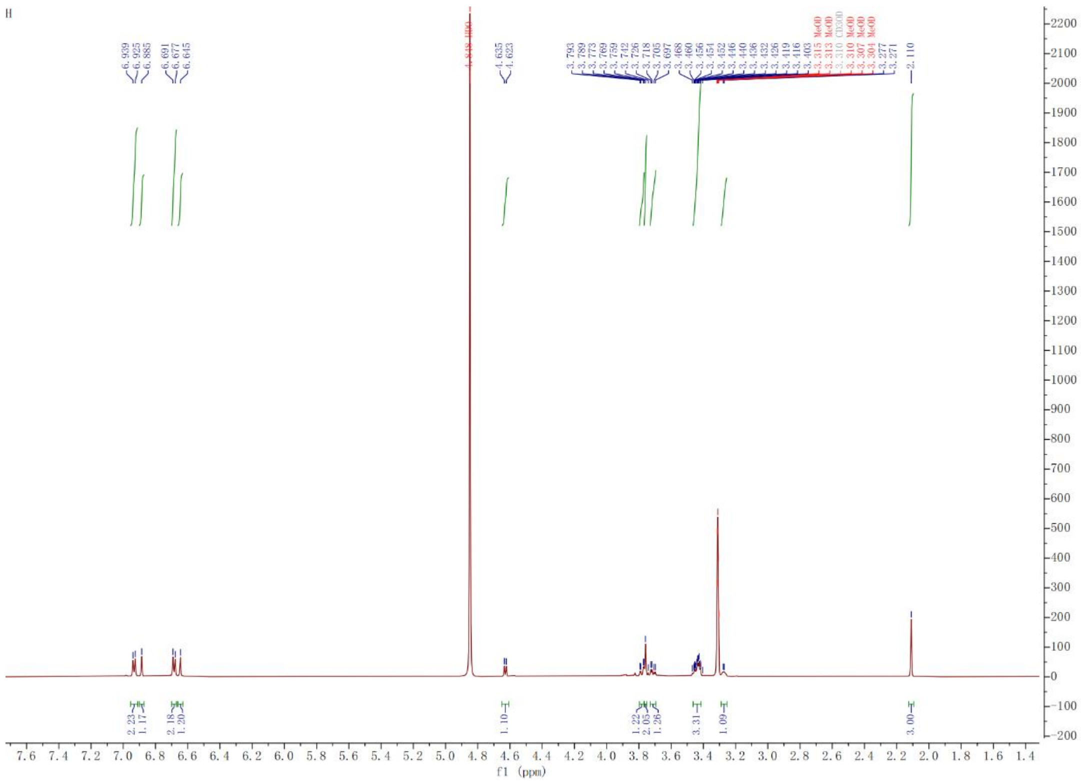

Figure S5. ^{1}H NMR spectrum of **1**.

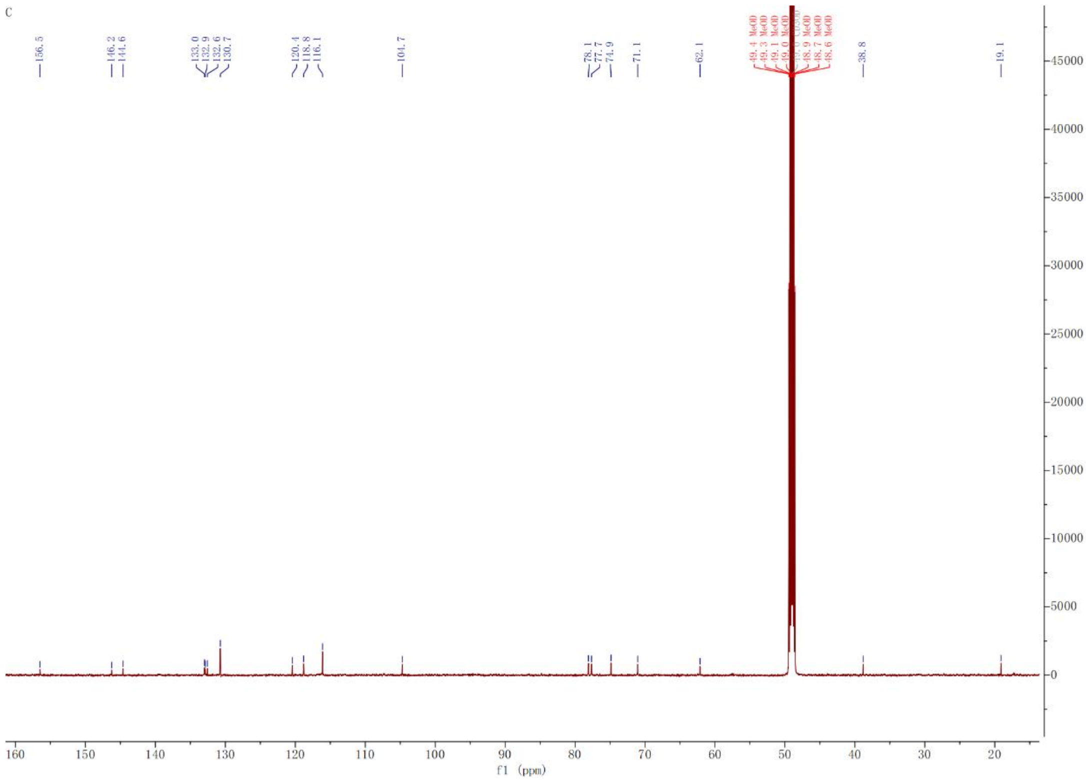

Figure S6. ^{13}C NMR spectrum of **1**.

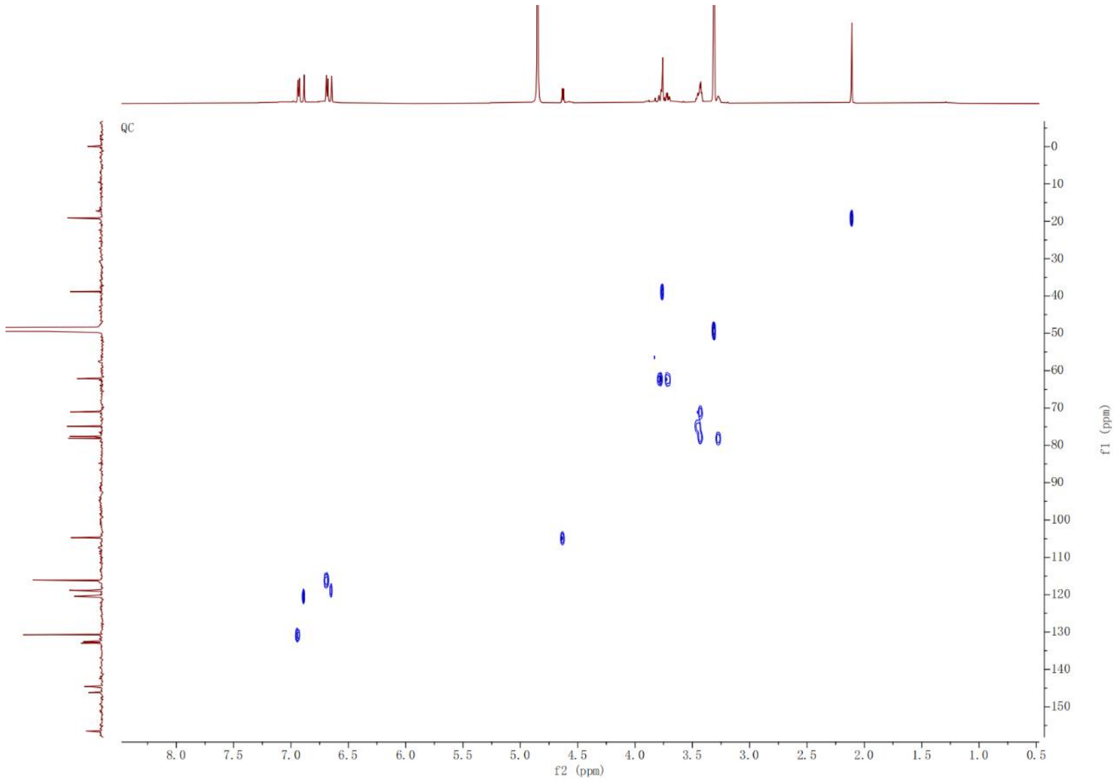

Figure S7. HSQC spectrum of **1**.

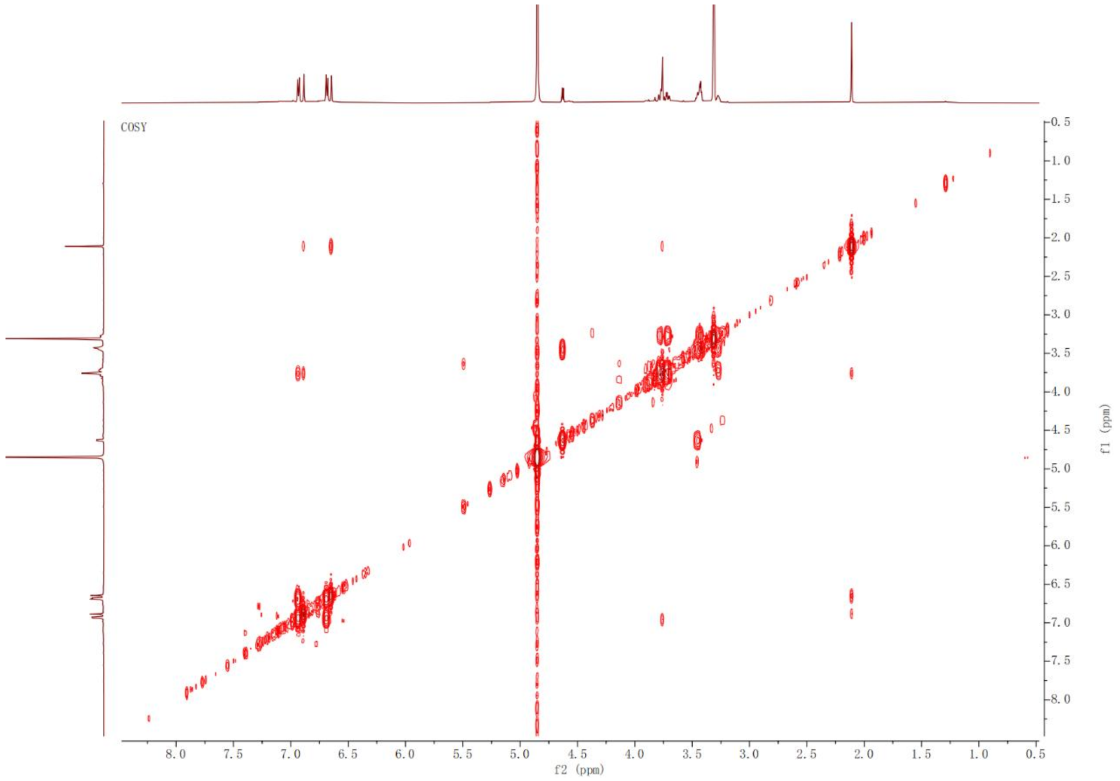

Figure S8. ^{1}H-^{1}H COSY spectrum of **1**.

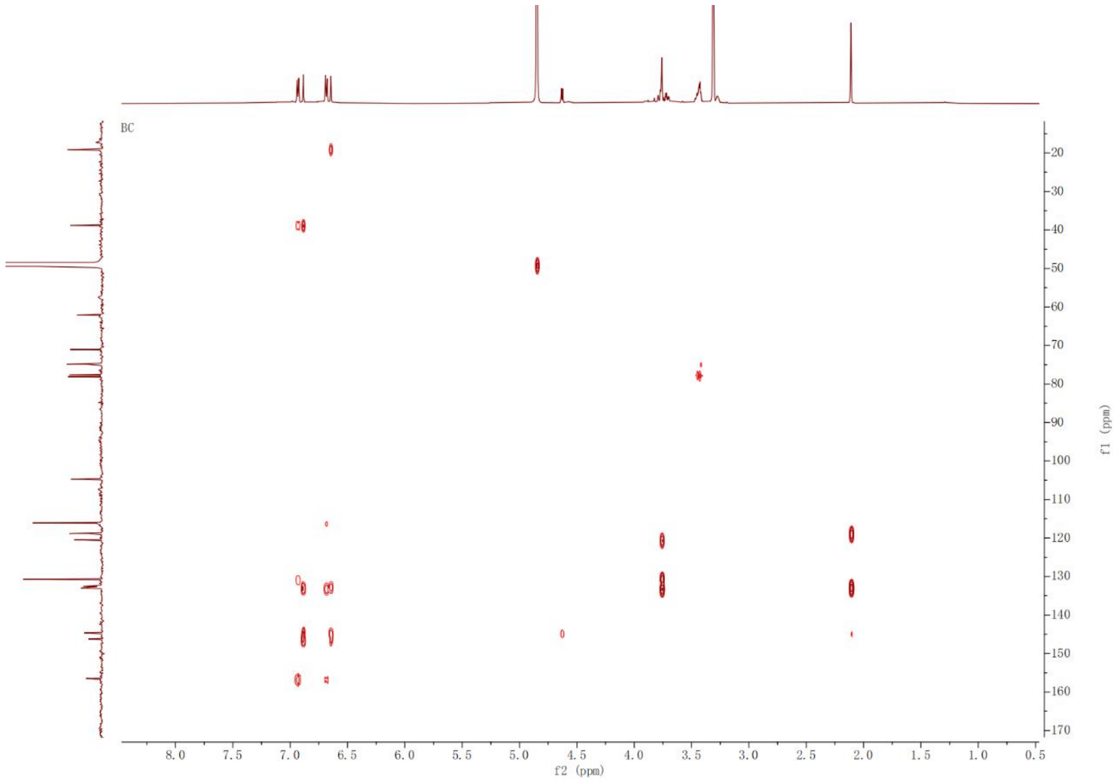

Figure S9. HMBC spectrum of **1**.

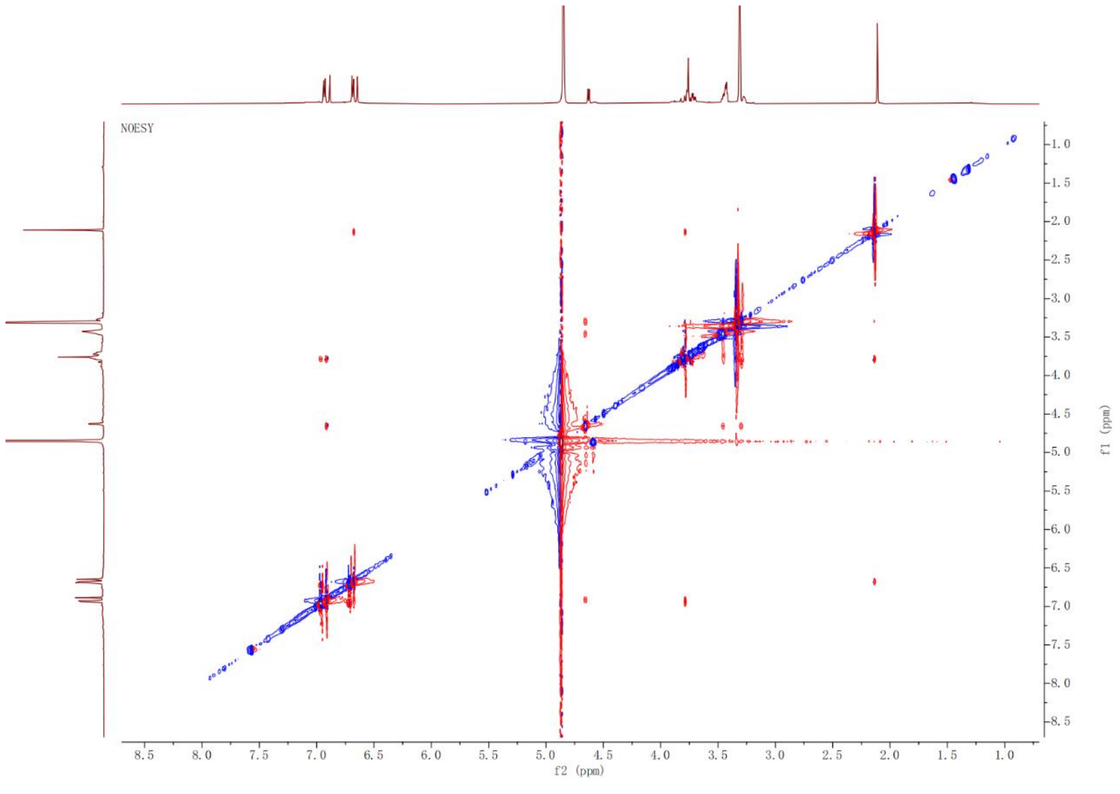

Figure S10. NOESY spectrum of **1**.

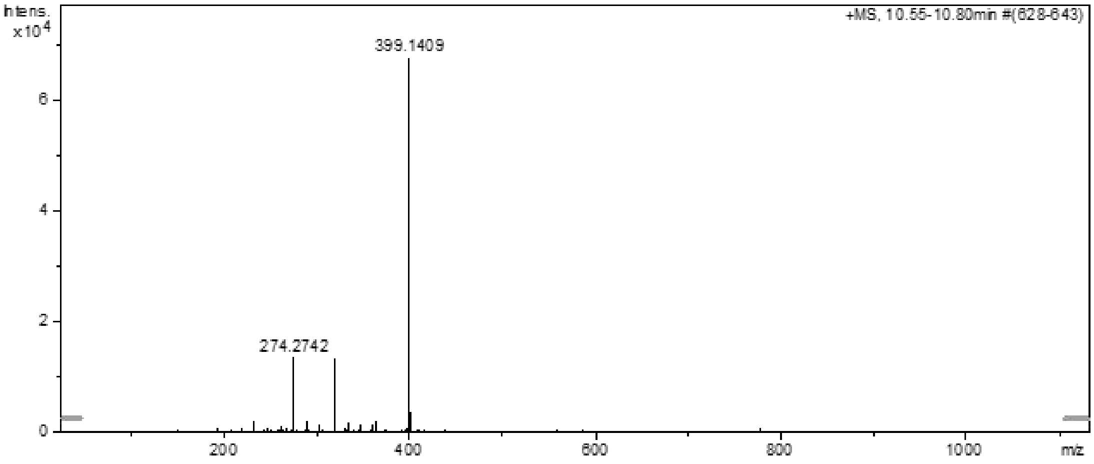

Meas. m/:	#	Formula	Score	m/z	err [mDa	err [ppm	mSigma	rdb	e¯ Conf	N-Rule
399.1409	1	C 13 H 28 Na O 12	0.84	399.1473	6.4	16	57.6	-0.5	even	ok
	2	C 20 H 24 Na O 7	100	399.1414	0.5	1.3	97.5	8.5	even	ok
	3	C 22 H 23 O 7	9.64	399.1438	2.9	7.3	110.3	11.5	even	ok
	4	C 27 H 20 Na O 2	0.27	399.1356	-5.3	-13.4	122.4	17.5	even	ok
	5	C 29 H 19 O 2	2.62	399.138	-2.9	-7.4	133.6	20.5	even	ok

Figure S11. HRESIMS spectrum of **2**.

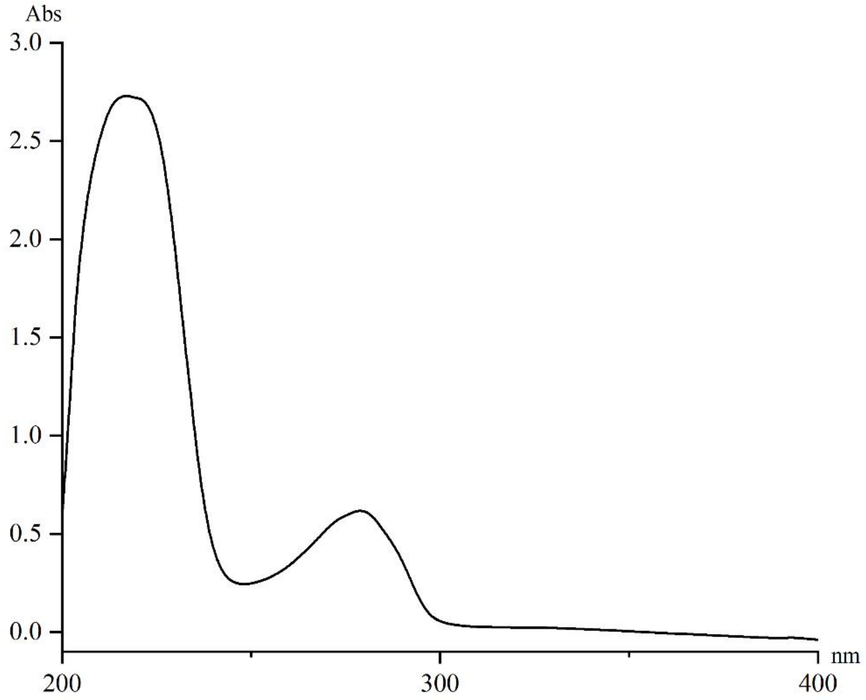

Figure S12. UV spectrum of **2**.

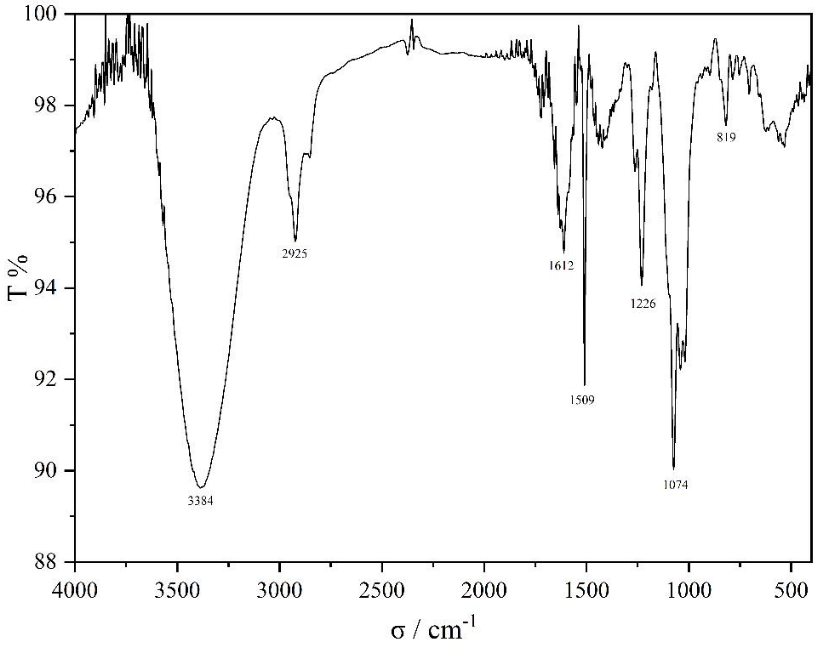

Figure S13. IR spectrum of **2**.

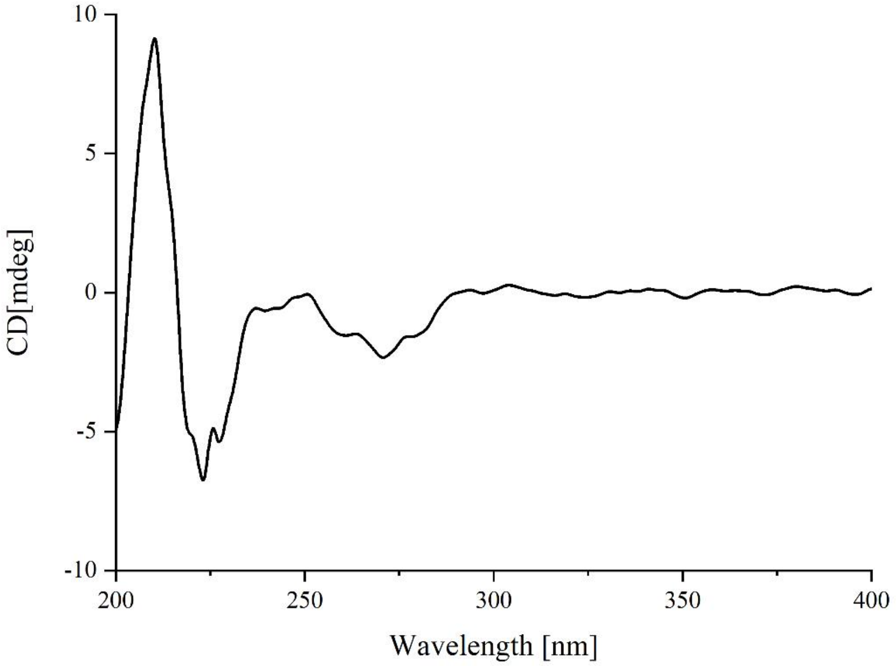

Figure S14. CD spectrum of **2**.

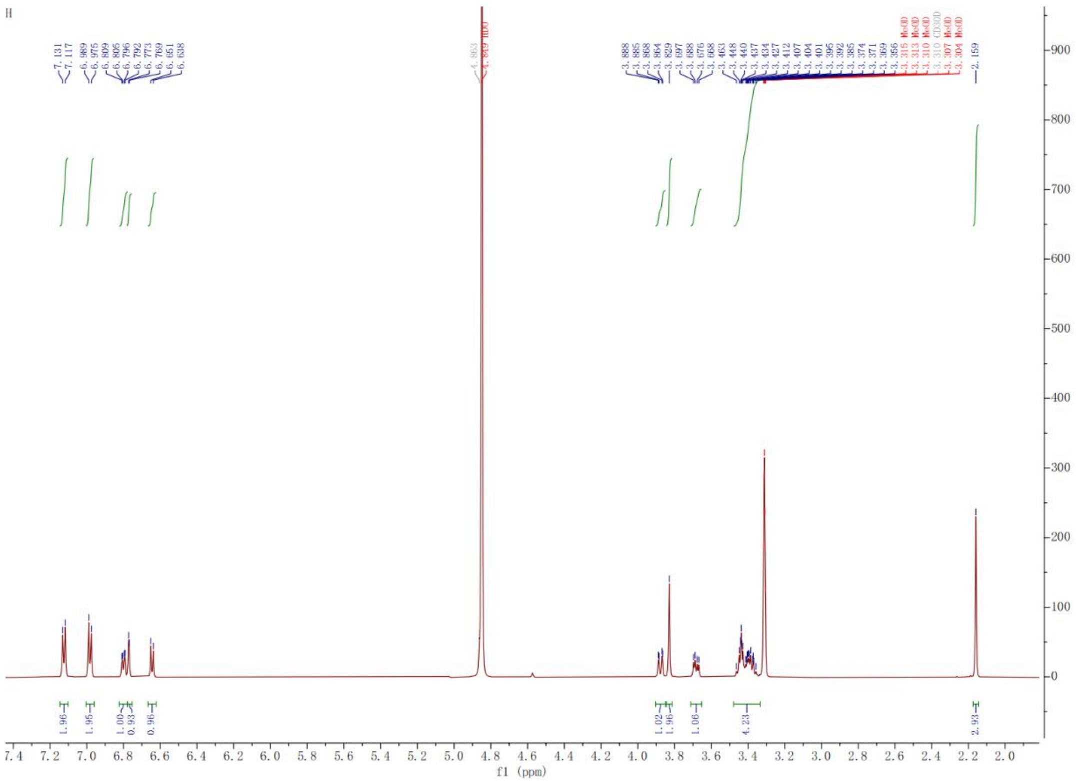

Figure S15. ^{1}H NMR spectrum of **2**.

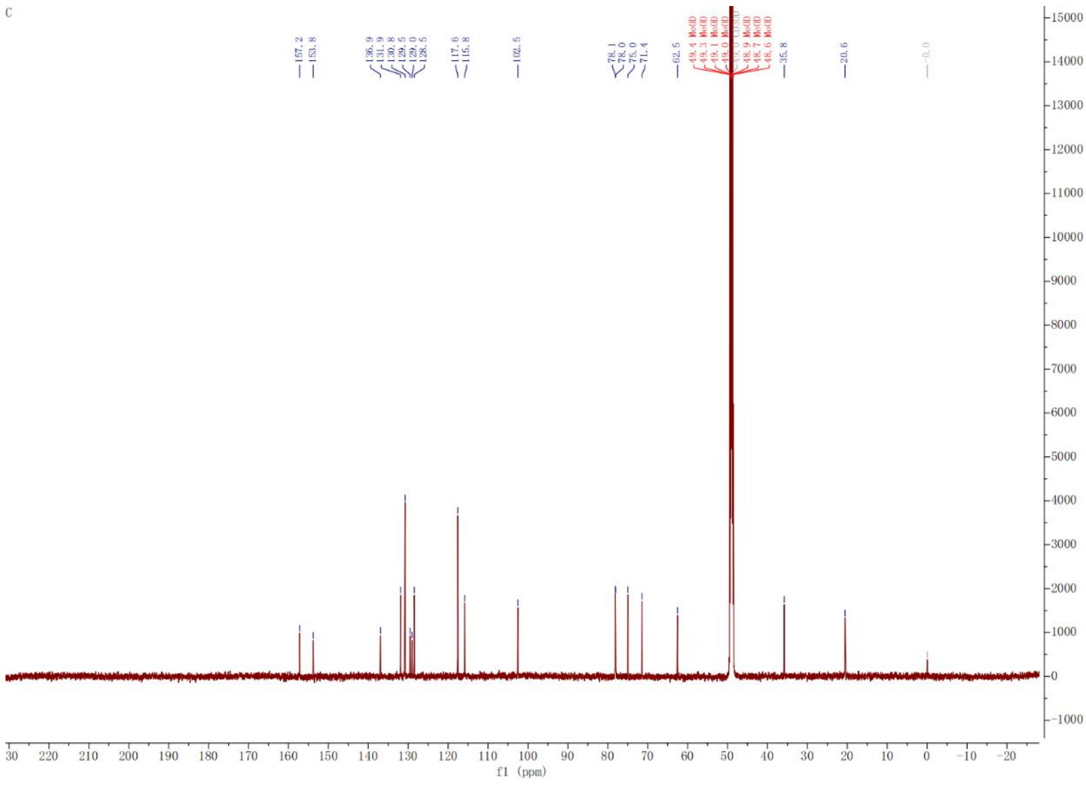

Figure S16. ^{13}C NMR spectrum of **2**.

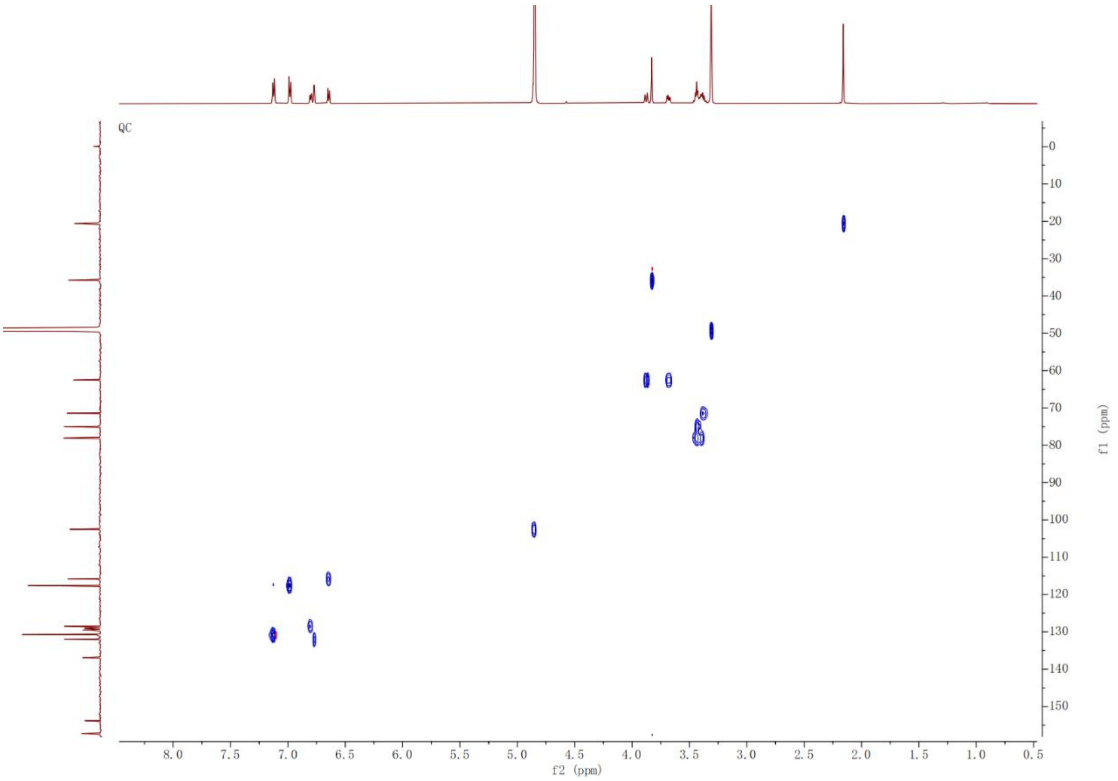

Figure S17. HSQC spectrum of **2**.

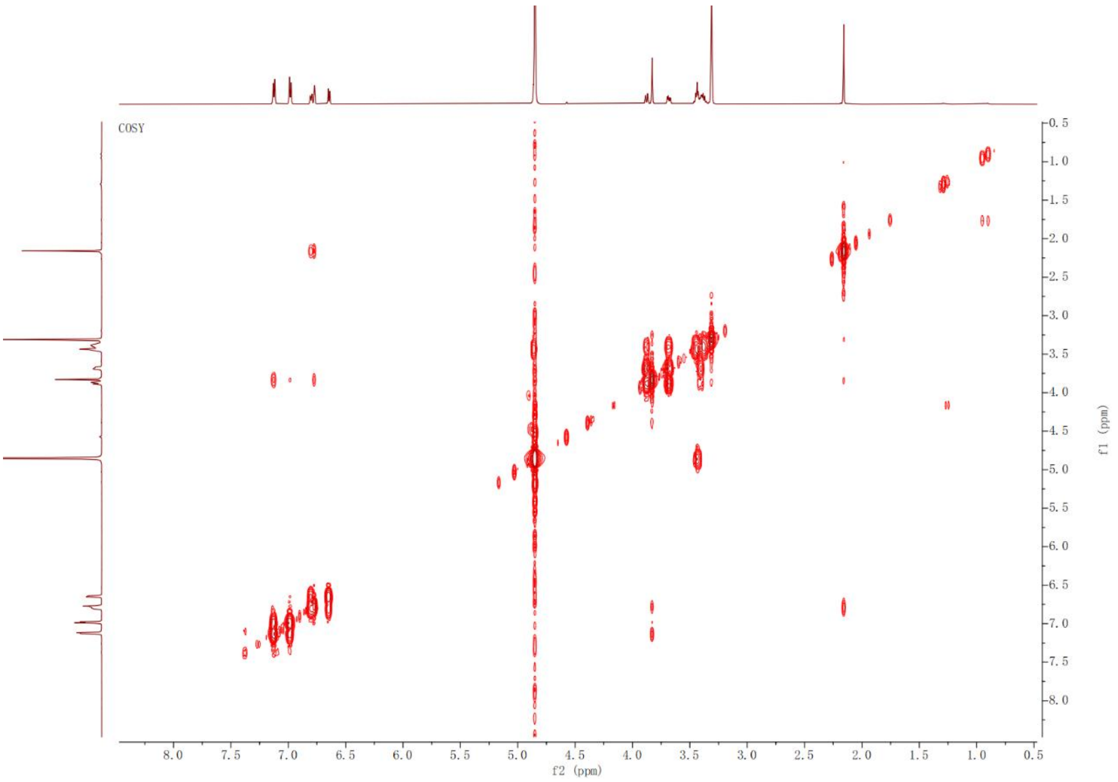

Figure S18. ^{1}H-^{1}H COSY spectrum of **2**.

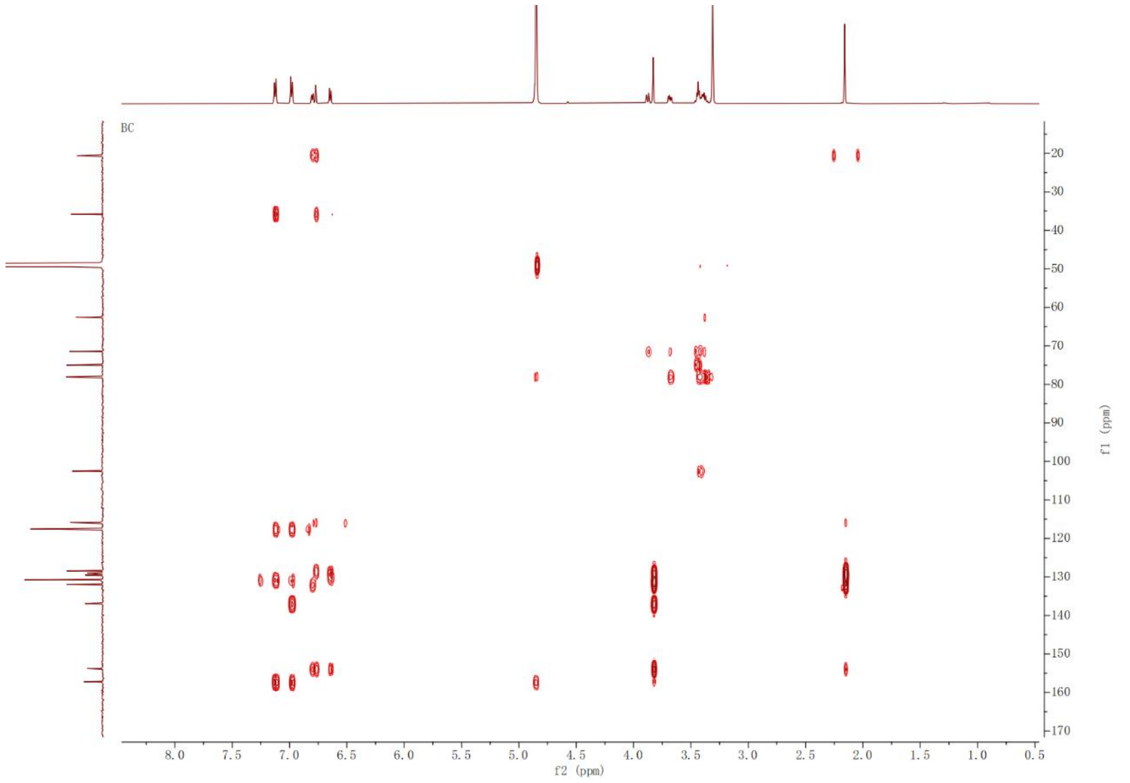

Figure S19. HMBC spectrum of **2**.

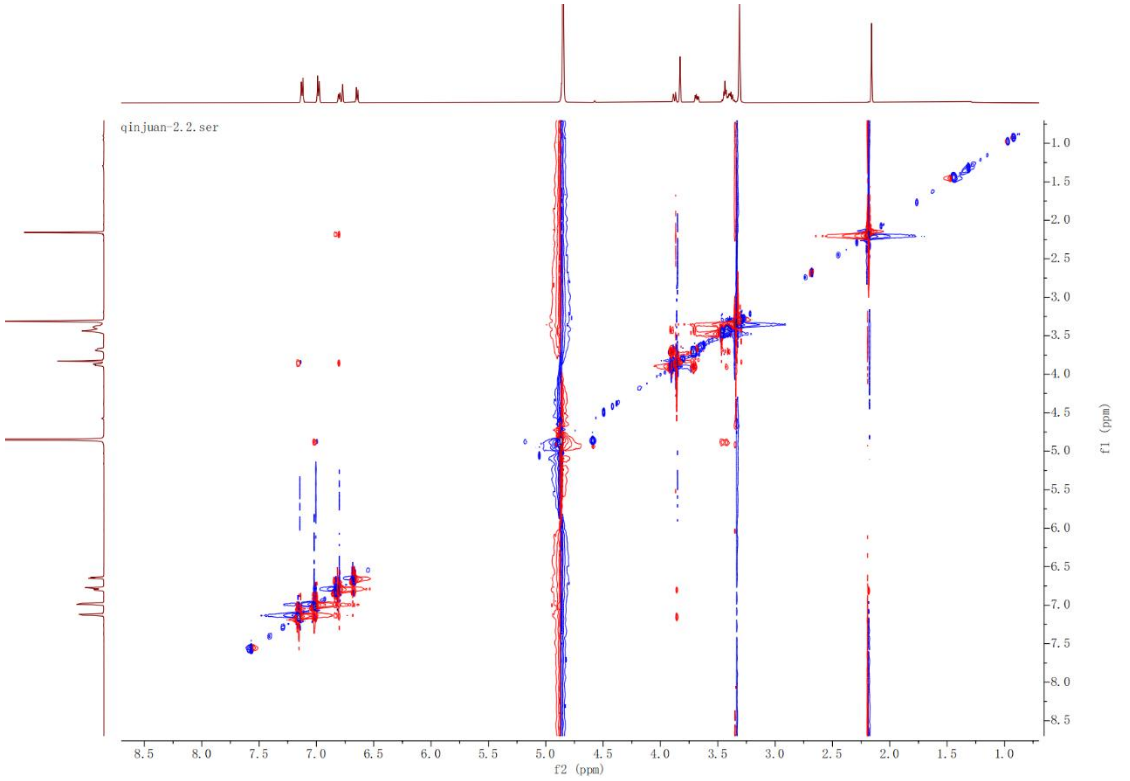

Figure S20. NOESY spectrum of **2**.

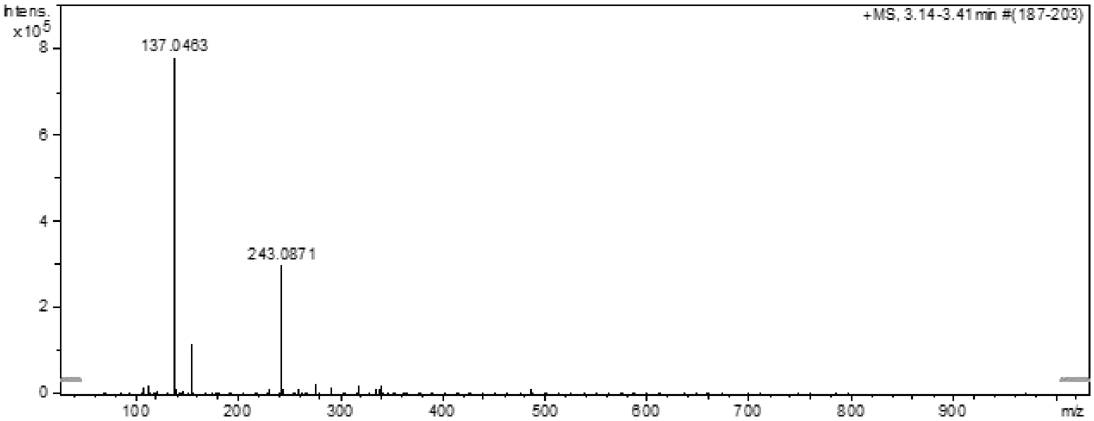

Meas. m/z	#	Formula	Score	m/z	err [mDa]	err [ppm]	mSigma	rdb	e⁻ Conf	N-Rule
243.0871	1	C 6 H 15 N 2 O 8	4.67	243.0823	-4.8	-19.8	23.6	0.5	even	ok
	2	C 7 H 11 N 6 O 4	17.17	243.0836	-3.5	-14.3	37	5.5	even	ok
	3	C 9 H 16 Na O 6	22.75	243.0839	-3.2	-13.1	37.3	1.5	even	ok
	4	C 6 H 8 N 10 Na	4.58	243.0826	-4.5	-18.7	39	7.5	even	ok
	5	C 11 H 15 O 6	100	243.0863	-0.8	-3.2	49.8	4.5	even	ok
	6	C 10 H 12 N 4 Na O 2	48.56	243.0852	-1.9	-7.6	51.4	6.5	even	ok
	7	C 8 H 7 N 10	39.17	243.085	-2.1	-8.8	51.5	10.5	even	ok
	8	C 12 H 11 N 4 O 2	70.67	243.0877	0.6	2.3	64	9.5	even	ok
	9	C 15 H 12 N 2 Na	14.63	243.0893	2.2	8.9	78.4	10.5	even	ok
	10	C 17 H 11 N 2	0.68	243.0917	4.6	18.8	91	13.5	even	ok

Figure S21. HRESIMS spectrum of **13**.

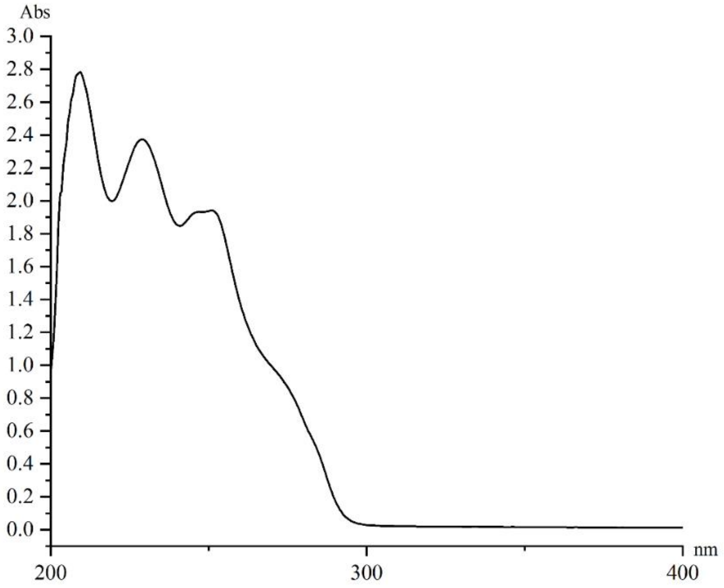

Figure S22. UV spectrum of **13**.

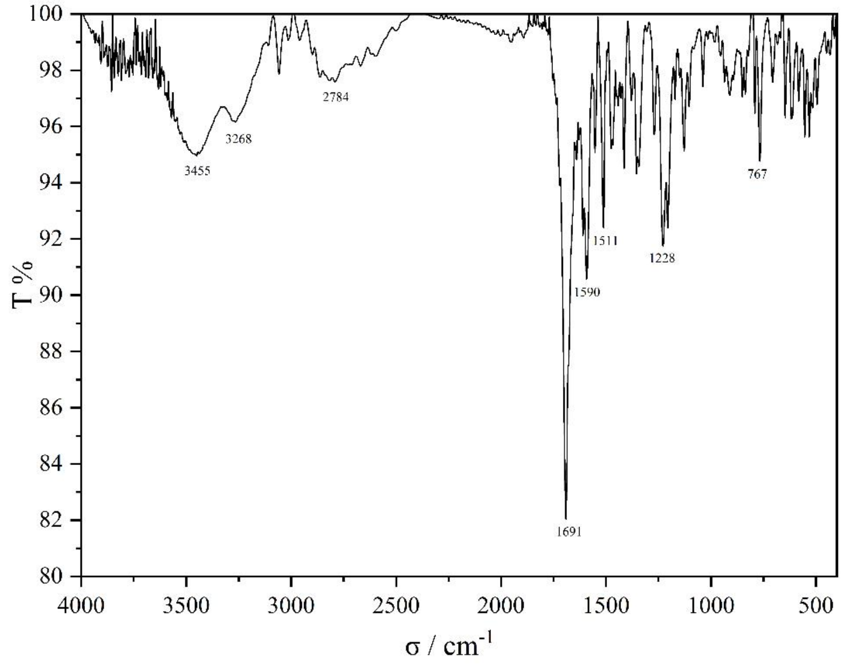

Figure S23. IR spectrum of **13**.

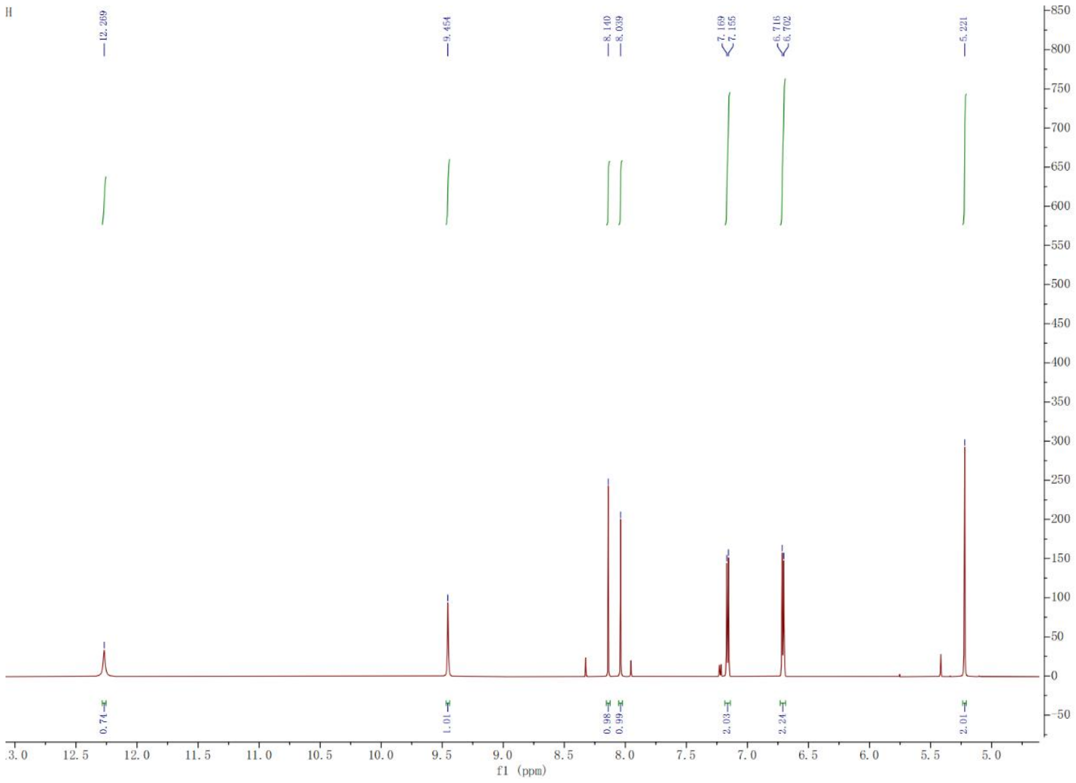

Figure S24. ^{1}H NMR spectrum of **13**.

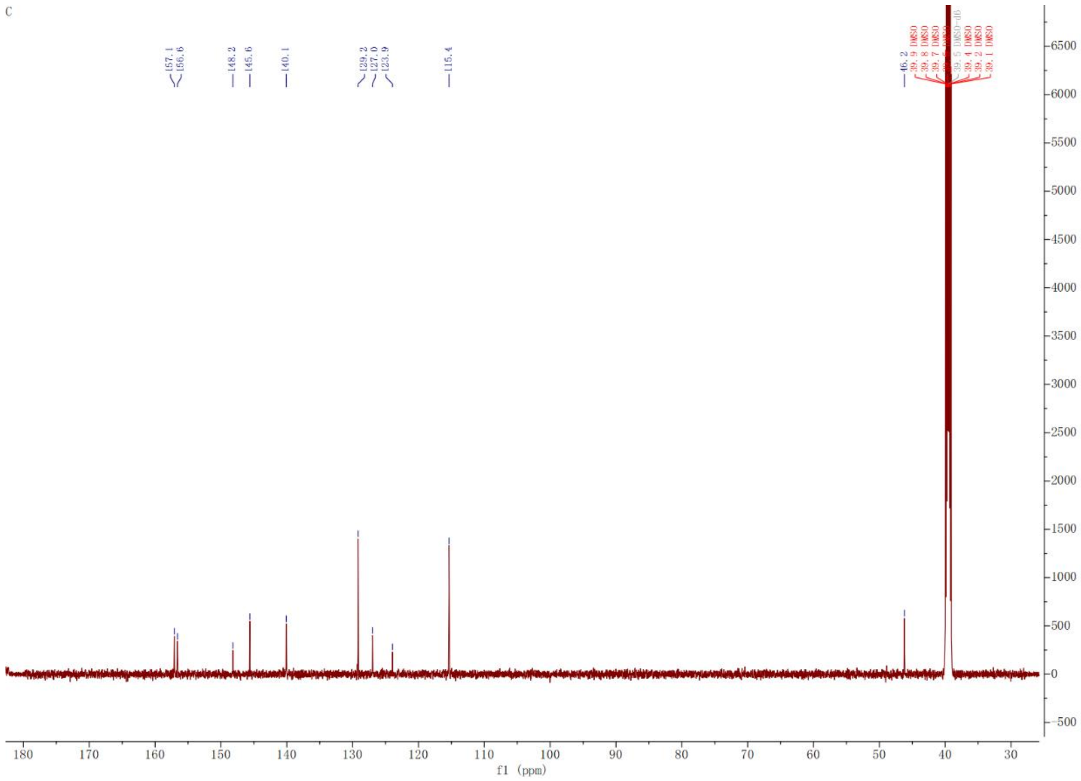

Figure S25. ^{13}C NMR spectrum of **13**.

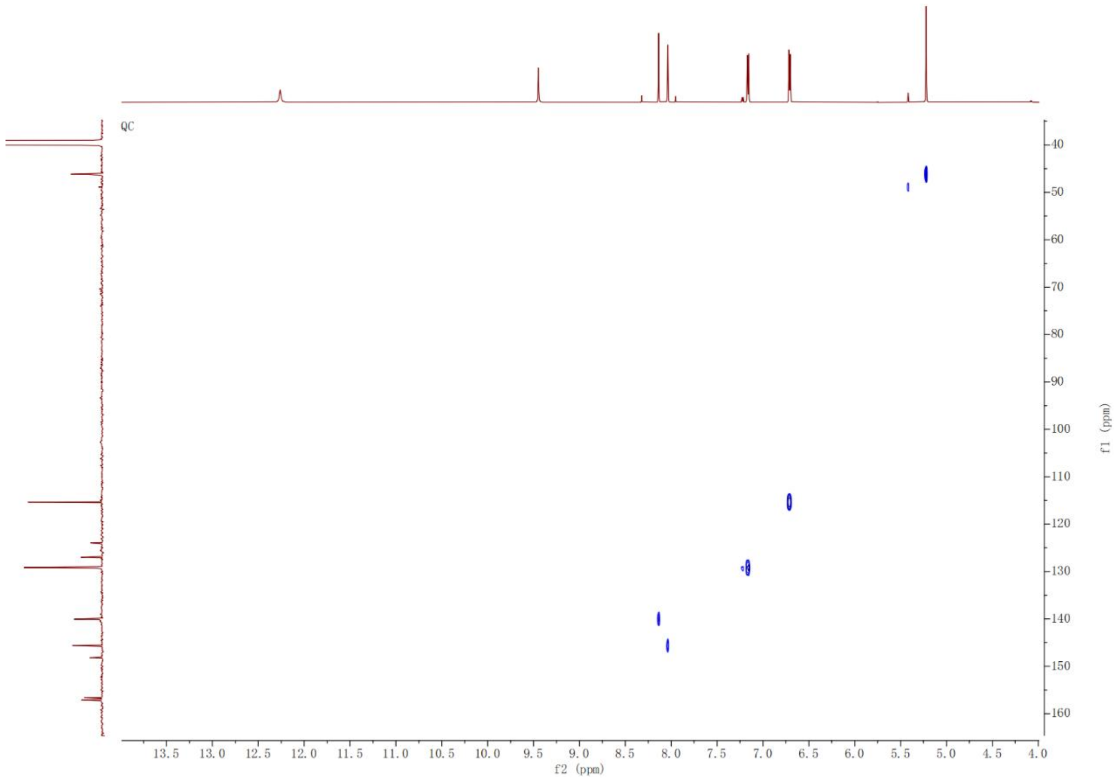

Figure S26. HSQC spectrum of **13**.

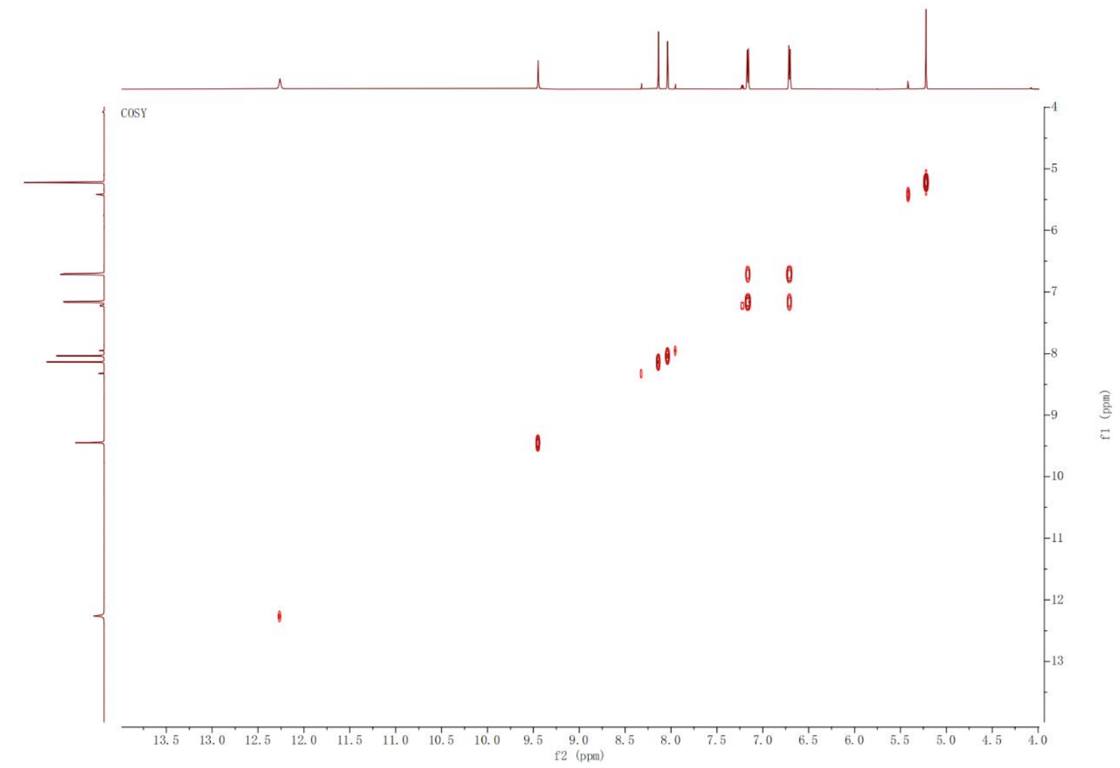

Figure S27. ^{1}H-^{1}H COSY spectrum of **13**.

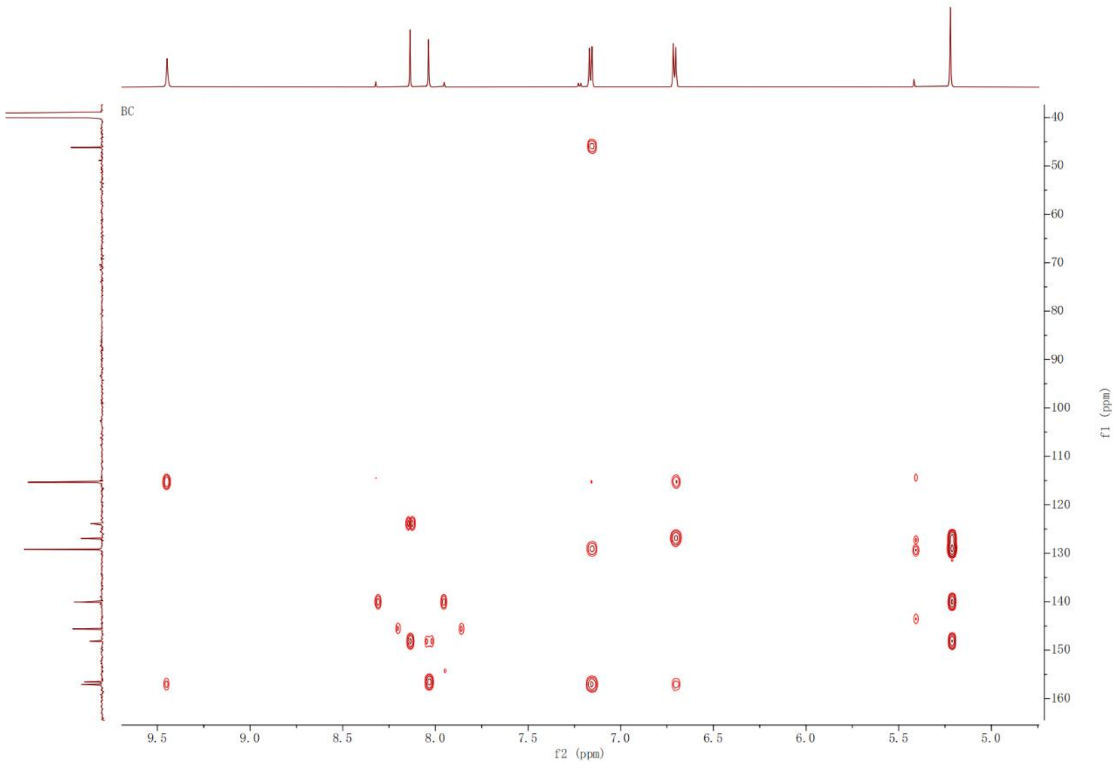

Figure S28. HMBC spectrum of **13**.

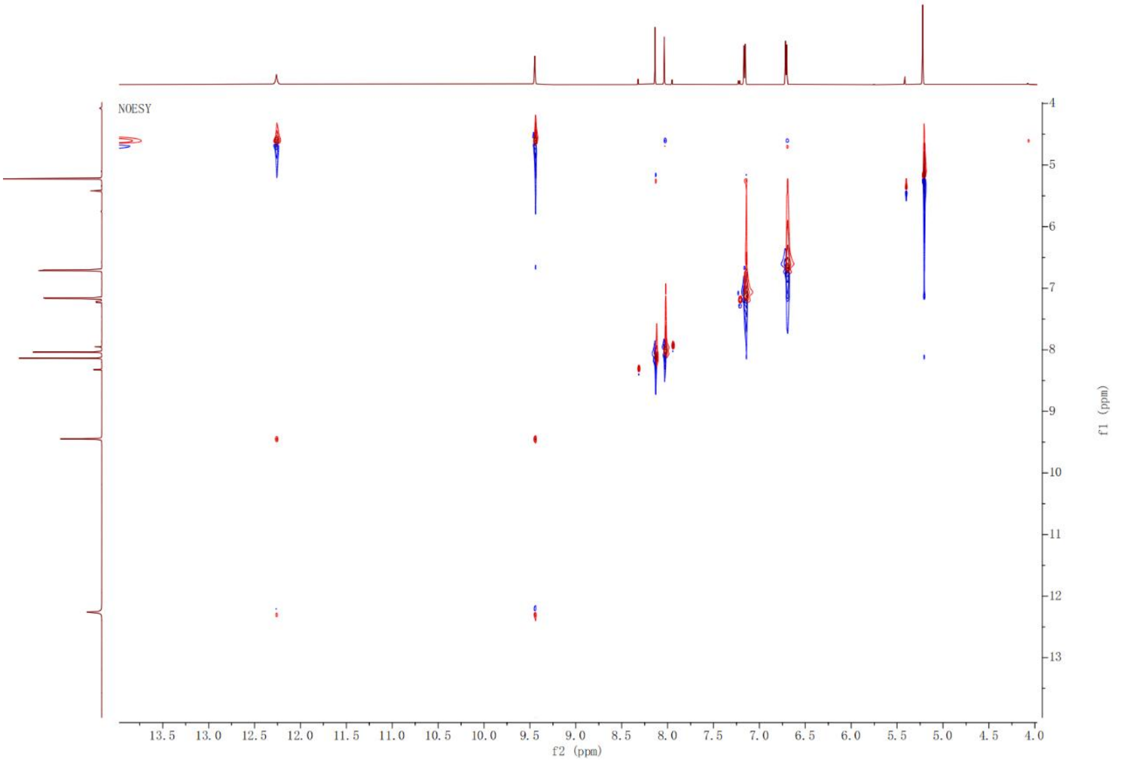

Figure S29. NOESY spectrum of **13**.

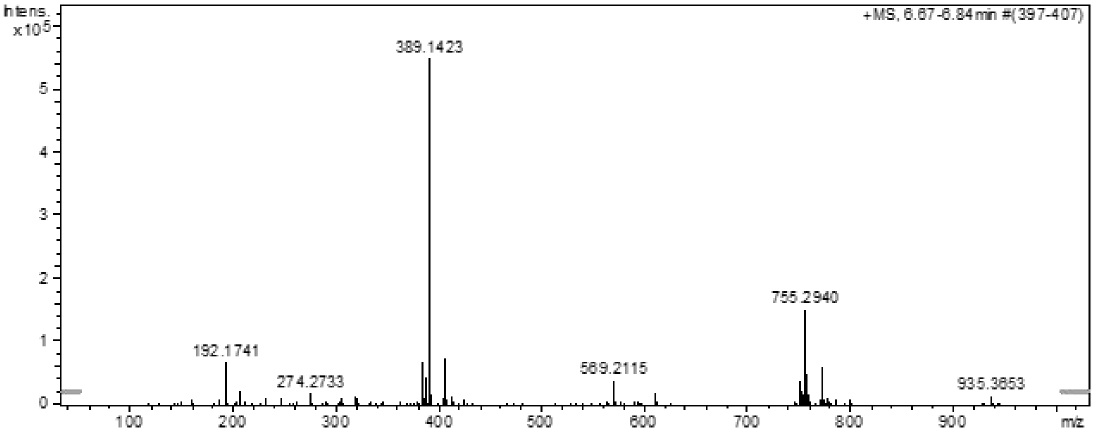

Meas. m/z	#	Formula	Score	m/z	err [mDa]	err [ppm]	mSigma	rdb	e⁻ Conf	N-Rule
389.1423	1	C 11 H 22 N 6 Na O 8	18.07	389.1391	-3.2	-8.2	1.5	3.5	even	ok
	2	C 8 H 14 N 16 Na O 2	3.22	389.1378	-4.5	-11.6	11	9.5	even	ok
	3	C 15 H 26 Na O 10	100	389.1418	-0.5	-1.3	16	2.5	even	ok
	4	C 12 H 18 N 10 Na O 4	43.97	389.1405	-1.8	-4.7	16.3	8.5	even	ok
	5	C 16 H 22 N 4 Na O 6	64.35	389.1432	0.8	2.2	28.1	7.5	even	ok
	6	C 13 H 14 N 14 Na	70.51	389.1418	-0.5	-1.3	31.3	13.5	even	ok
	7	C 17 H 18 N 8 Na O 2	17.97	389.1445	2.2	5.6	42.9	12.5	even	ok
	8	C 21 H 22 N 2 Na O 4	0.67	389.1472	4.9	12.5	55.2	11.5	even	ok

Figure S30 HRESIMS spectrum of **17**.

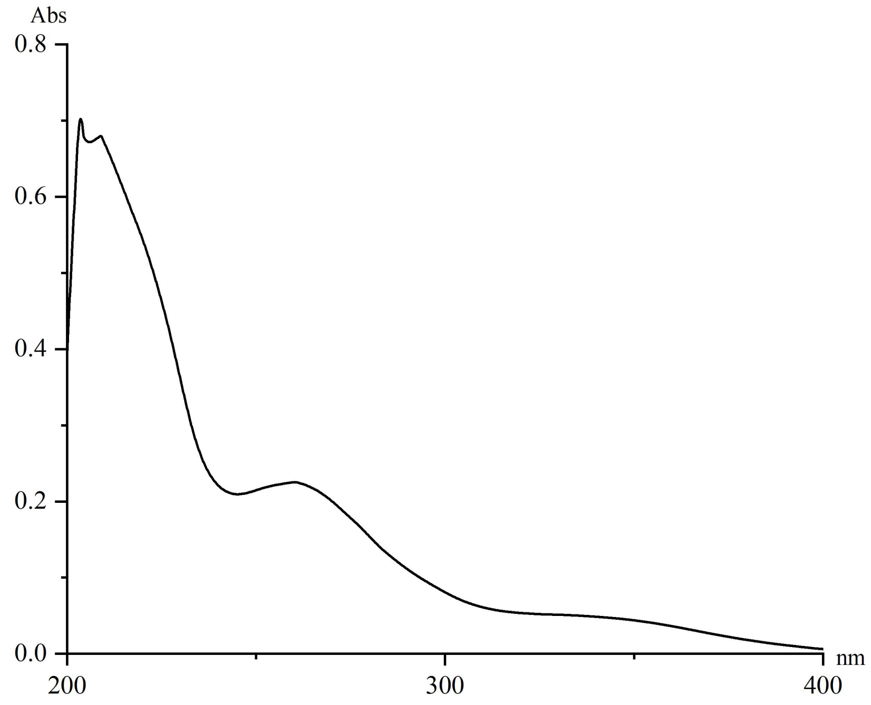

Figure S31 UV spectrum of **17**.

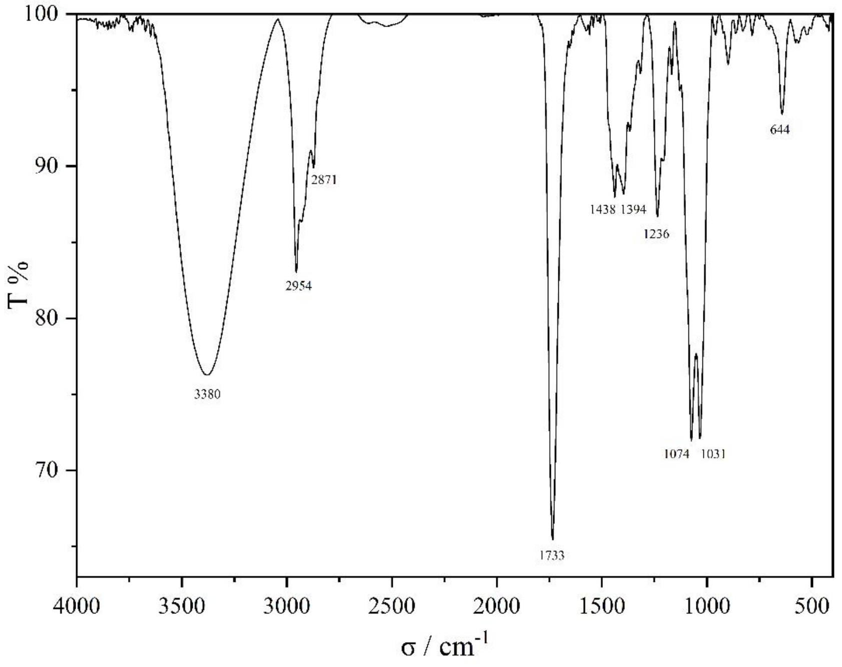

Figure S32 IR spectrum of **17**.

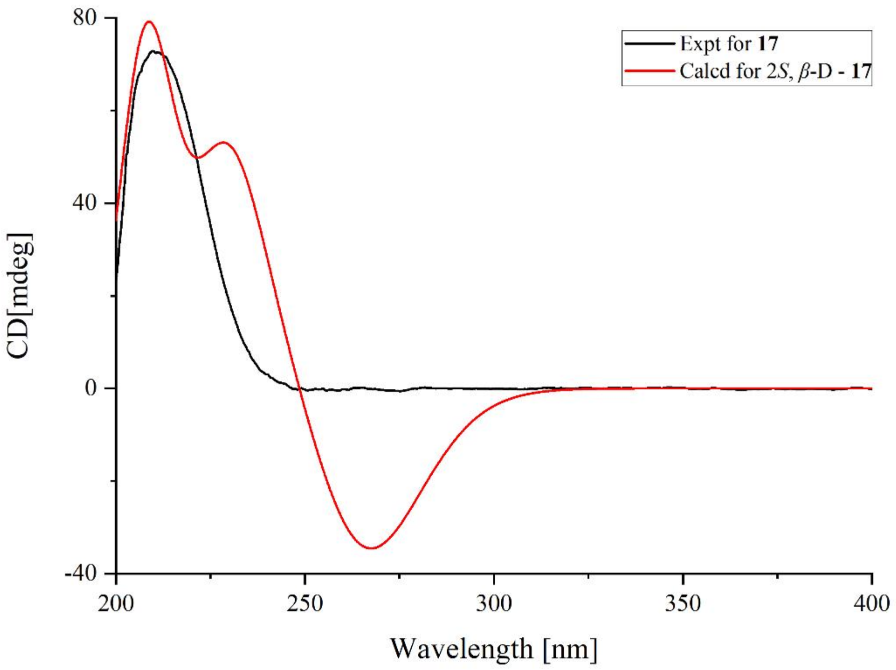

Figure S33 CD spectrum of **17**.

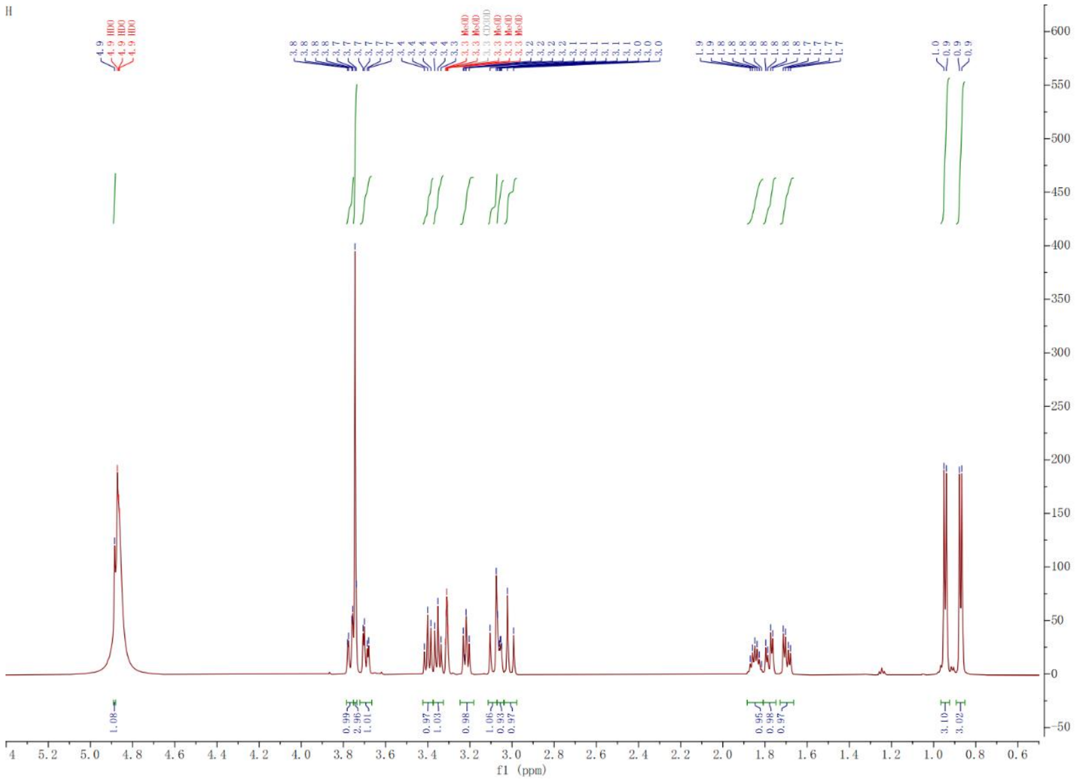

Figure S34 ^{1}H NMR spectrum of **17**.

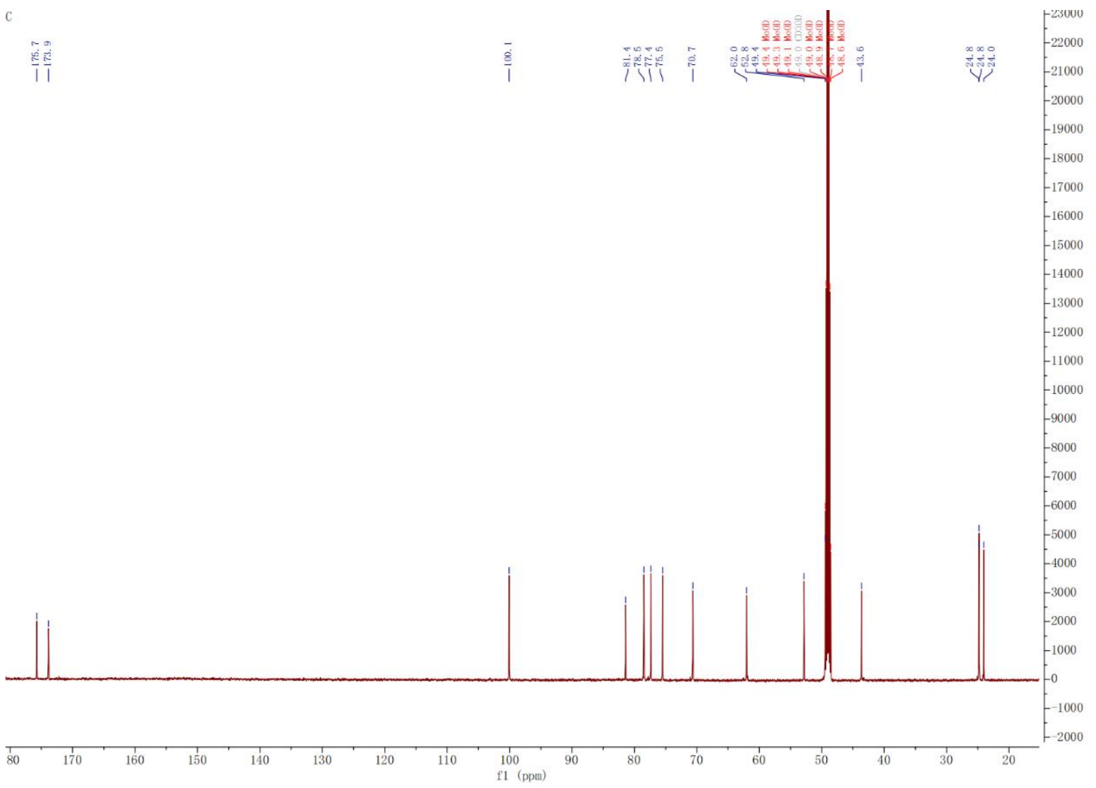

Figure S35 ^{13}C NMR spectrum of **17**.

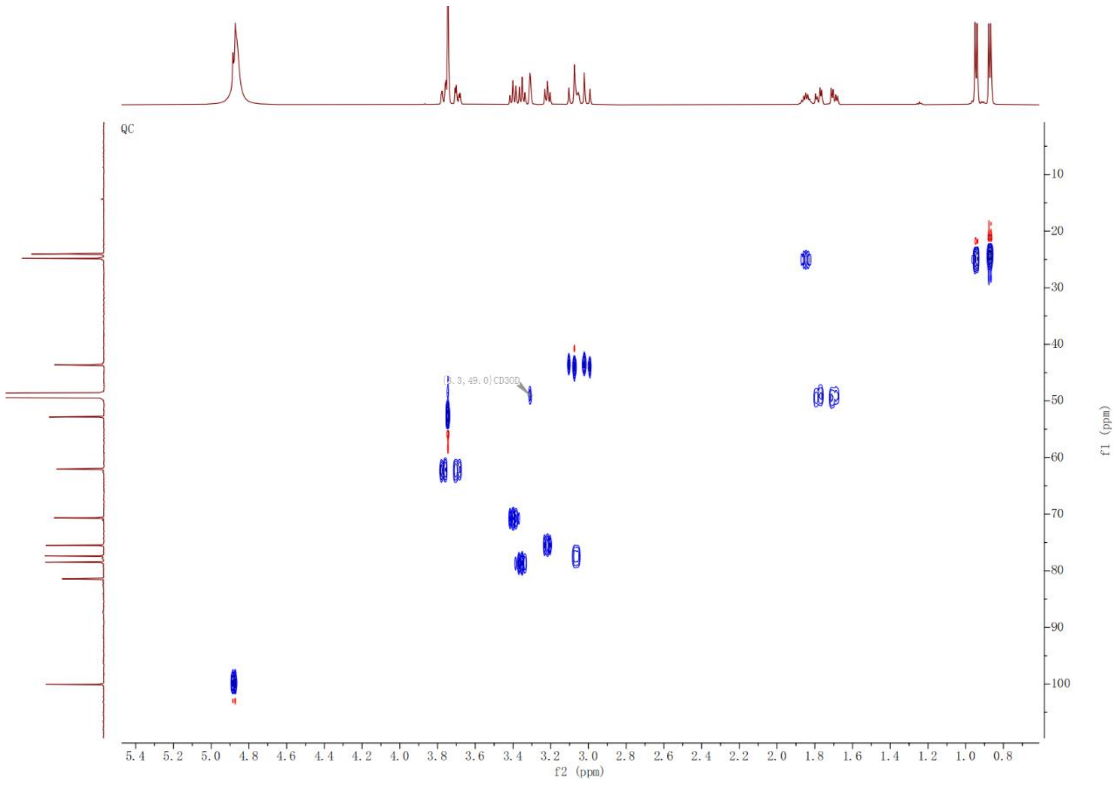

Figure S36 HSQC spectrum of **17**.

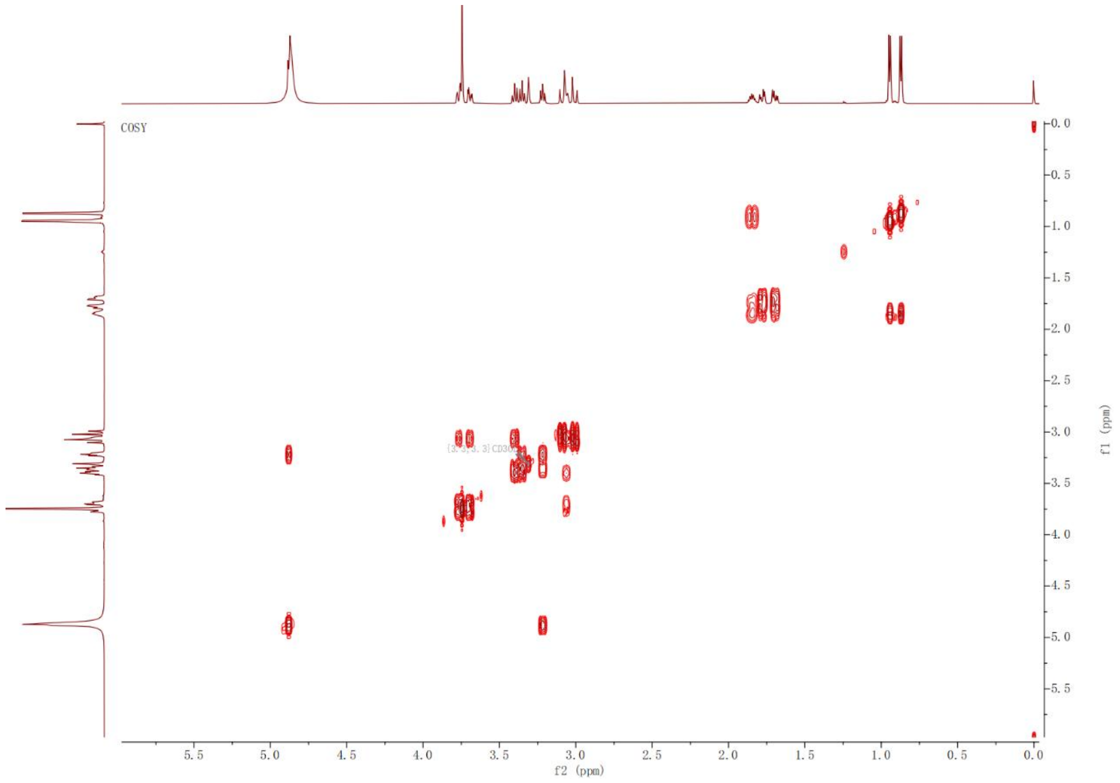

Figure S37 ^{1}H-^{1}H COSY spectrum of **17**.

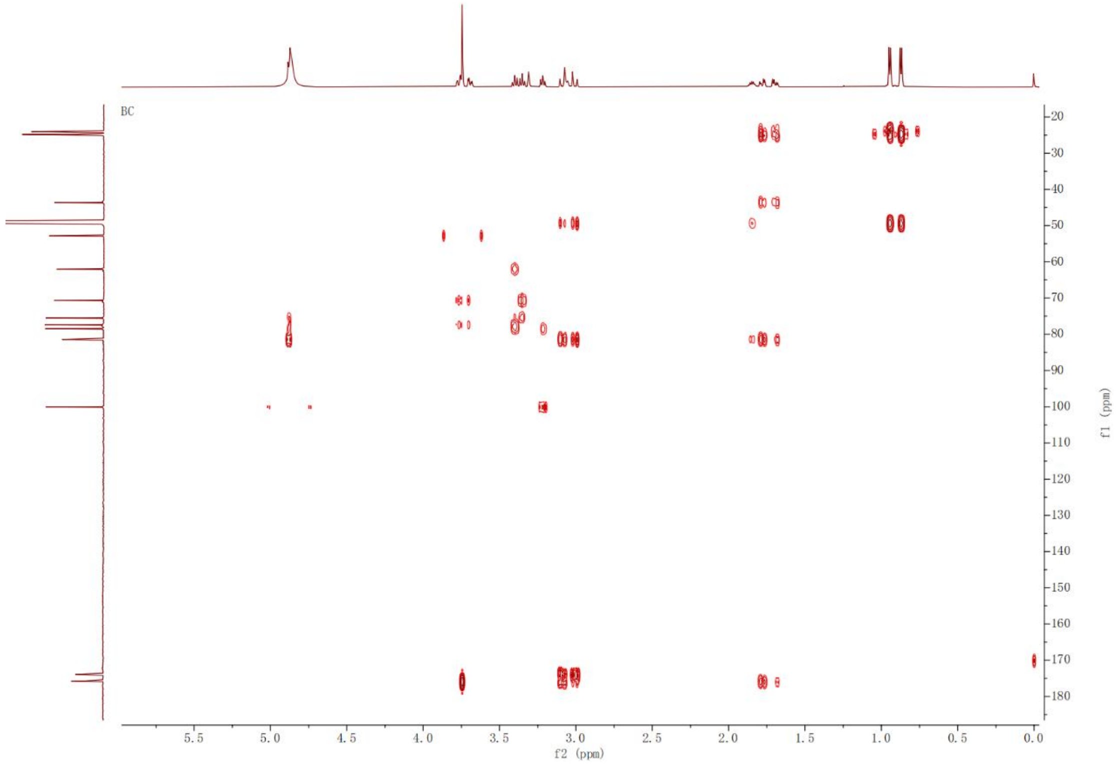

Figure S38 HMBC spectrum of **17**.

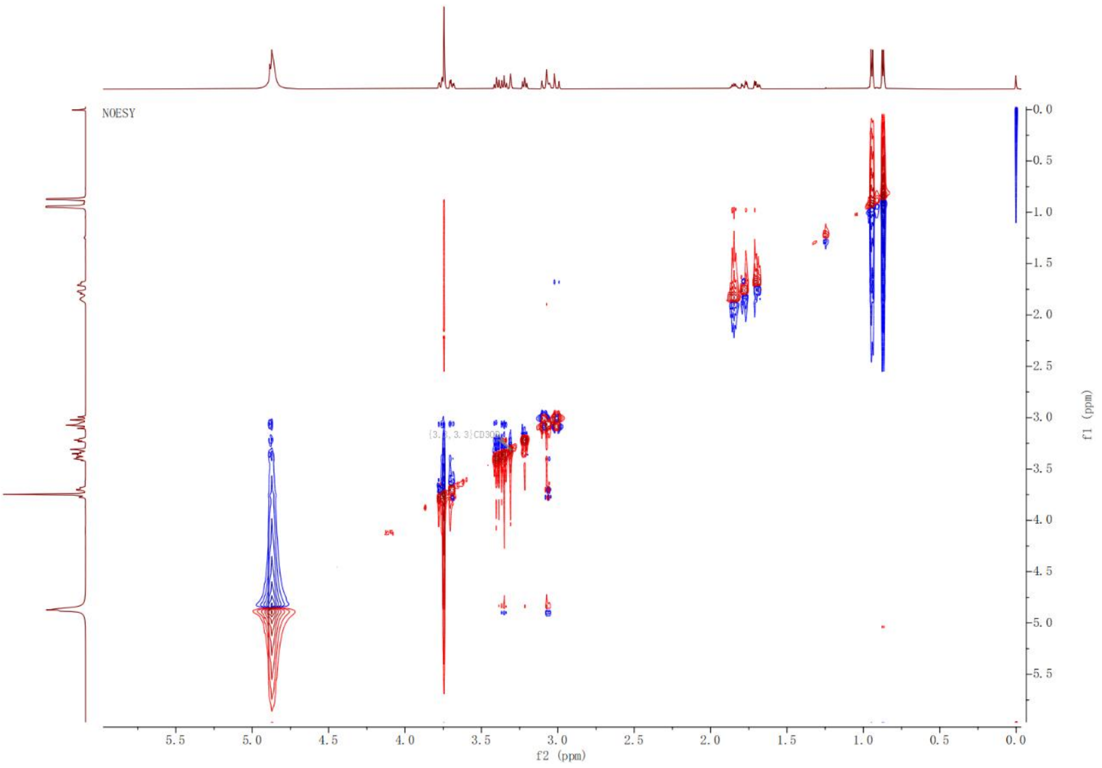

Figure S39 NOESY spectrum of **17**.

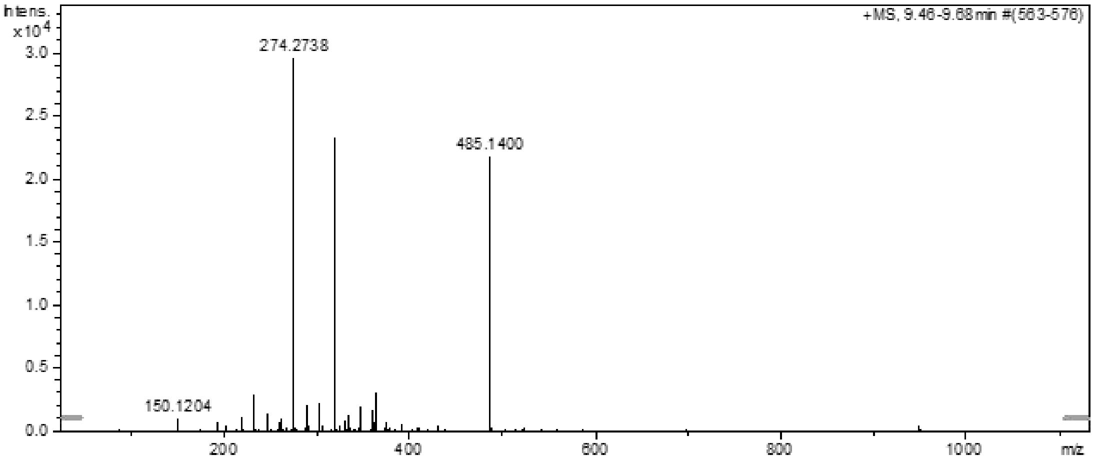

Figure S40. HRESIMS spectrum of **3**.

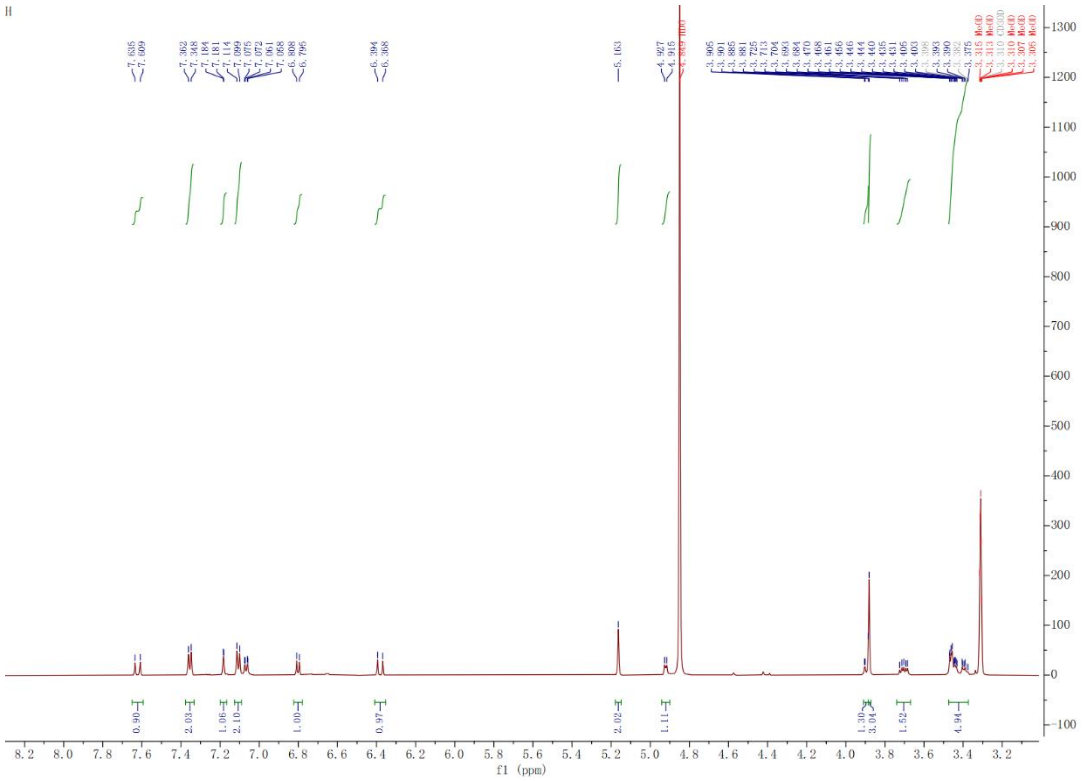

Figure S41. ^{1}H NMR spectrum of **3**.

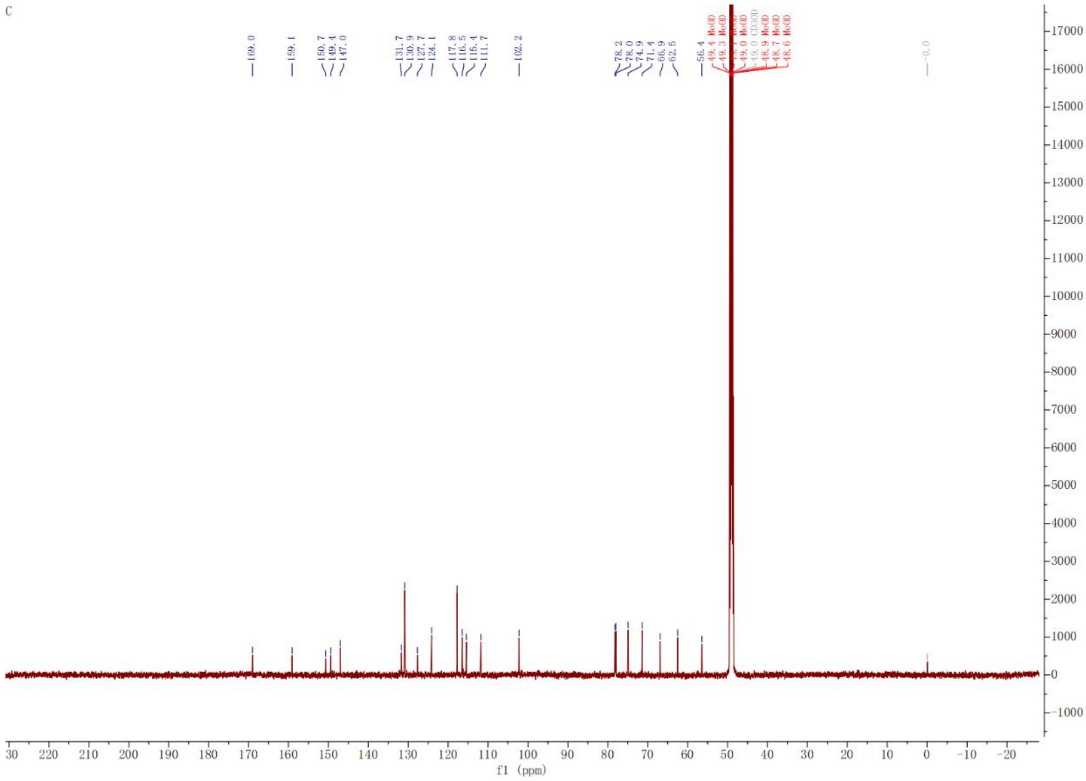

Figure S42. ^{13}C NMR spectrum of **3**.

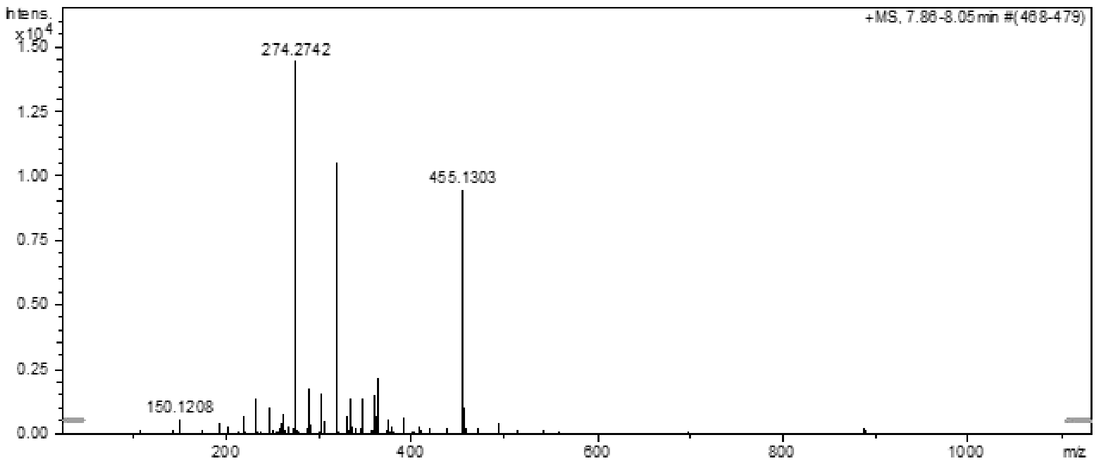

Figure S43. HRESIMS spectrum of **4**.

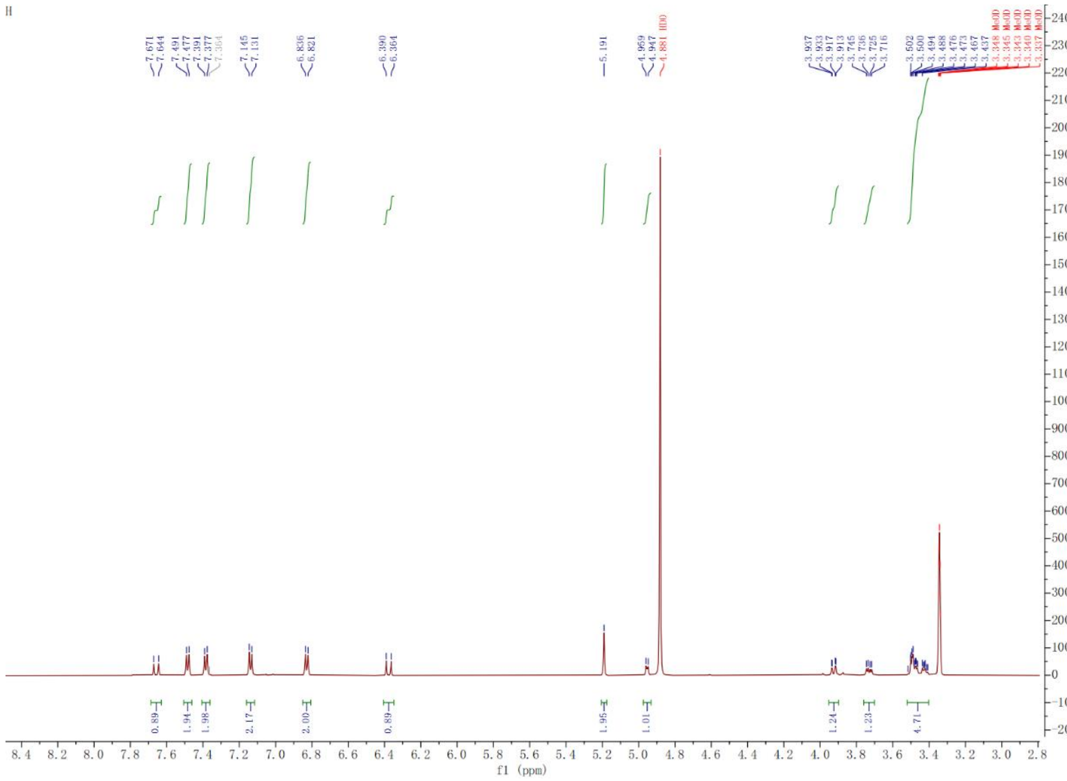

Figure S44. ^{1}H NMR spectrum of **4**.

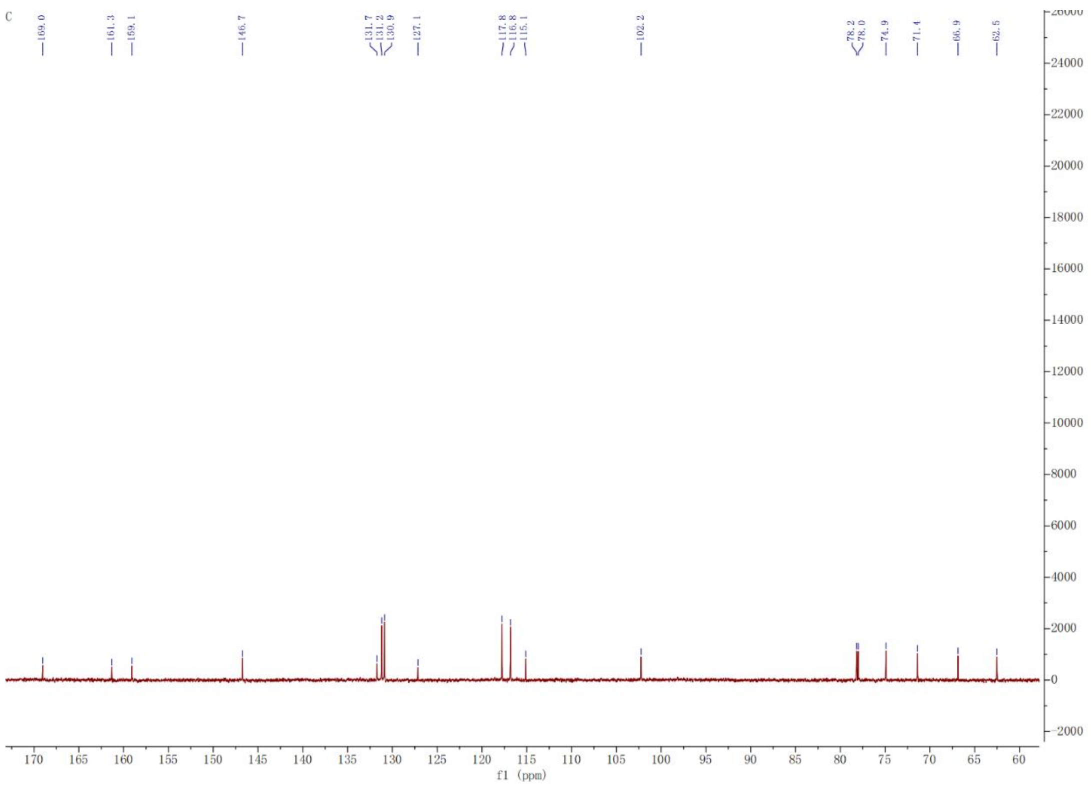

Figure S45. ^{13}C NMR spectrum of **4**.

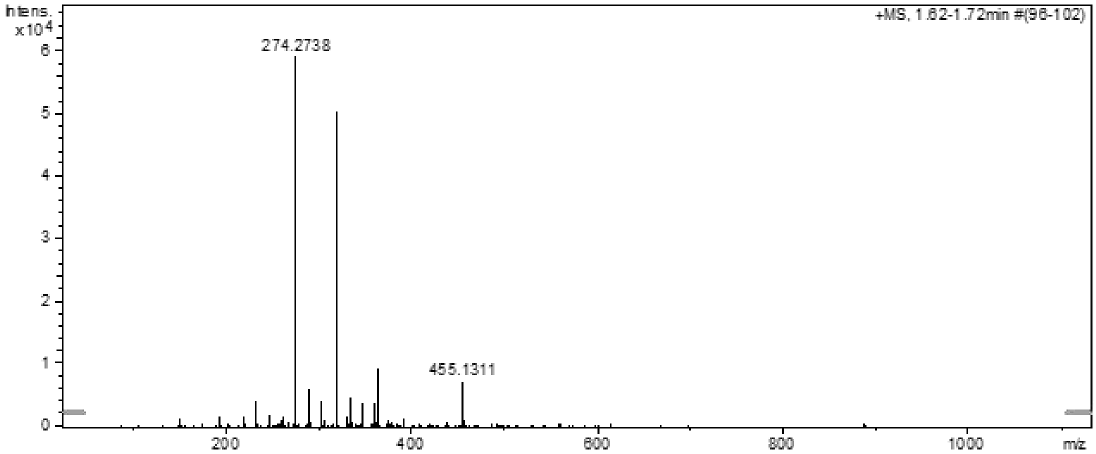

Figure S46. HRESIMS spectrum of **5**.

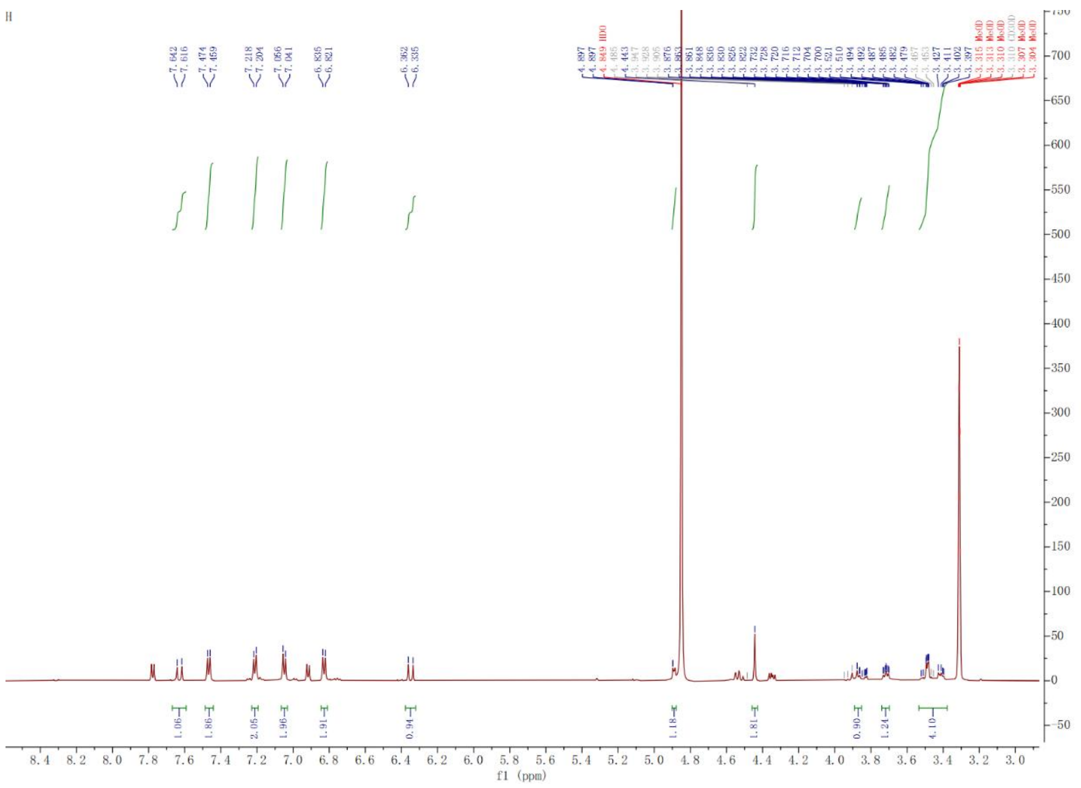

Figure S47. ^{1}H NMR spectrum of **5**.

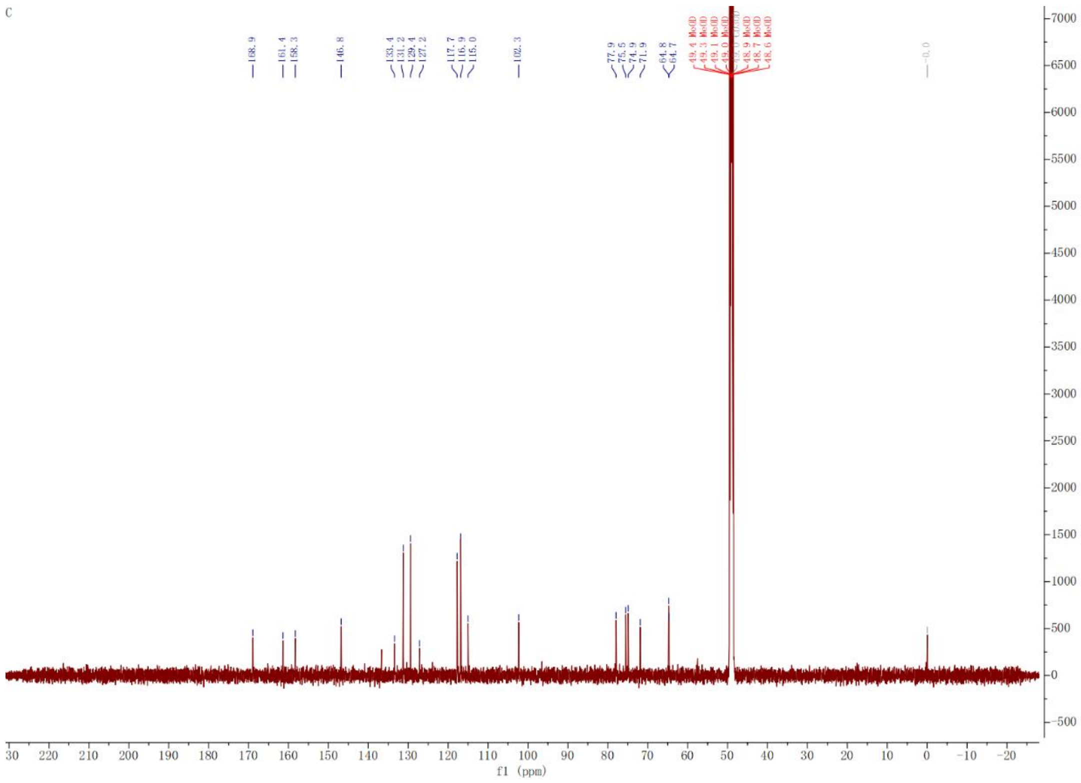

Figure S48. ^{13}C NMR spectrum of **5**.

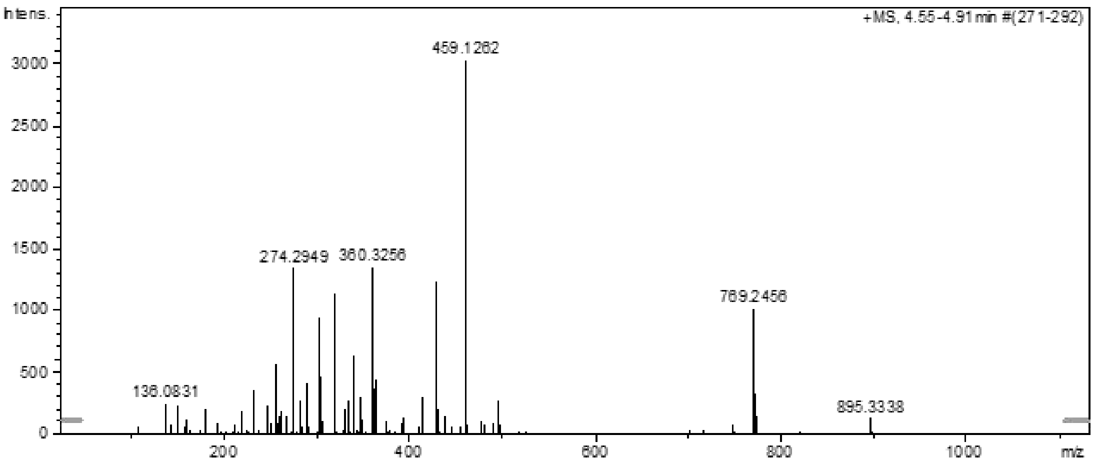

Figure S49. HRESIMS spectrum of **6**.

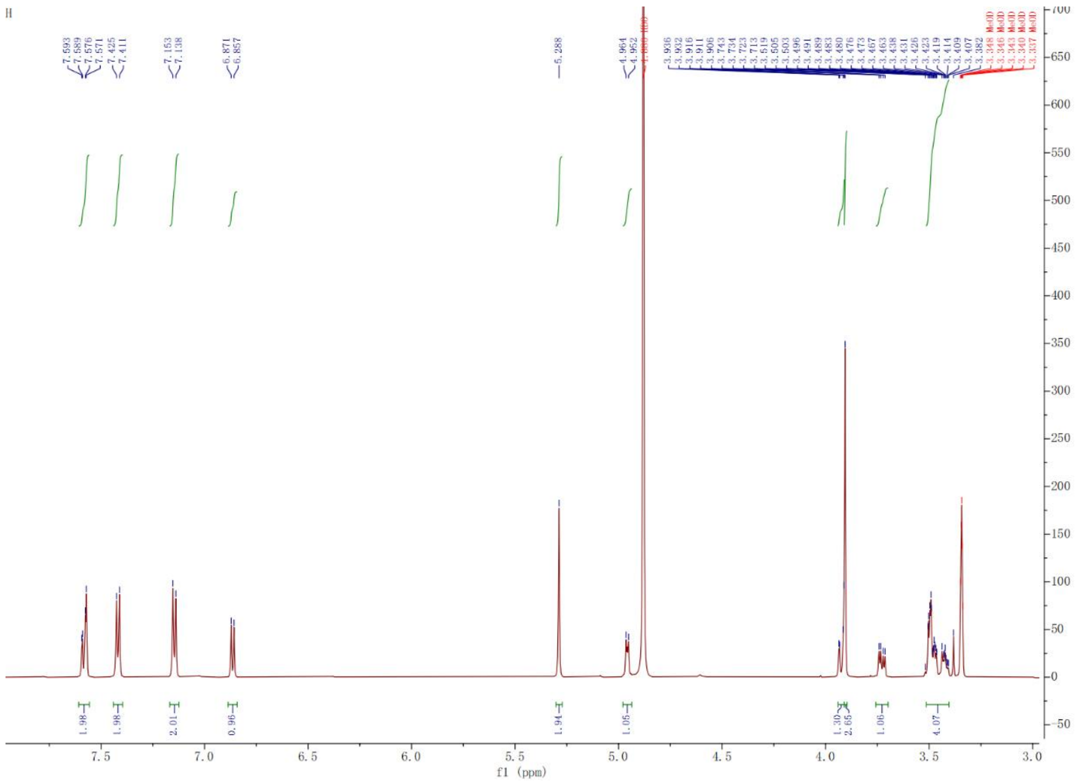

Figure S50. ^{1}H NMR spectrum of **6**.

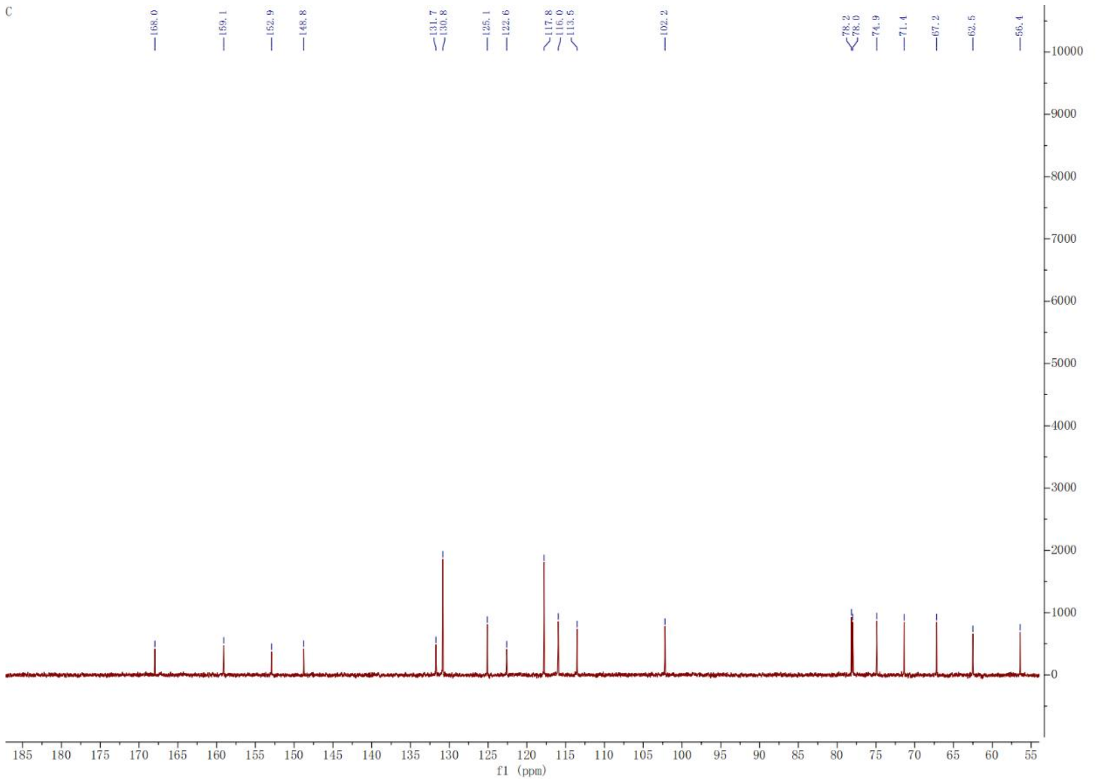

Figure S51. ^{13}C NMR spectrum of **6**.

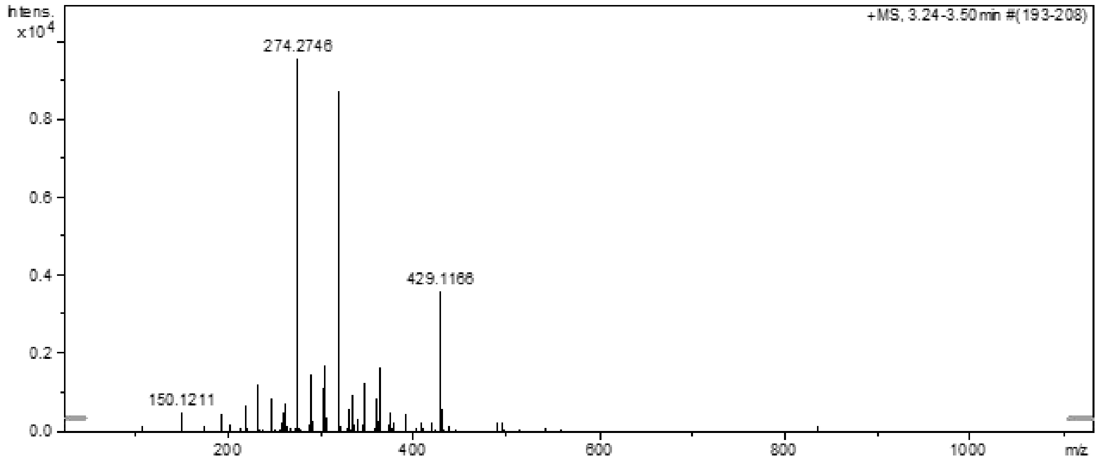

Figure S52. HRESIMS spectrum of **7**.

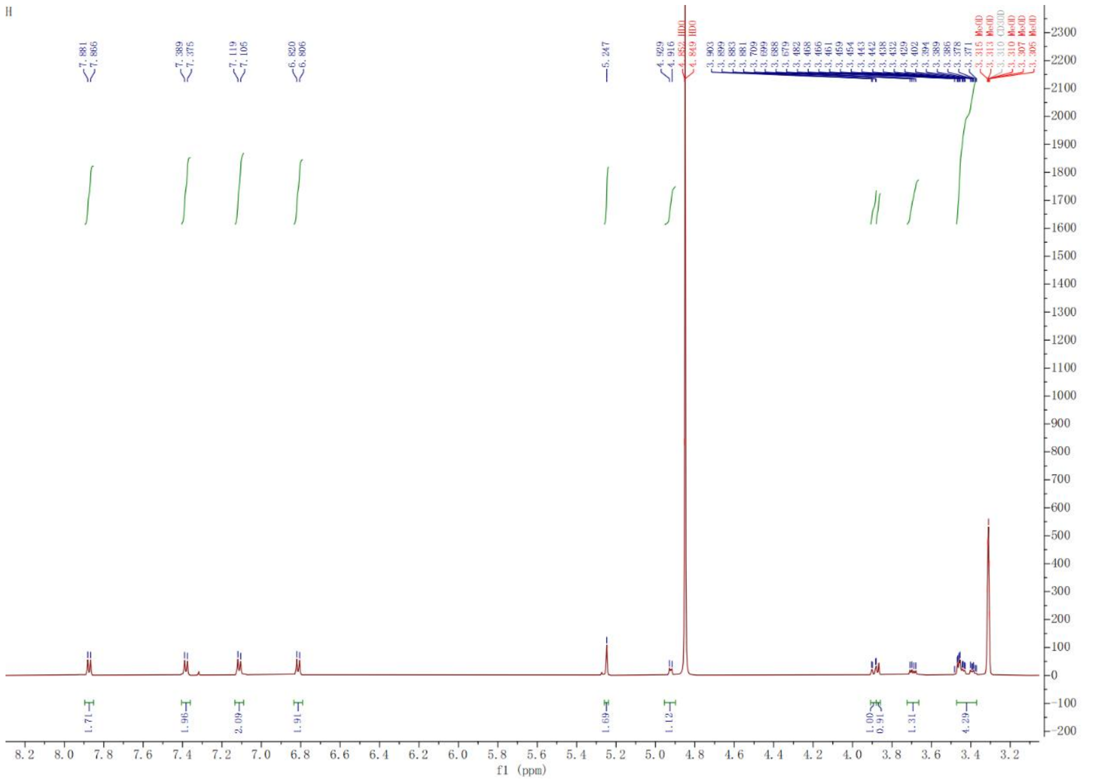

Figure S53. ^{1}H NMR spectrum of **7**.

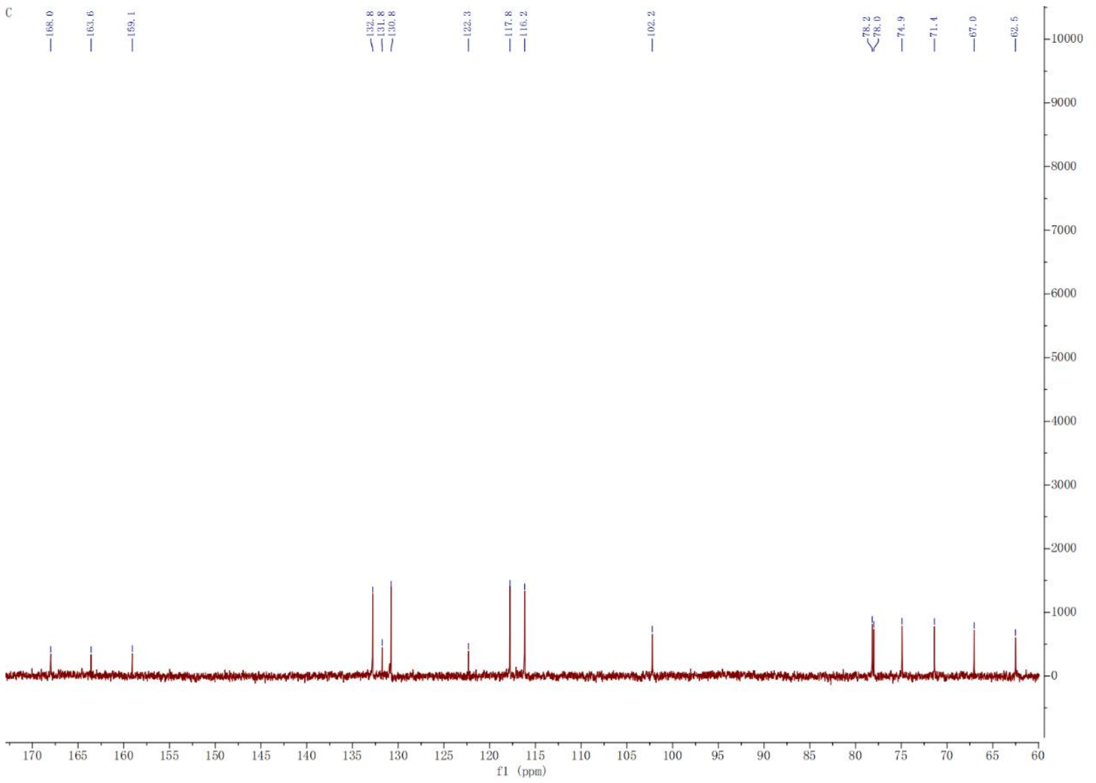

Figure S54. ^{13}C NMR spectrum of **7**.

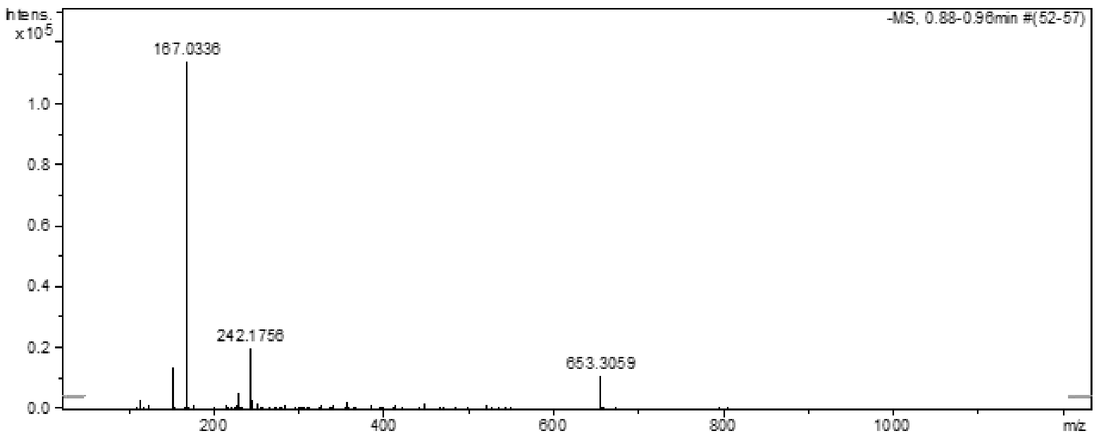

Figure S55. HRESIMS spectrum of **8**.

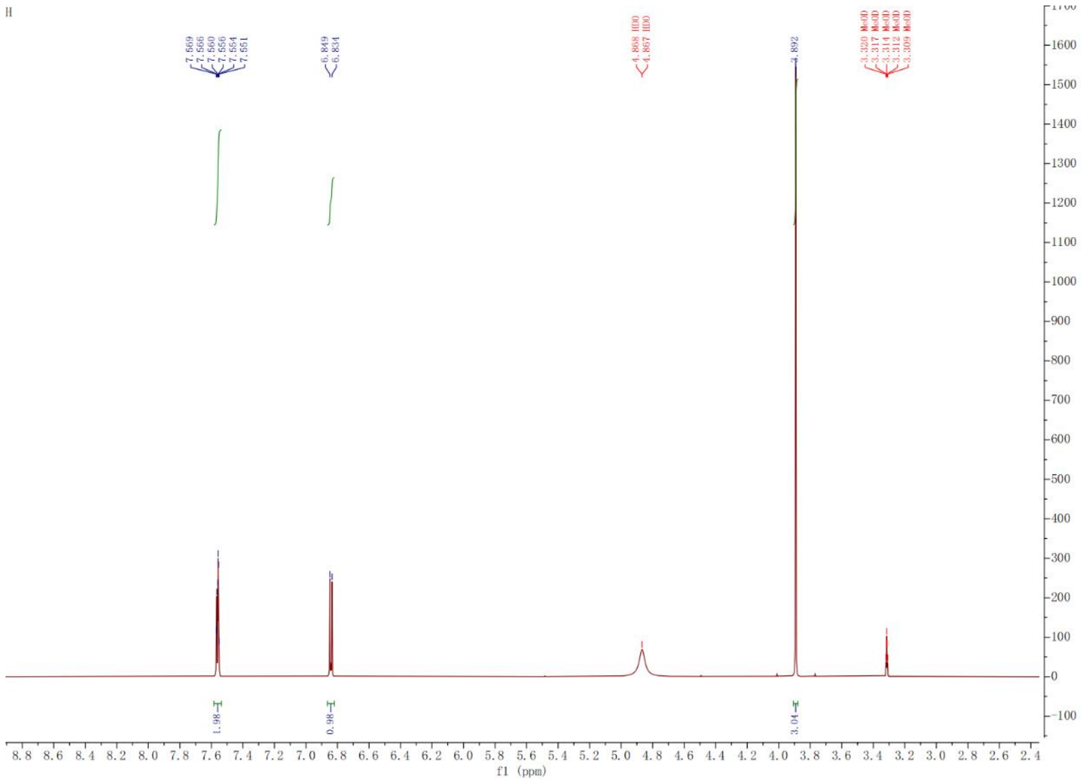

Figure S56. ^{1}H NMR spectrum of **8**.

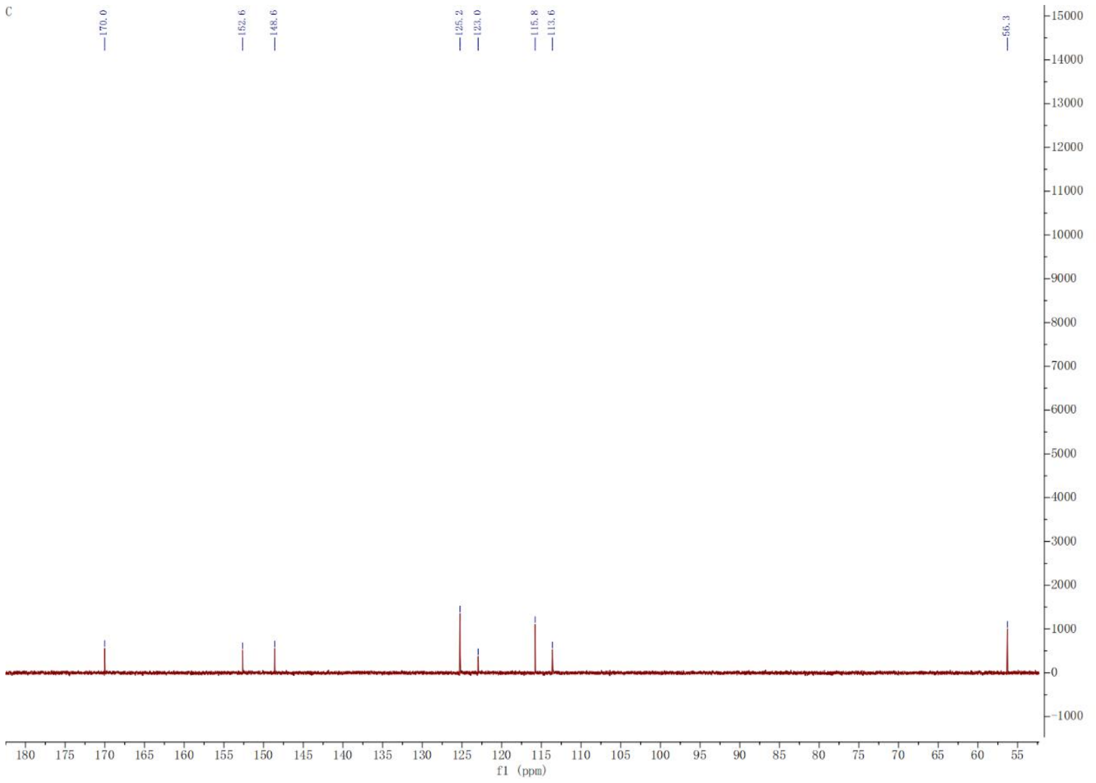

Figure S57. ^{13}C NMR spectrum of **8**.

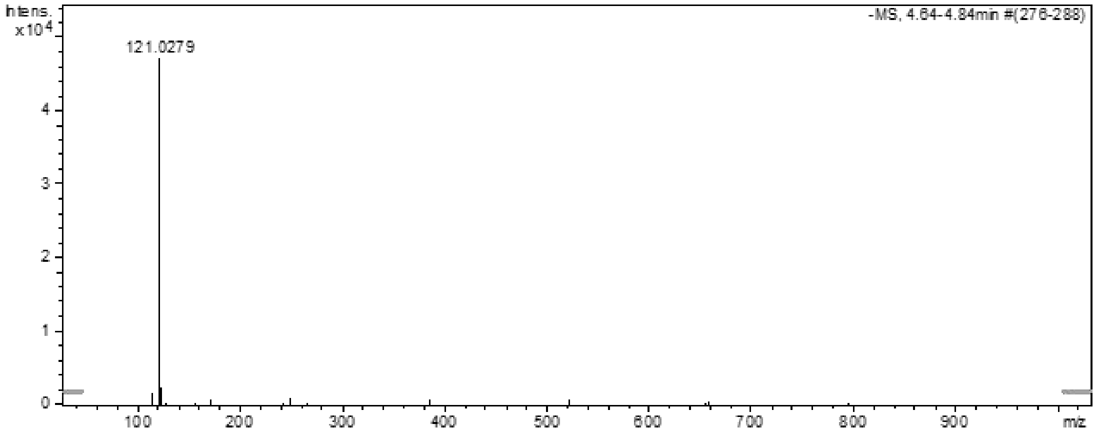

Figure S58. HRESIMS spectrum of **9**.

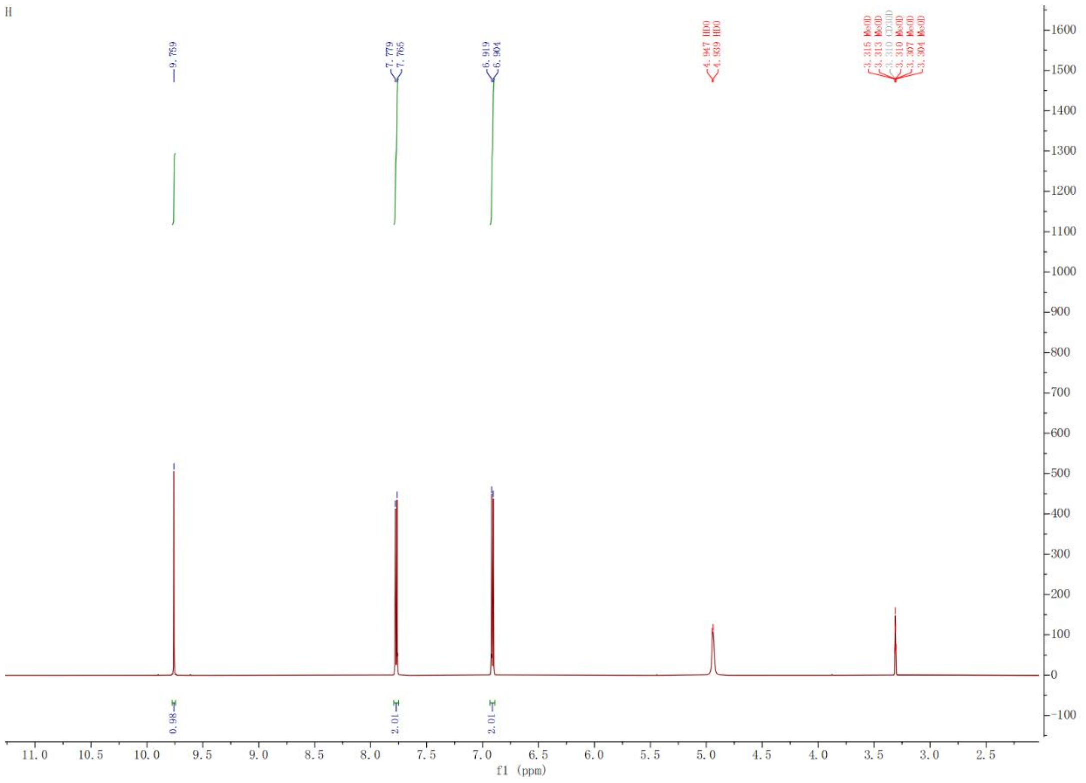

Figure S59. ^{1}H NMR spectrum of **9**.

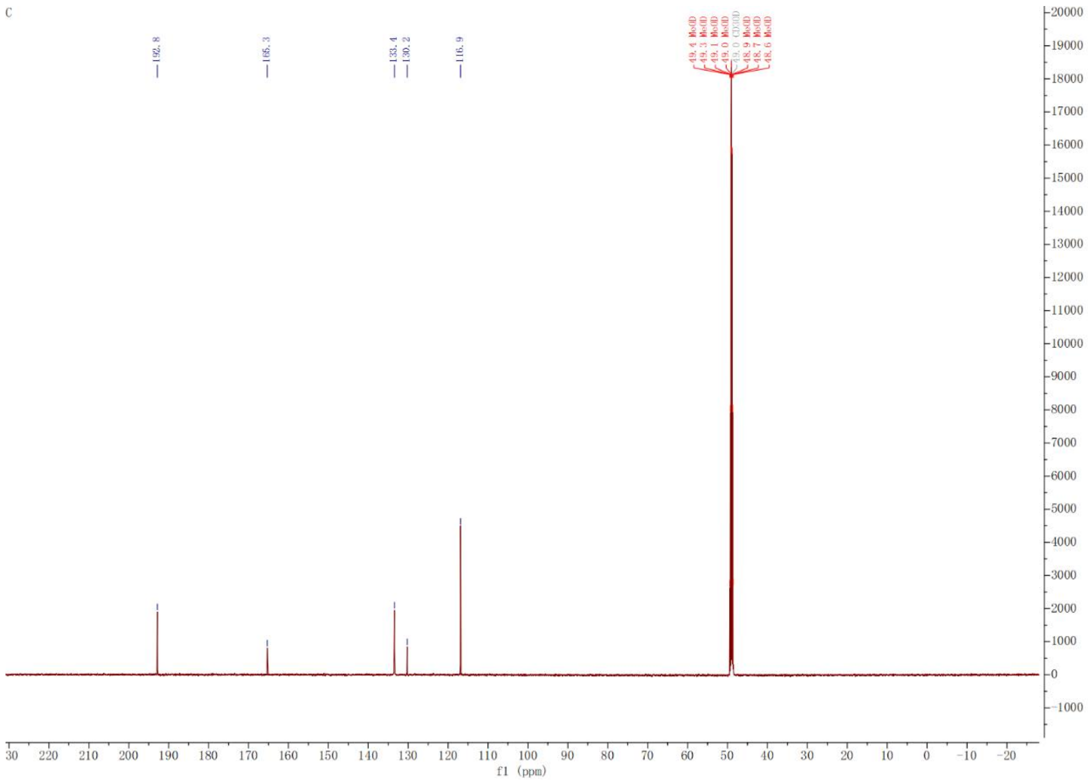

Figure S60. ^{13}C NMR spectrum of **9**.

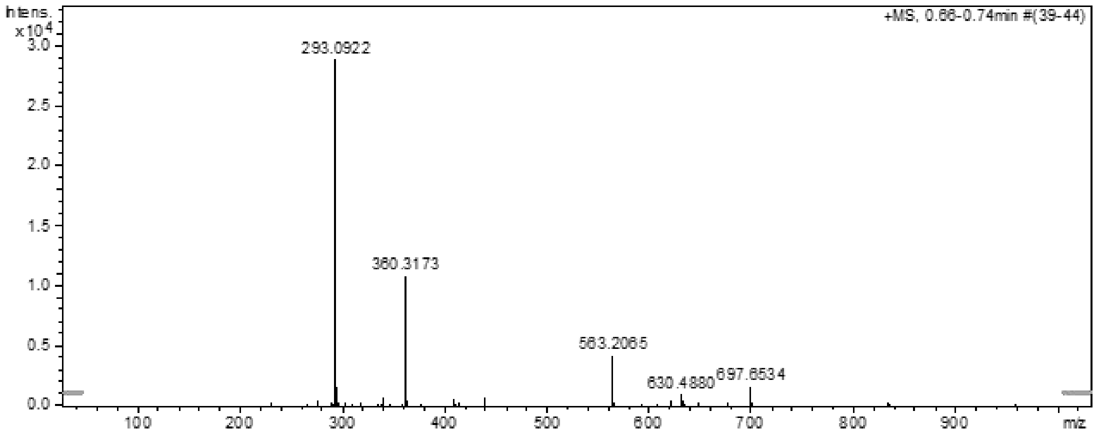

Figure S61. HRESIMS spectrum of **10**.

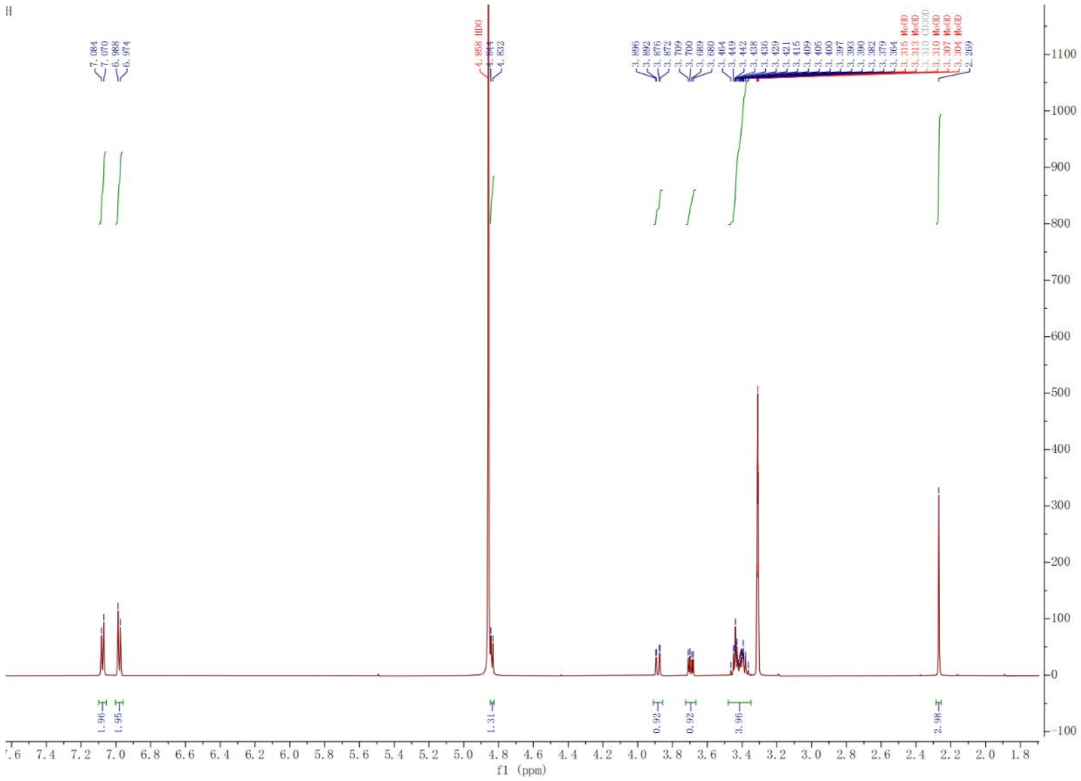

Figure S62. ^{1}H NMR spectrum of **10**.

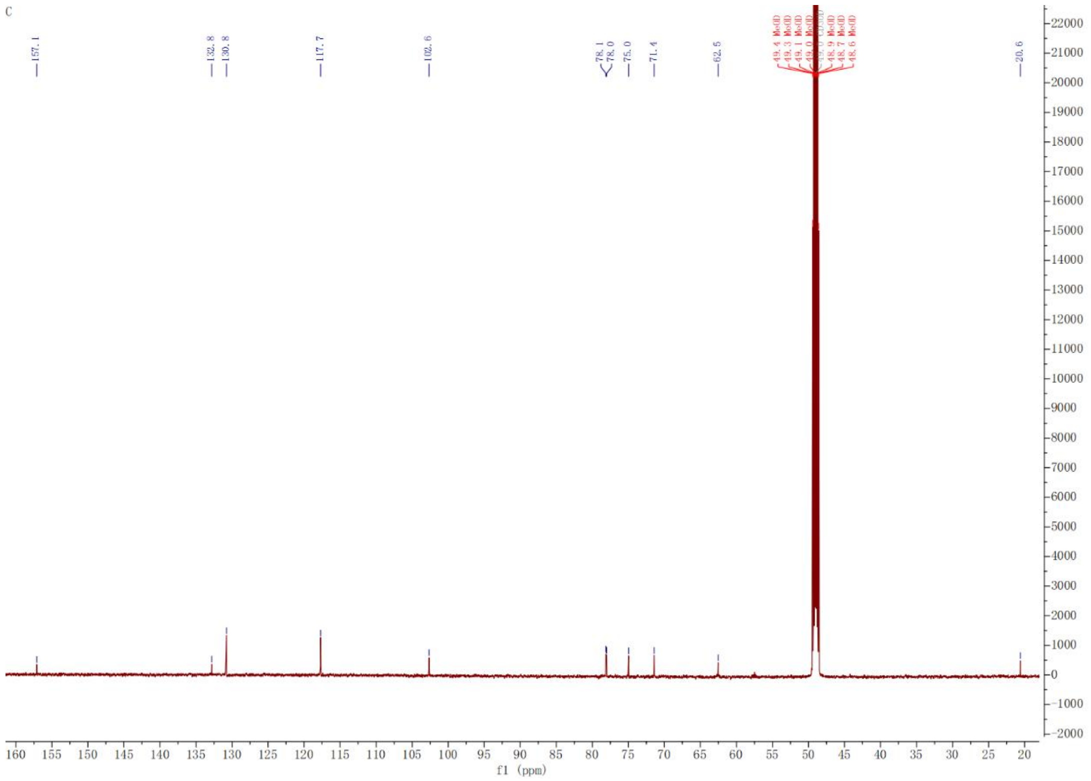

Figure S63. ^{13}C NMR spectrum of **10**.

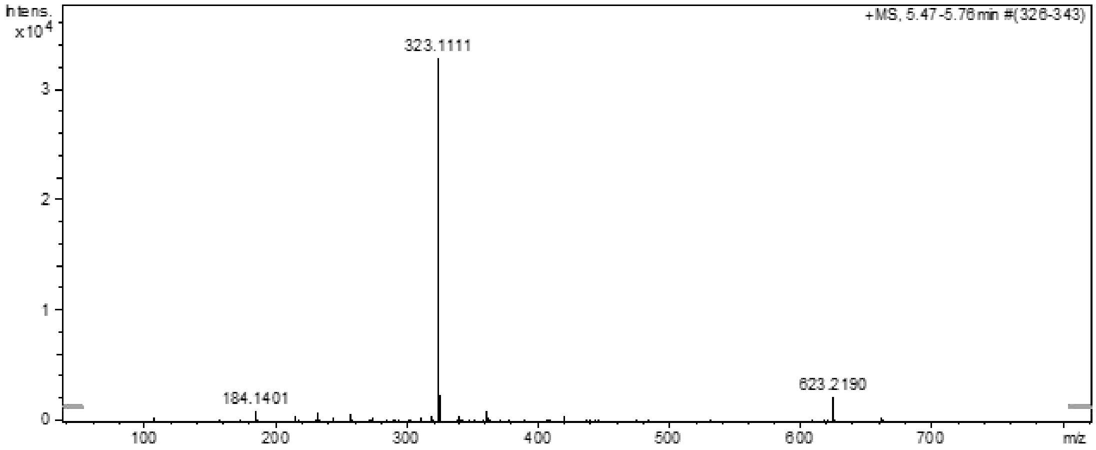

Figure S64. HRESIMS spectrum of **11**.

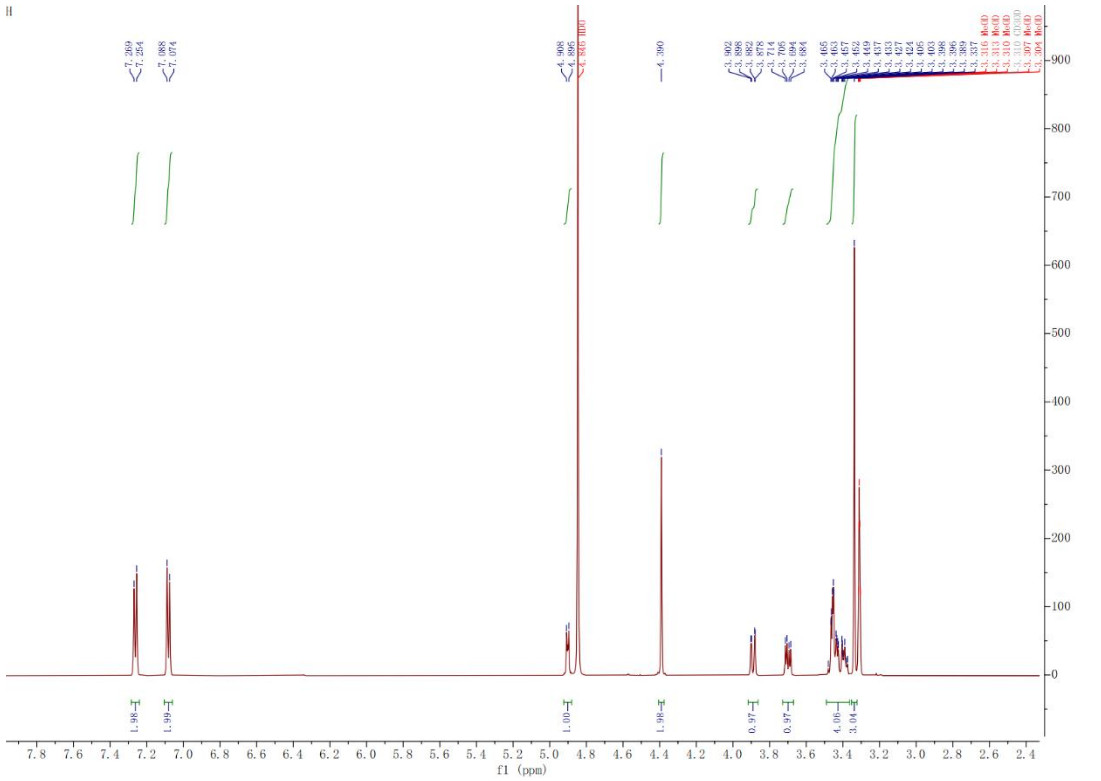

Figure S65. ^{1}H NMR spectrum of **11**.

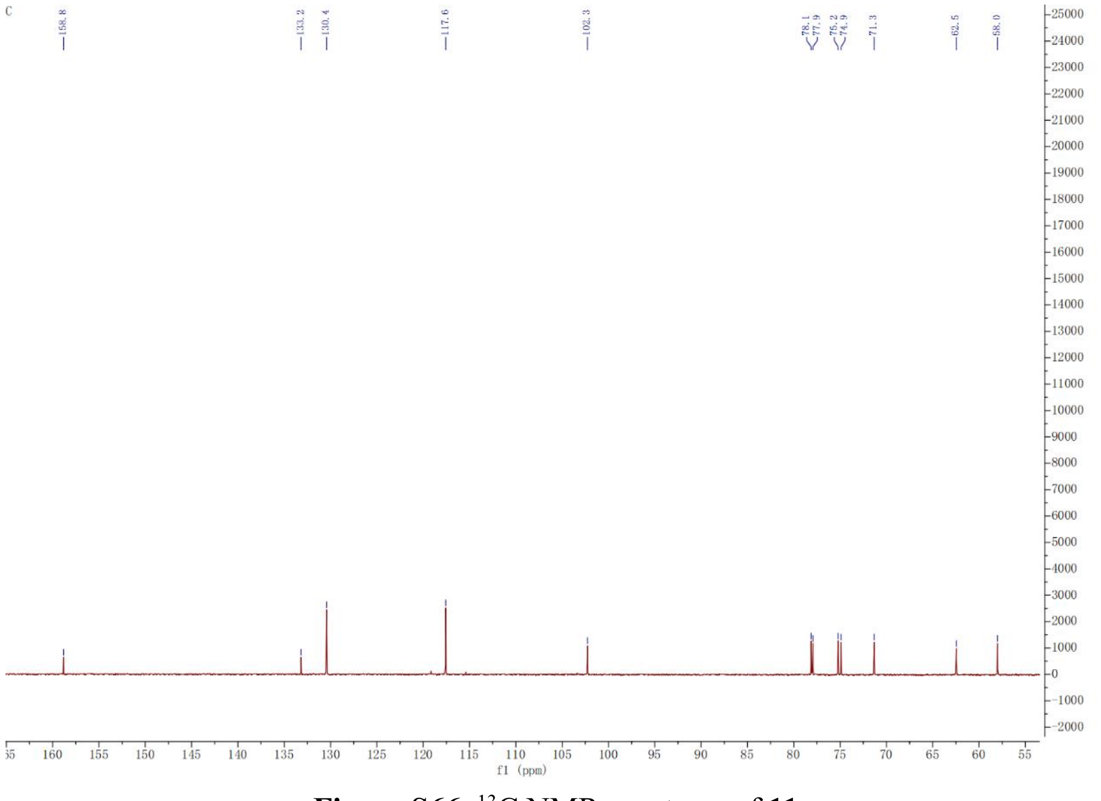

Figure S66. ^{13}C NMR spectrum of **11**.

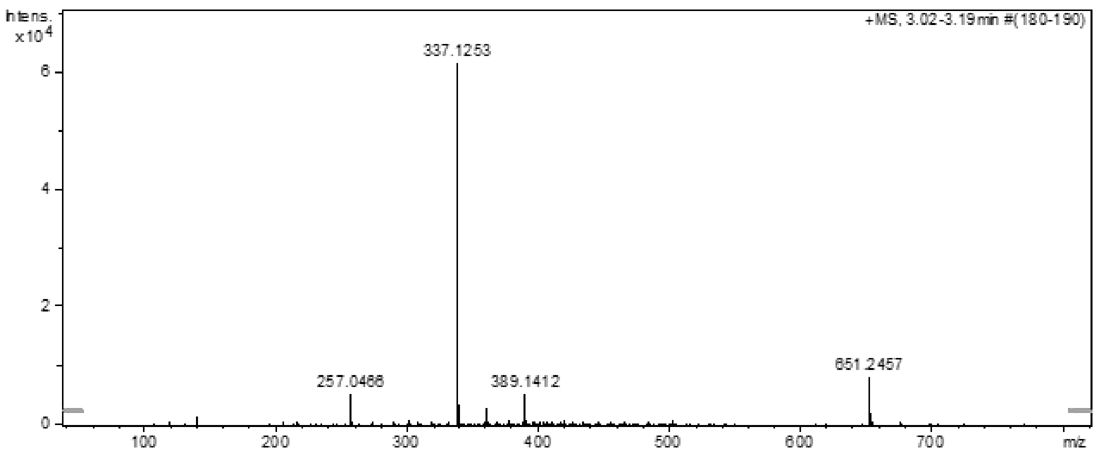

Figure S67. HRESIMS spectrum of **12**.

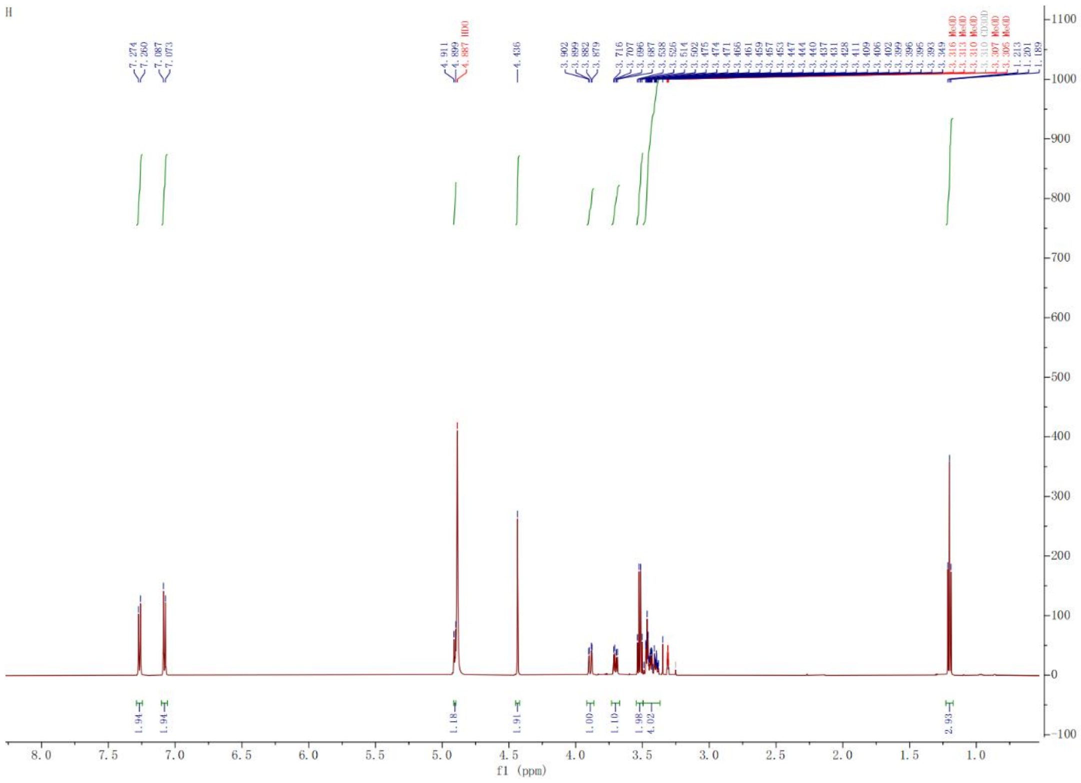

Figure S68. ^{1}H NMR spectrum of **12**.

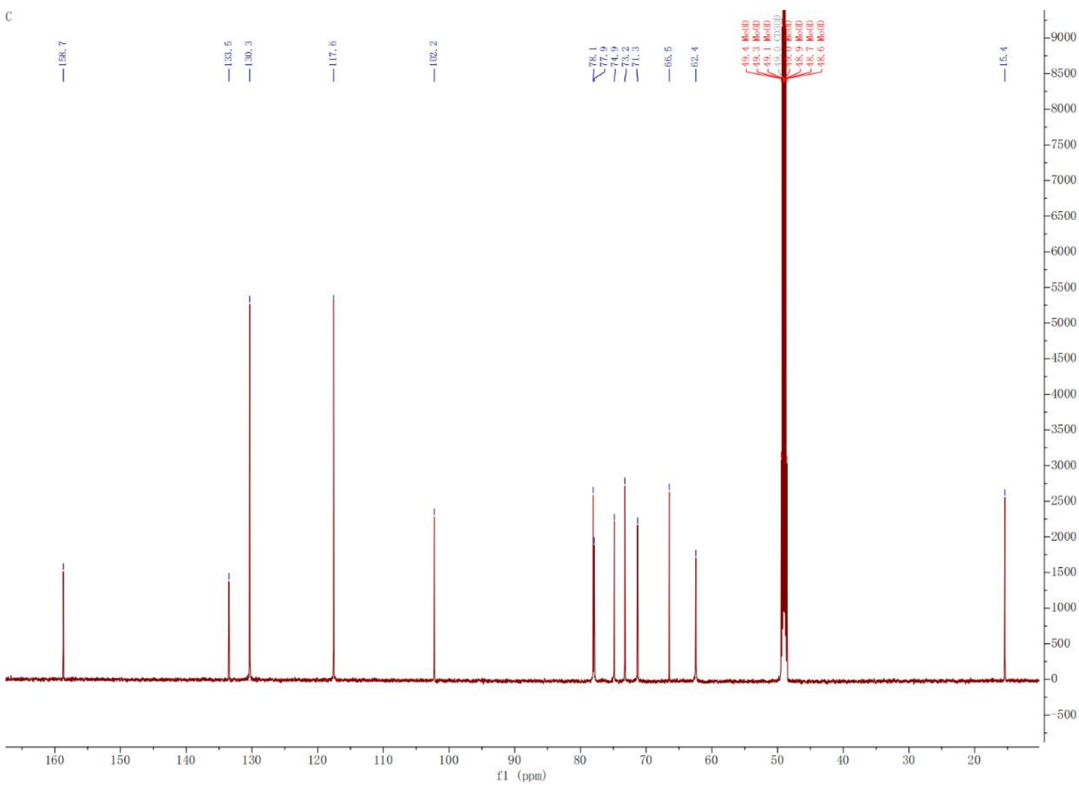

Figure S69. ^{13}C NMR spectrum of **12**.

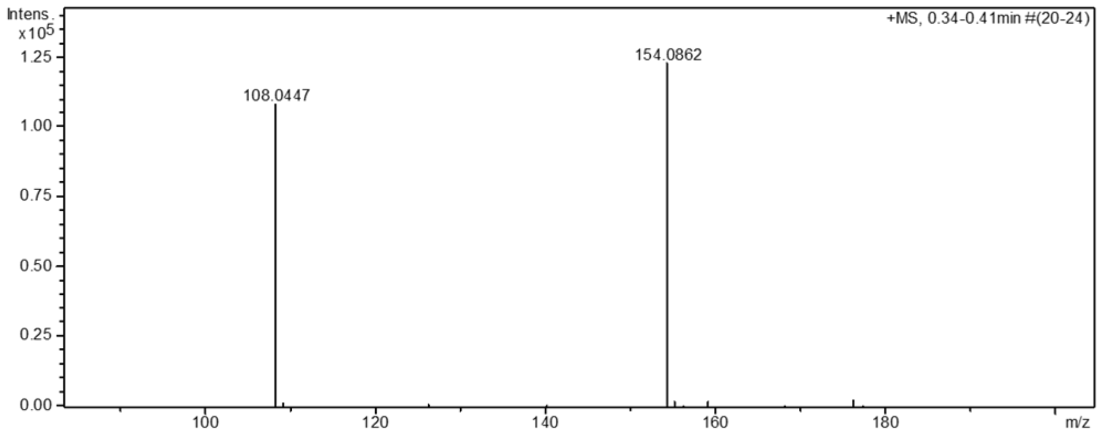

Figure S70. HRESIMS spectrum of **14**.

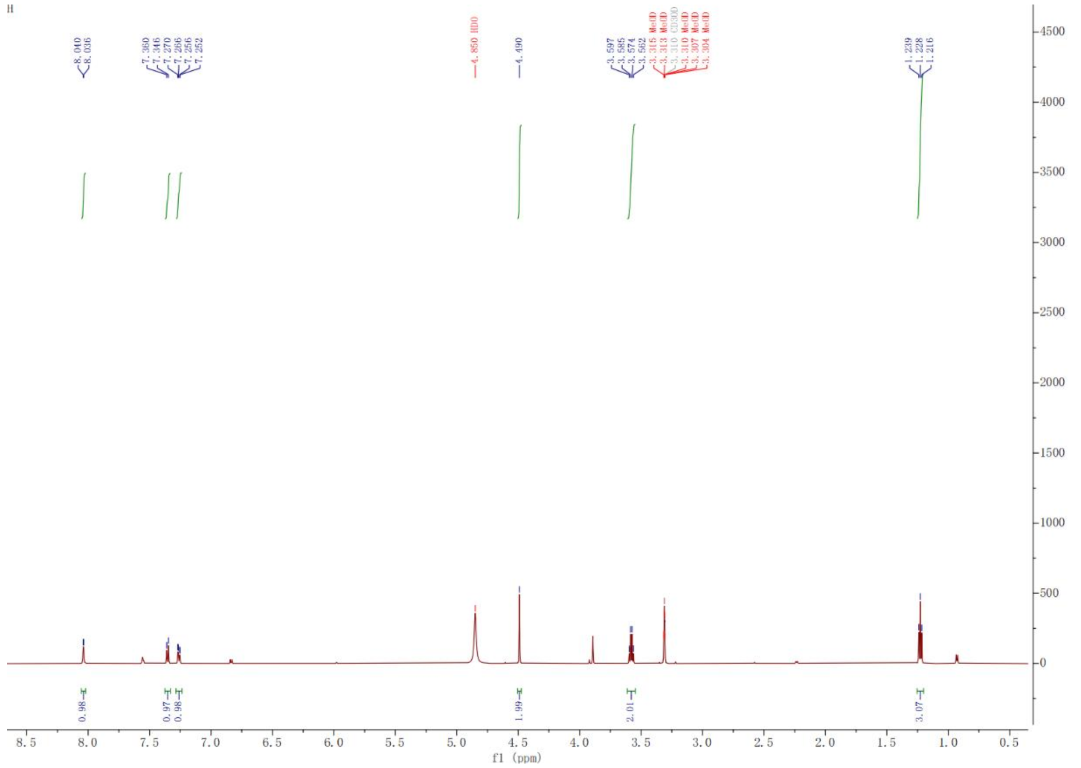

Figure S71. ^{1}H NMR spectrum of **14**.

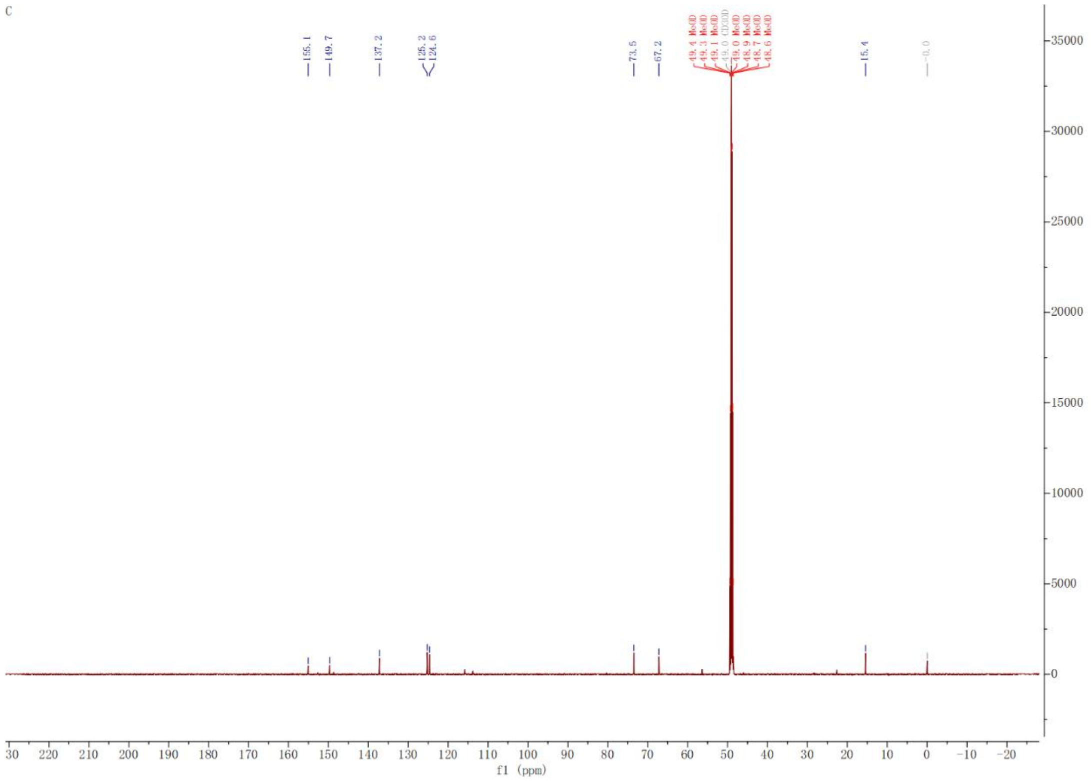

Figure S72. ^{13}C NMR spectrum of **14**.

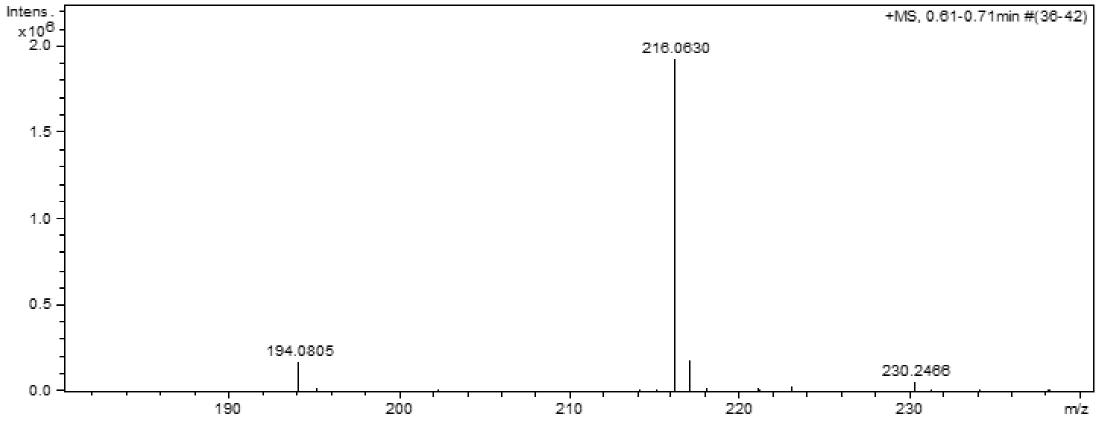

Figure S73. HRESIMS spectrum of **15**.

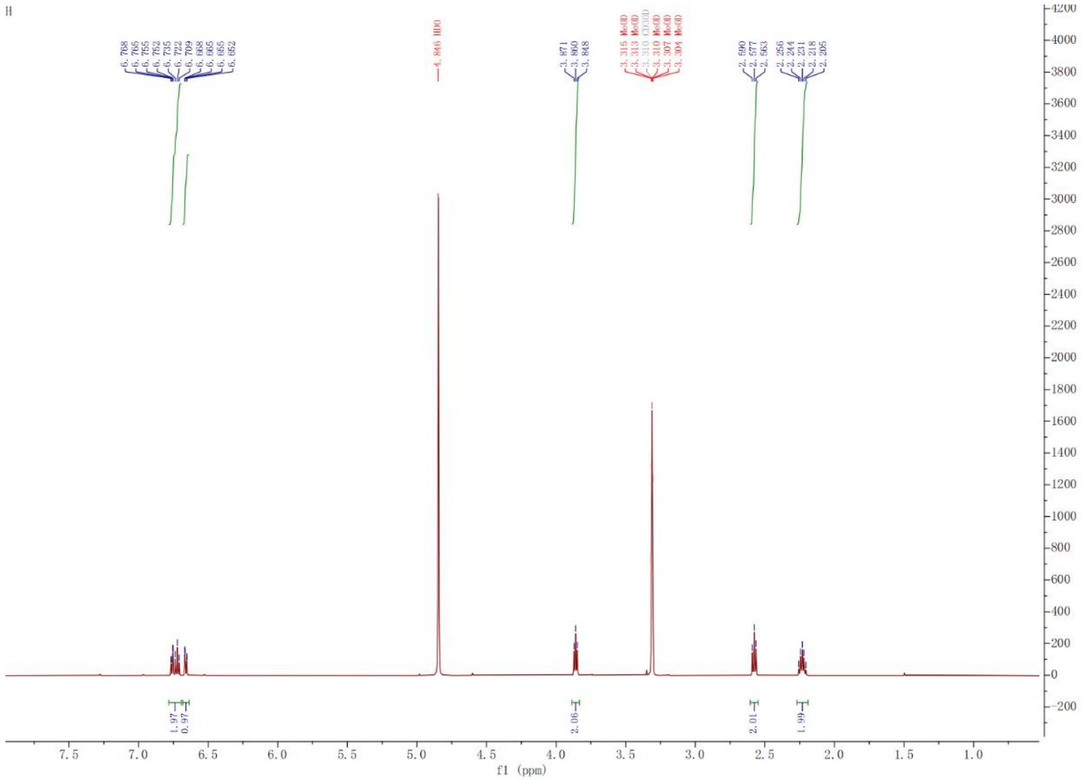

Figure S74. ^{1}H NMR spectrum of **15**.

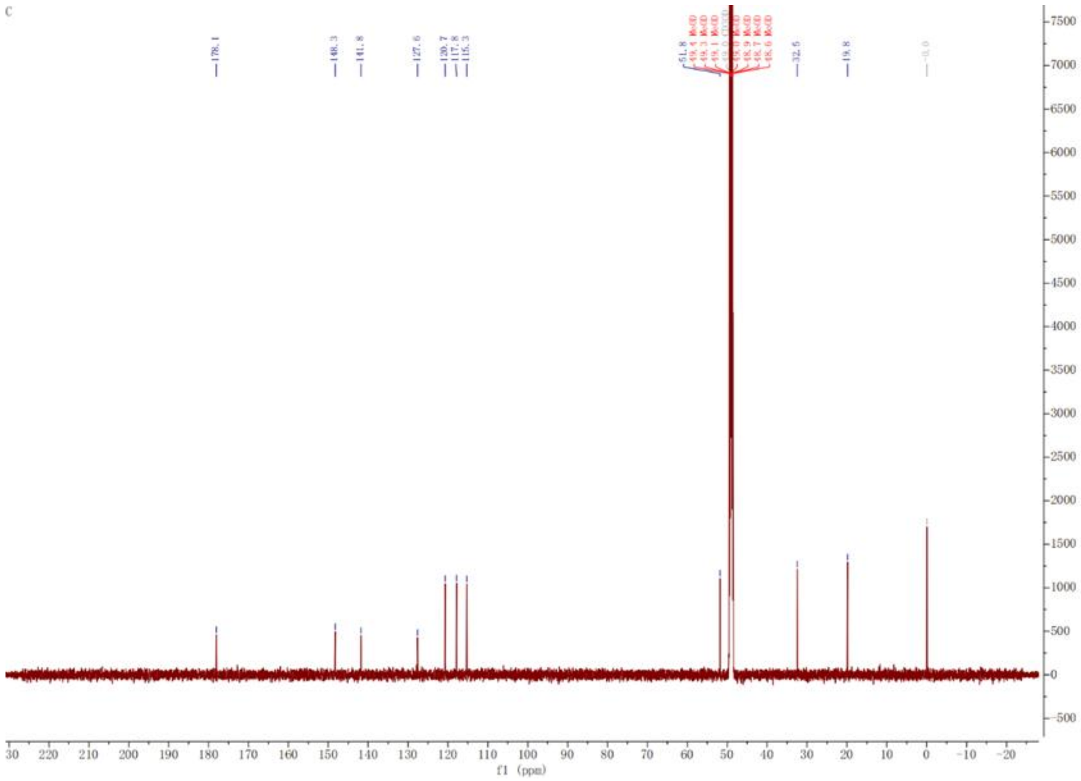

Figure S75. ^{13}C NMR spectrum of **15**.

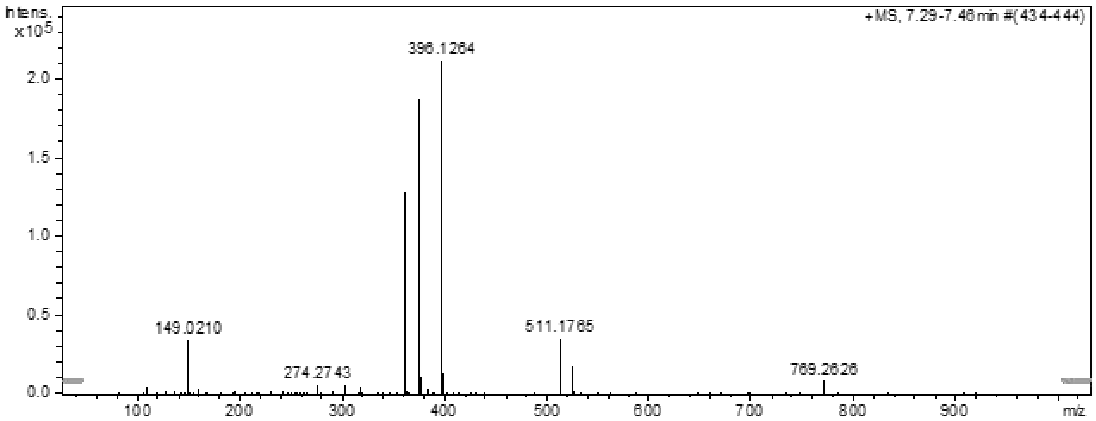

Figure S76. HRESIMS spectrum of **16**.

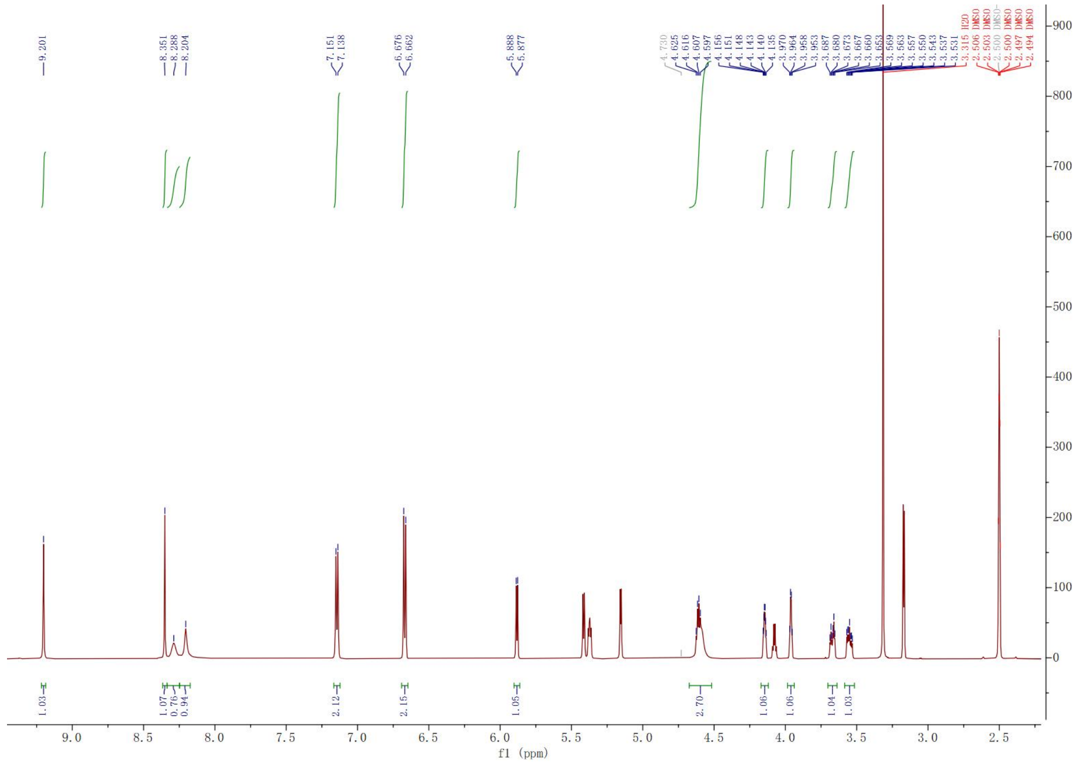

Figure S77. ^{1}H NMR spectrum of **16**.

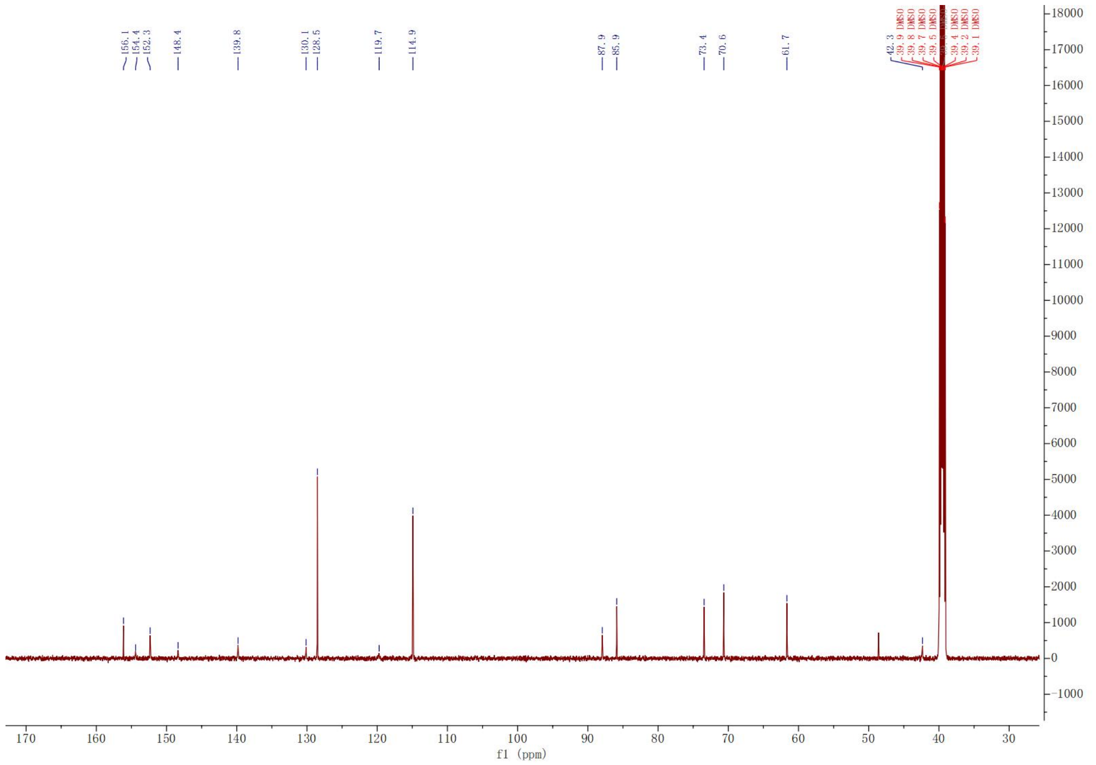

Figure S78. ^{13}C NMR spectrum of **16**.

Table S1 ^{1}H and ^{13}C-NMR date of compounds **1** and **2** recorded in CD_3OD (δ in ppm, J in Hz)

NO.	**1**		**2**	
	δ_H (nH, m, J, Hz)	δ_C (ppm)	δ_H (nH, m, J, Hz)	δ_C (ppm)
1'		133.0		136.9
2', 6'	6.93, d, J = 8.4 Hz	130.7	7.12, d, J = 8.4 Hz	130.8
3', 5'	6.68, d, J = 8.4 Hz	116.1	6.98, d, J = 8.4 Hz	117.6
4'		156.5		157.2
7'	3.76, s	38.8	3.83, s	35.8
1		146.2		129.0
2		144.6		153.8
3	6.89, s	118.8	6.64, d, J = 7.8 Hz	115.8
4		132.6	6.80, dd, J = 7.8, 2.4Hz	128.5
5		132.9		129.5
6	6.65, s	120.4	6.77, d, J = 2.4 Hz	131.9
CH3	2.11, s	19.1	2.16, s	20.6
1''	4.63, d, J = 7.2 Hz	104.7	4.86, s	102.5
2''	3.44, m	74.9	3.42, m	75.0
3''	3.44, m	77.7	3.42, m	78.0
4''	3.44, m	71.1	3.42, m	71.4
5''	3.27, m	78.1	3.37, m	78.1
6''	3.89, dd, J = 12.0, 2.4Hz, H-6''a; 3.70, dd, J=12.6, 4.8 Hz, H-6'''b	62.1	3.88, dd, J = 12.0, 2.4Hz, H-6''a; 3.68, dd, J=12.6, 5.4 Hz, H-6'''b	62.5

Table S2 ^{1}H and ^{13}C-NMR date of compound **13** recorded in CD_3OD (δ in ppm, J in Hz)

NO.	13	
	δ_H (nH, m, J, Hz)	δ_C (ppm)
2	8.04, s	145.6
4		148.2
5		123.9
6		156.6
8	8.14, s	140.1
1'		127.0
2', 6'	7.16, d, J = 8.4 Hz	129.2
3', 5'	6.71, d, J = 8.4 Hz	115.4
4'		157.1
7'	5.22, s	46.2

Table S3 ^{1}H and ^{13}C-NMR date of compounds **17** recorded in CD_3OD (δ in ppm, J in Hz)

NO.	**17**	
	δ_H (nH, m, J, Hz)	δ_C (ppm)
1	-	175.7
2	-	81.4
3	3.09, d, J=17.4 Hz; 3.01, d, J = 17.4 Hz	43.6
4	-	173.9
5	1.78, m; 1.70, m	49.4
6	1.85, m	24.8
7	0.87, d, J = 6.6 Hz	24
8	0.95, d, J = 6.6 Hz	24.8
9	3.75, s	52.8
1'	4.89, s	100.1
2'	3.21, t, J = 7.8 Hz	75.5
3'	3.06, m	77.4
4'	3.40, t, J = 9.0 Hz	70.7
5'	3.35, t, J = 8.4 Hz	78.5
6'	3.77, dd, J = 12.0, 2.4 Hz, H-6'a; 3.69, dd, J = 12.0, 4.2 Hz, H-6'b	62

Table S4 The primer sequence of genes for qPCR

Gene	Sequence of the primer	Length of products (bp)
Col27a1	F: 5'-CGAACAGAAGATGGCGGATGGT-3'<br>R: 5'-CCGTGATAGGCTTCAGGCAAGT-3'	122
Banp	F: 5'-GCCAGCCAGGAAGTCAGAACA-3'<br>R: 5'-CCTCACGGTGGAACAGGTAGTC-3'	219
Hmgcs1	F: 5'-ACGGTTCCCTTGCTTCTGTTCT-3'<br>R: 5'-ATCCTGGTGTGGCATCTTGTGT-3'	138
Insig1	F: 5'-ATCACCATCGCCTTCCTAGCC-3'<br>R: 5'-CCACTGTGACACCTCCTGAGAA-3'	136
Sytl3	F: 5'-CTCCTCCATCCAGTCCAACACA-3'<br>R: 5'-GGCAGCAGGTAGGTCTTGACAT-3'	256
Gsta5	F: 5'-CCTTGTAGGCAACAGGCTGAC-3'<br>R: 5'-GGCTGCTGATTCTGCTCTTGA-3'	131
Egr1	F: 5'-CTGGAGGAGATGATGCTGCTGAG-3'<br>R: 5'-TGCTGCTGCTGCTGCTGTTAT-3'	100
Armcx5	F: 5'-ACAGAACTCCTGGCAGAACCT-3'<br>R: 5'-CCTTGACAGATGGCTCACCTC-3'	127
GAPDH	F: 5'-AAGTTCAACGGCACAGTCAAG-3'<br>R: 5'-ACATACTCAGCACCAGCATCA-3'	122
